# Pre‐ and Post‐Natal Stress Programming: Developmental Exposure to Glucocorticoids Causes Long‐Term Brain‐Region Specific Changes to Transcriptome in the Precocial Japanese Quail

**DOI:** 10.1111/jne.12387

**Published:** 2016-05-25

**Authors:** V. Marasco, P. Herzyk, J. Robinson, K. A. Spencer

**Affiliations:** ^1^ Institute of Biodiversity, Animal Health and Comparative MedicineUniversity of GlasgowGlasgowUK; ^2^ Institute of Molecular Cell and Systems BiologyUniversity of GlasgowGlasgowUK; ^3^ Glasgow PolyomicsWolfson Wohl Cancer Research CentreUniversity of GlasgowGlasgowUK; ^4^ School of Psychology and NeuroscienceUniversity of St AndrewsSt AndrewsUK

**Keywords:** early‐life stress, pre‐ and post‐natal glucocorticoid exposure, brain transcriptome, hippocampus, hypothalamus

## Abstract

Exposure to stress during early development can permanently influence an individual's physiology and behaviour, and affect its subsequent health. The extent to which elevated glucocorticoids cause such long‐term ‘programming’ remains largely untested. In the present study, using the Japanese quail as our study species, we independently manipulated exposure to corticosterone during pre‐ and/or post‐natal development and investigated the subsequent effects on global gene expression profiles within the hippocampus and hypothalamus upon achieving adulthood. Our results showed that the changes in transcriptome profiles in response to corticosterone exposure clearly differed between the hippocampus and the hypothalamus. We also showed that these effects depended on the developmental timing of exposure and identified brain‐region specific gene expression patterns that were either: (i) similarly altered by corticosterone regardless of the developmental stage in which hormonal exposure occurred or (ii) specifically and uniquely altered by either pre‐natal or post‐natal exposure to corticosterone. Corticosterone‐treated birds showed alterations in networks of genes that included known markers of the programming actions of early‐life adversity (e.g. brain‐derived neurotrophic factor and mineralocorticoid receptor within the hippocampus; corticotrophin‐releasing hormone and serotonin receptors in the hypothalamus). Altogether, for the first time, these findings provide experimental support for the hypothesis that exposure to elevated glucocorticoids during development may be a key hormonal signalling pathway through which the long‐term phenotypic effects associated with early‐life adversity emerge and potentially persist throughout the lifespan. These data also highlight that stressors might have different long‐lasting impacts on the brain transcriptome depending on the developmental stage in which they are experienced; more work is now required to relate these mechanisms to organismal phenotypic differences.

The responses of an organism to early‐life environments can have long‐term phenotypic effects, potentially persisting for the whole lifespan 1. In vertebrates, such early‐life phenotypic ‘programming’ or ‘priming’ can come about as a result of environmental factors acting directly on the developing individual, or indirectly, often via the mother 2. Subsequent to the pioneering clinical observations of Barker *et al*. 3 showing links between low birth weight and an increased propensity to adult diseases, a great deal of work has focused on the long‐term phenotypic consequences induced by early‐life adversity. It is generally assumed that adverse developmental environments, which often expose the developing organisms to physiologically stressful conditions, are likely to be damaging for organismal fitness and predispose individuals to an increased risk of adult morbidity and mortality 4. There is, however, a growing body of experimental work on a range of vertebrate taxa suggesting that exposure to challenging environments in early life can have both positive and negative consequences on fitness outcomes 5, 6. On the positive side, early‐life adversity may induce preparative physiological responses (i.e. predictive adaptive responses) that could prime the growing individual to better cope with future environmental conditions 7. However, long‐term costs may still arise as a result of unavoidable negative side effects or because of a mismatch between the expected and the encountered environmental conditions 1. Matching and mismatching challenges may have an especially important adaptive role when they occur in early life 8. Indeed, recent experimental work indicates that adult phenotypic variation on behavioural and physiological traits is also a function of the quality of the environments experienced across differing developmental periods 9, 10, 11, 12.

The mechanisms underlying such phenotypic programming remain elusive. One of the main candidate mechanisms is the overexposure to glucocorticoid stress hormones that often occurs when animals are subjected to a wide range of both pre‐ and/or post‐natal stressors 4, 13. The hypothalamic‐pituitary‐adrenal (HPA) axis regulates both the production and secretion of glucocorticoids. These steroids bind to specific nuclear receptors [i.e. mineralocorticoid (MR) and glucocorticoid (GR) receptors] that are expressed throughout the brain and act as transcription factors 14. The hypothalamus and hippocampus, key regulatory brain regions within the HPA axis, are both particularly sensitive to the actions of early‐life glucocorticoid exposure and express high concentrations of MR and GR receptors 14, 15, 16. In laboratory rats, pre‐natal stressors have been shown to decrease hippocampal MR and GR receptors 17, 18 and to increase the turnover of brain noradrenergic neurones into adulthood 19. Long‐term changes in glucocorticoid receptors in the brain in response to pre‐ and post‐natal stressors have also been demonstrated in other vertebrate species, such as birds 20, 21. However, a growing line of evidence now suggests that the effects of early‐life adversity in the brain are unlikely limited to single specific genetic targets. For example, global gene expression patterns (i.e. transcriptome profiles) were significantly altered in the hippocampus of adult rats as a function of variation in maternal care 22. In chickens, a variety of early‐life adversities such as unpredictable light/dark rhythm, unpredictable access to food and intermittent social isolation, which are presumed to be associated with increased glucocorticoid levels, resulted in significant changes in the neural gene expression signature and similar directional changes were also observed in the offspring 23, 24, 25. The effects of these types of stressors on the brain transcriptome might be driven by the direct exposure to elevated glucocorticoids during development. To date, this idea remains experimentally largely untested. We also do not know the extent to which the effects of pre‐natal stressors on the brain transcriptome might differ from those induced by post‐natal stressors. Disentangling this experimentally requires independent manipulations during pre‐natal and post‐natal development.

The main aim of the present study was to examine the potential long‐term effects of the independent and combined exposure to exogenous corticosterone (main glucocorticoid in birds) during pre‐ and post‐natal development on brain‐region‐specific transcriptome profiles within the hippocampus and hypothalamus. As our study species, we used the precocial Japanese quail, which ensured that controlled experimental manipulations were undertaken during individuals’ development without the mother potentially compensating for them, given that the fertile eggs were artificially incubated and no maternal care was provided to the chicks. In the Japanese quail, maximal brain growth and maturation takes place during the pre‐natal stages of development, similar to other precocial mammalian species such as the spiny mouse and guinea pigs. This is, however, in contrast to other mammalian models, such as mice and rats, which have altricial developmental modes and show relatively delayed neuronal developmental processes. Using our chosen precocial study species, we aimed to test three main predictions. First, that both pre‐ and post‐natal exposure to corticosterone would induce similar alterations in the brain transcriptome across all the stress‐exposed birds compared to the controls. Second, that the combined effects of both pre‐ and post‐natal protocols would induce additive/cumulative gene expression changes as a result of ‘potentiated’ induced‐phenotypic plasticity in the quail that experienced both pre‐ and post‐natal exposure to corticosterone compared to those exposed to the stressful treatment during either pre‐ or post‐natal development 8. In mammalian species, developmental timelines have been shown to be important contributory factors when analysing the effects of early‐life stressful events in the brain 16. Also, pre‐natal stressors can induce different effects on physiology and behaviour compared to post‐natal stressors 9, 10, 11, 12. Therefore, our third prediction was that certain long‐term alterations in the brain transcriptome would be specifically and uniquely dependent on either pre‐natal corticosterone or post‐natal corticosterone exposure.

## Materials and methods

### Animals and treatments

The Japanese quail (*Coturnix coturnix japonica*) used in the present study were also used in previous work to determine the short‐ and long‐term effects of early‐life exposure to glucocorticoids on the physiological phenotype (full details of experimental procedures and housing conditions have been reported previously) 10, 11. Briefly pre‐natal stress was mimicked by a single injection of corticosterone (B) into the yolk on day 5 of incubation. B‐treated eggs were injected at the conical tip with 10 μl of a sterile solution of B (Sigma‐Aldrich, St Louis, MO, USA; concentration B: 850 ng/ml; dose: 8.5 ng), whereas control eggs were injected with 10 μl of vehicle alone. This B dose elevated endogenous yolk B concentrations within 1.8 SD above the control yolks, which was determined using both a radioimmunoassay and liquid chromatography‐mass spectroscopy 12. This increase of B is similar to previous avian work that manipulated B concentrations in the egg within the relevant physiological ranges (e.g. 26–27). Day 5 of incubation was chosen because this is the earliest point at which egg fertility can be reliably determined in the Japanese quail. Avian egg yolks are stratified in layers at laying and these layers break down after a few days of incubation, with the yolk becoming mixed. Our injection at day 5 therefore ensured that B levels were increased once yolk layers had ceased to exist. In the chicken, embryonic production of B is detected from around pre‐natal day 8 (total incubation time of 21 days). However, embryonic B production at this time is via basal secretion by the adrenal glands and not under the influence of stress given that the HPA axis is not fully functional until the second half of incubation, from pre‐natal day 14 28. Therefore, because our pre‐natal protocol was performed within the first half of incubation (total incubation time 17–19 days), the experimental elevation of B was meant to predominantly reflect maternal stress. Hatched quail from each pre‐natal treatment were subsequently randomly allocated to one of two post‐natal treatment protocols: post‐natal B treatment or post‐natal control treatment. Age‐matched quail were housed in four different treatment‐specific enclosures. A brooding lamp was placed over each enclosure to ensure an appropriate initial brooding temperature of 35.5 °C, which was gradually decreased by 1–1.5 °C until post‐natal day 19 when warming bulbs were switched off and the juveniles were subjected to the constant ambient temperature of 19 °C. Birds allocated to the post‐natal B treatment were administered with a unique daily oral dose of B from post‐natal day 5 to post‐natal day 19 (B dose: 0.045 mg/day between post‐natal day 5 and post‐natal day 15 and 0.09 mg/day between post‐natal day 16 to post‐natal day 19), whereas birds allocated to the post‐natal control treatment were given a single daily oral dose of vehicle alone. Doses were delivered using mealworms (*Tenebrio molitor*) injected with 10 μl of B solution dissolved in peanut oil (concentration B: 4.5 mg/ml between post‐natal day 5 and post‐natal day 15, and 9 mg/ml between post‐natal day 16 and post‐natal day 19) or with 10 μl of peanut oil alone. To confirm that each juvenile quail was eating a single mealworm/day, chicks housed in the same age treatment‐specific enclosure were separated with transparent dividers during feeding. The post‐natal B dosages were validated in previous work (10) and were within the relevant physiological ranges. Thus, there were four groups of experimental birds: (i) pre‐natal and post‐natal control birds (CC, n: female = 9, male = 14); (ii) pre‐natal B‐treated and post‐natal control birds (BC, n: female = 9, male = 6); (iii) pre‐natal control and post‐natal B‐treated birds (CB, n: female = 10, male = 10); and (iv) pre‐natal B treated and post‐natal B treated birds (BB, n: female = 9, male = 8).

At post‐natal day 19, the juvenile quail were sexed by sexual dimorphic plumage and singly housed in 61 × 46 × 51 cm enclosures, in visual and auditory contact with conspecifics. Food (turkey starter crumbs; Dodson and Horrell, Kettering, UK) and water were available *ad lib*. Upon reaching adulthood, post‐natal days 69–73, the birds were sacrificed by i.p. administration of 2 ml of Euthatal (sodium pentobarbital; 200 mg/ml; Merial Animal Health, Harlow, UK). All procedures were carried out under Home Office Project Licence 60/4068 and Personal Licence 60/12436.

### Tissue collection

*Post mortem* (within 1 min after administration of Euthatal), birds were decapitated, and brains subsequently removed within (mean ± SEM) 10.57 ± 0.20 min *post mortem*. Dissected brains were immediately placed on dry ice and stored at −80 °C for further dissection. To perform the dissections, the brains were placed ventral side up into a frozen custom made brain holder (Workshop, University of Glasgow, UK) with a 1‐mm graduated scale, and a 2‐mm thick coronal section was then obtained using two razor blades positioned approximately 4 mm from the rostral pole and 2 mm from the cerebellum. The cutting plane was adjusted to match as closely as possible the plane of the chicken brain atlas (29) (coronal brain sections interaural 2.08–2.56 mm), such that tissue collection was standardised across animals. Then, two equivalent bilateral punches (each with a diameter of 1 mm) were obtained from the hippocampus and one single (diameter 2 mm) was obtained from the basal hypothalamus that spanned the third ventricle. The obtained hippocampal and hypothalamic punches were then stored at −80 °C until analysis.

### RNA isolation and high‐throughput RNA‐sequencing

Total RNA was extracted using the Rneasy Microarray Tissue Mini Kit (Qiagen, Manchester, UK), with a DNase digestion step to remove DNA contaminants using RNase‐Free DNase Set (Qiagen). Both purity and integrity of RNA were assessed, respectively, using a Nanodrop spectrophotometer (Thermo Fisher Scientific, Wilmington, DE, USA) and Agilent 2100 Bioanalyzer (Agilent Technologies, Santa Clara, CA, USA). Hippocampal and hypothalamic RNA concentrations (mean ± SEM) were 158.86 ± 6.89 ng/μl and 193.06 ± 6.39 ng/μl, respectively; RNA integrity number (RIN) scores for the hippocampal and hypothalamic samples (mean ± SEM) were 8.84 ± 0.06 and 9.16 ± 0.06, respectively.

Total RNA (i.e. RIN ≥ 8) extracted from the hippocampi and hypothalami of 48 randomly selected quail were used for the RNA‐sequencing (RNA‐seq). For each tissue, three RNA biological replicates were prepared by pooling RNA from four birds (two males and two females) per treatment. Each pool contained the same amount of RNA from each individual bird (500 ng each; 2000 ng in total). The same pools of individuals were used for each tissue. Birds sharing the same mother were avoided where possible within the same pool to control for potential pseudoreplication.

The RNA pooled samples were then processed for RNA‐seq using standard TruSeq™ RNA Sample Preparation kit (Illumina, Little Chesterford, UK). Sequencing was performed on a Genome Analyzer IIX (GAIIX) platform at the Glasgow Polyomics facility (University of Glasgow, UK) using standardised procedures; full details are provided in paragraph 1.1, Supporting information. The sequencing run terminated after 76 cycles and yielded single‐end reads with a maximum length of 76 bases.

### Quantitative polymerase chain reaction (qPCR)

Using the same hippocampal RNA pools previously used for the RNA‐seq, we also performed qPCR to measure gene expression for three selected genes showing relatively high fold changes in at least one treatment pair‐wise comparison in the RankProducts analysis: transthyretin (TTR); superoxide dismutase extracellular 3 (SOD3); and guanine nucleotide binding protein (G‐protein), Gamma 11 (GNG11) (full methodological details are provided in paragraph 1.2, Supporting information).

### Data analysis

#### Mapping of the RNA‐seq reads

After standard quality check of the raw sequencing data (full details are provided in paragraph 1.3, Supporting information), the quail reads were aligned to the chicken (*Gallus gallus*) genome. This reference was chosen because: (i) there is a high degree of conservation between the quail and the chicken genome as confirmed by comparative mapping of macrochromosomes 30 and (ii) it provides the best quantitative annotation in comparisons with the zebra finch and the turkey. A recent study suggested that direct genome mapping outperforms *de‐novo* based methods 31. We ackowledge the availability of the first assembled draft of the Japanese quail genome 32; however, lack of annotation file (e.g. GFF/GTF format) and transcriptome nucleotide sequence still make the use of these data difficult for RNA‐sequencing analysis.

Preliminary alignments of our generated quail reads onto the chicken genome were performed to establish a reasonable compromise between the high number of aligned reads and the small number of reads mapped to more than one location in the genome (full details are provided in paragraph 1.4, Supporting information). The final alignment was performed using TopHat version 1.3.2 33 using 36 bases long reads. TopHat was provided with: (i) The genomic sequence of the chicken reference genome (FASTA file ‘galGal3’; http://genome.ucsc.edu; International Chicken Genome Sequencing Consortium (2004), May 2006 release) and (ii) the chicken genome annotation (WASHUC2 release‐65, GTF file *Gallus gallus*; http://www.ensembl.org). For the final TopHat runs, the default parameters were used, except for a few parameters as detailed in Table S1 (Supporting information).

#### Quantification and normalisation of expression signal

htseq, version 0.5.3p9 34 was used to count how many reads map to each gene. The software was provided with (i) the alignment files in BAM format and (ii) the chicken genome annotation in GTF format. The obtained raw counts were then normalised using the 75th percentile of nonzero count distribution within each sample in bayseq, version 1.8.0 (35).

### Differential expression analysis: RankProducts and vector analysis

#### RankProducts

The normalised counts were then transformed to log2 (normalised counts + 32) to minimise deviation from constant variance across all the genes 36. We used principal component analysis (PCA) plots to reduce dimensionality of the data and to identify principal components along which variation of the data is maximal, and to assess similarities and differences among samples. RankProducts was used to detect differentially expressed genes 36, 37 using the ‘RankProd’ package, version 2.28.0 (http://bioconductor.org) in r environment, version 2.14.2 (The R Project for Statistical Computing, Vienna, Austria). The tissue‐specific data were analysed separately using a pairwise approach (i.e. all six contrasts per tissue). RankProducts was carried out using the ‘data from single origin’ option; ranks and P‐values were calculated using 1000 permutations. The analysis controlled for the multiple testing error using the percentage of false‐positives, which estimates the false discovery rate (FDR) 38.

#### Vector analysis

**Figure 1:**
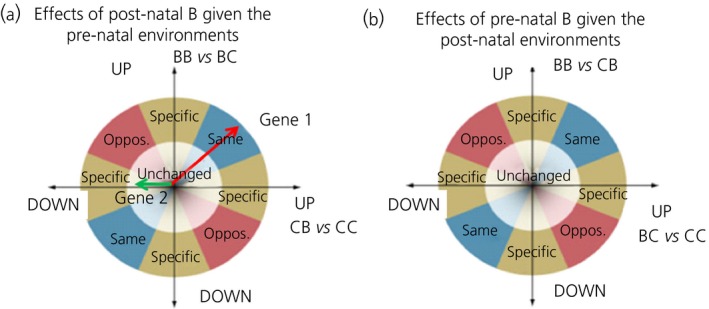
Graphical representation, modified from Breitling *et al*. (39) of the vector analysis performed to examine (a) the long‐term responses of post‐natal corticosterone (B) exposure given the pre‐natal environments [i.e. control (horizontal axis) or exposure to B (vertical axis)] and (b) the long‐term responses of pre‐natal B given the post‐natal environments (i.e. control or exposure to B). On the two axes are reported the log2‐fold changes of genes in response to the developmental environments. (a) |Vsum| of two hypothetical vectors are reported: gene 1 (in red) is strongly up‐regulated in both the environments (vector analysis class: 0 up, 1 up), whereas gene 2 (in green) is specifically and weekly down‐regulated in the control environment (vector analysis class: 1 down, 0 unchanged). (a, b) The Cartesian plane is systematically subdivided into sectors corresponding to the following prototypical behaviours: genes that show inconsistent responses in either environments (unchanged, in white); genes that show similar responses in both environments (in blue); genes that show opposite (oppos.) responses in both environments (in red); and, finally, genes that are specifically down‐regulated in one environment and not in the other one (in yellow).

**Figure 2:**
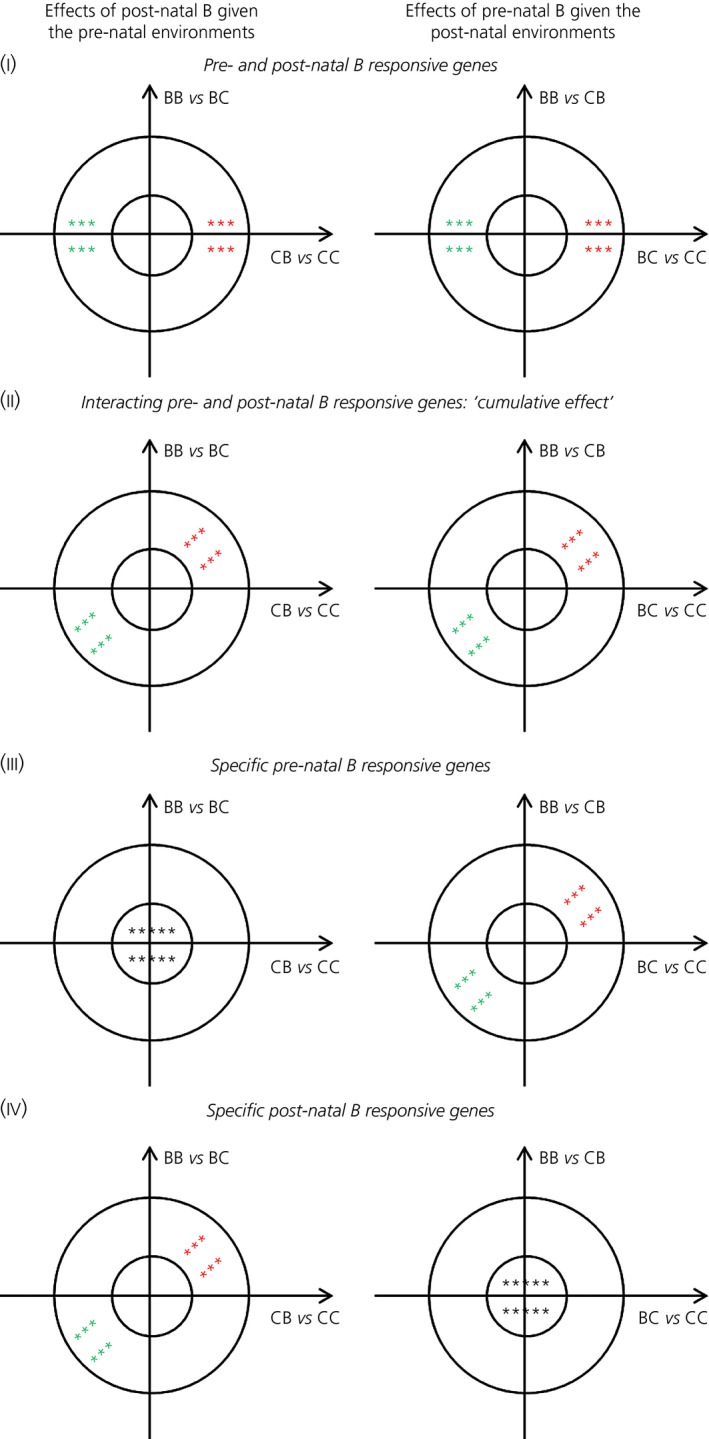
Illustration of the behavioural categories (I, II, III, and IV) used to filter the vector analysis results. The stars represent the vectors |Vsum| of hypothetical genes: in red or green, respectively, the significant up‐regulated or down‐regulated genes in one or both the pre‐ and post‐natal environments (|Vsum| ≥ 40 and P* *≤* *0.05); in black, the genes whose responses were not significant specifically in one environment (|Vsum| ≤ 40 and P ≥ 0.05).

Vector analysis 39 was used to further process the results from the RankProducts analysis. Here, the difference in the response of each gene compared to either post‐natal or pre‐natal treatment with B was represented by a vector in a Cartesian plane where the orthogonal axes correspond to two developmental environments, namely pre‐ and post‐natal. Subsequently, we established the gene behaviour depending on the size and orientation of such vector after vector summation was applied to all between‐replicate comparisons. Therefore, we quantified the extent to which the expression of a given gene was modified in the adult quail by the independent or combined exposure to pre‐ and post‐natal B within the different developmental environments. We performed two separate analyses in each tissue using genes filtered with FDR ≤ 0.10 criterion in the preceding RankProducts calculations applied to all six contrasts (i.e. 489 genes in the hippocampus and 302 genes in the hypothalamus). These two analyses were then compared in pairs (Fig. 1). The first analysis focused on interrogating the long‐term responses of post‐natal B given the pre‐natal environments: (i) ‘pre‐natal exposure to B’ using the contrast BB versus BC or (ii) ‘pre‐natal exposure to carrier only’ using the contrast CB versus CC. The second analysis focused on interrogating the long‐term responses of pre‐natal B given the post‐natal environments: (i) ‘post‐natal exposure to B’ using the contrast BB versus CB or (ii) ‘post‐natal exposure to carrier only’ using the contrast BC versus CC. The resulting data were then decomposed into classes according to the orientation of the representative vector and filtered using |Vsum| = 40 and P* *=* *0.05 as cut‐off values to keep statistically significant and high consistency data (Fig. 2). These cut‐offs are indeed much more stringent compared to those used in the original vector analysis study 39. To confirm our three main predictions, we specifically focused analyses on the following four behavioural categories (full details on algorithm definitions are provided in the Supporting information, Table S2):

Category I *–* Pre‐ and post‐natal B responsive genes: the genes that showed similar significant long‐term responses in both the pre‐ and post‐natal environments as a consequence of exposure to B regardless of the developmental stage in which the stressful treatment occurred (Fig. 2‐I). These genes corresponded to those that were (i) significantly and similarly up‐ or down‐regulated across all the B‐exposed birds (i.e. BC, CB and BB) relatively to the controls (i.e. CC) and (ii) showed no significant cumulative effects of pre‐ and post‐natal B;
Category II *–* Interacting pre‐ and post‐natal B responsive genes (cumulative effect): the genes that showed (i) similar significant long‐term responses to post‐natal B in both the pre‐natal environments and (ii) similar significant long‐term responses to pre‐natal B in both the post‐natal environments. These genes corresponded to those that were altered (i) by both pre‐ and post‐natal B (as in Category I) and (ii) by cumulative effects of pre‐ and post‐natal B via elevating or attenuating expression depending on whether the fold changes BB/BC, BB/CB, BC/CC and CB/CC were all positive and similar, or all negative and similar (Fig. 2‐II);
Category III *–* Specific pre‐natal B responsive genes: the genes that showed (i) no response to post‐natal B in either pre‐natal environments and (ii) similar significant responses to pre‐natal B in both the post‐natal environments (Fig. 2‐III);
Category IV *–* Specific post‐natal B responsive genes: the genes that showed (i) similar significant responses to post‐natal B in both the pre‐natal environments and (ii) no response to pre‐natal B in either post‐natal environments (Fig. 2‐IV).

### Functional analysis

We used ingenuity pathway analysis (IPA) (http://www.ingenuity.com/products/ipa) to identify biological processes and molecular networks associated with the differentially expressed significant gene lists identified according to the vector analysis behavioural categories. Functional analyses in IPA were concordant with analyses using DAVID bioinformatics resources (https://david.ncifcrf.gov/) (data not shown).

Ingenuity pathway analysis was performed only on the mostly populated gene lists within each tissue. In total, we performed two analyses: one by combining together the hippocampal genes in the behavioural category I and II, and the second one using the hypothalamic gene list in the behavioural category III. The grouping of the hippocampal gene lists belonging to categories I and II was biologically relevant because we incorporated together the genes that were similarly altered by both early‐life stressful treatments (i.e. all these genes were significantly up‐ or down‐regulated across the B‐exposed birds compared to the controls). In IPA, each gene was mapped to its corresponding gene object in the ingenuity knowledge base (IKB) via ortholog mapping (to their vertebrate counterparts, mostly human, mouse and rat). The IKB converted each gene list into a shorter dataset of well‐characterised, nonredundant ‘focus’ genes. These genes were then mapped to molecular networks in IKB on the basis of their inter‐connectivity and ranked by scores, indicating the probability that a collection of genes equal to or greater than the number in a network was a result of chance alone.

## Results

### Raw data quality control

In both the hippocampal and hypothalamic samples, the accuracy of the reads was high overall throughout the sequencing runs (full details on the Phred quality distributions per sequencing cycle for all the 24 samples are provided in the Supporting information, Fig. S1).

### Read alignment

Full details on key alignment metrics extracted from the TopHat outputs are provided in the Supporting information (Table S3). Briefly, between 56% and 62% of reads mapped to the reference genome, and between 52% and 57% represented unique hits. The number of reads spanning the predicted splice junctions corresponded to 6.4–7.2% of all mapped reads.

### PCA

The PCA plot of all the RNA‐seq samples clearly showed two tissue‐specific expression patterns along PC1 (see Supporting information, Fig. S2). Separation of the hippocampal samples along the three PCs was less clear in both the hippocampal and hypothalamic samples (see Supporting information, Fig. S3). However, the PCA plot with only the hippocampal samples suggested a relatively good separation along PC3 across all the B‐treated birds compared to the controls (see Supporting information, Fig. S3a), suggesting an overall effect of the treatment on hippocampal gene expression. The PCA plot with only the hypothalamic samples suggested a relatively good separation among CC, BC and BB samples along both PC1 and PC2 (see Supporting information, Fig. S3b).

### RankProducts

The number of significant candidate genes revealed by RankProducts across all six pairwise contrasts in both the hippocampal and hypothalamic samples is shown in Table 1. The top 20 up‐ and down‐regulated genes for each comparison in both the tissues are provided in the Supporting information (Table S4).

**Table 1:** Number of Significant Genes (False Discovery Rate ≤ 0.10) that were Up‐ or Down‐ Regulated Across the RankProducts Pairwise Comparisons in the Hippocampus and Hypothalamus

Contrast	Up‐regulated genes	Down‐regulated genes
(1st class versus 2nd class)	Under 1st class	Under 1st class
Hippocampus		
BC versus CC	159	231
CB versus CC	3	21
BB versus CC	19	127
CB versus BC	4	10
BB versus BC	53	43
BB versus CB	43	2
Hypothalamus		
BC versus CC	56	32
CB versus CC	21	5
BB versus CC	159	22
CB versus BC	24	31
BB versus BC	117	19
BB versus CB	10	3

CC, pre‐natal and post‐natal control birds (CC); BC, pre‐natal B‐treated and post‐natal control birds; CB, pre‐natal control and post‐natal B‐treated birds; BB, pre‐natal B treated and post‐natal B treated birds.

#### Hippocampus

In the hippocampus, the adult birds that experienced exposure to B during only pre‐natal development showed the largest number of differentially expressed candidate genes in comparison to the controls (i.e. BC versus CC) (Table 1); the differences were skewed towards a repression of gene expression (231 down‐regulated genes and 159 up‐regulated genes) with fold changes for the down‐regulated genes ranging from −18.2 to −1.5 (see Supporting information, Table S4a). The birds that experienced both pre‐ and post‐natal stressful treatments also showed a relatively large number of differentially down‐regulated genes compared to control birds (i.e. 127 down‐regulated genes versus 19 up‐regulated genes) with fold changes for the down‐regulated genes ranging from −28.1 to −1.6 (see Supporting information, Table S4a).

#### Hypothalamus

In the hypothalamus, the comparisons between BB versus CC and BB versus BC showed the highest number of significantly expressed candidate genes (i.e. BB versus CC: 22 down‐regulated genes and 159 up‐regulated genes; BB versus BC: 19 down‐regulated genes and 117 up‐regulated genes) and, in contrast to what was observed in the hippocampus, the transcriptome differences were skewed towards an overall increase in gene expression changes in the BB birds relative to CC or BC birds with fold changes ranging from 5.1 to 1.4 (see Supporting information, Table S4b).

### Vector analysis behavioural categories

**Figure 3:**
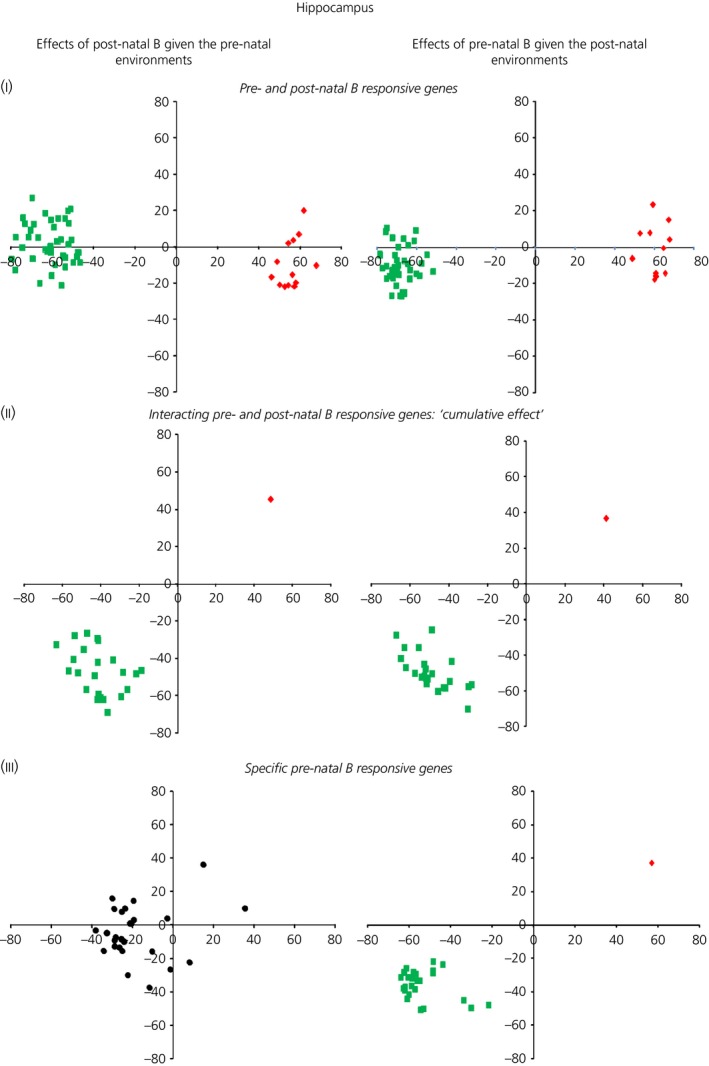
Significant up‐regulated (red) and down‐regulated (green) genes: in black, unchanged (nonstatistically significant differentially expressed genes) within the hippocampus filtered according to the behavioural categories I, II and III.

**Figure 4:**
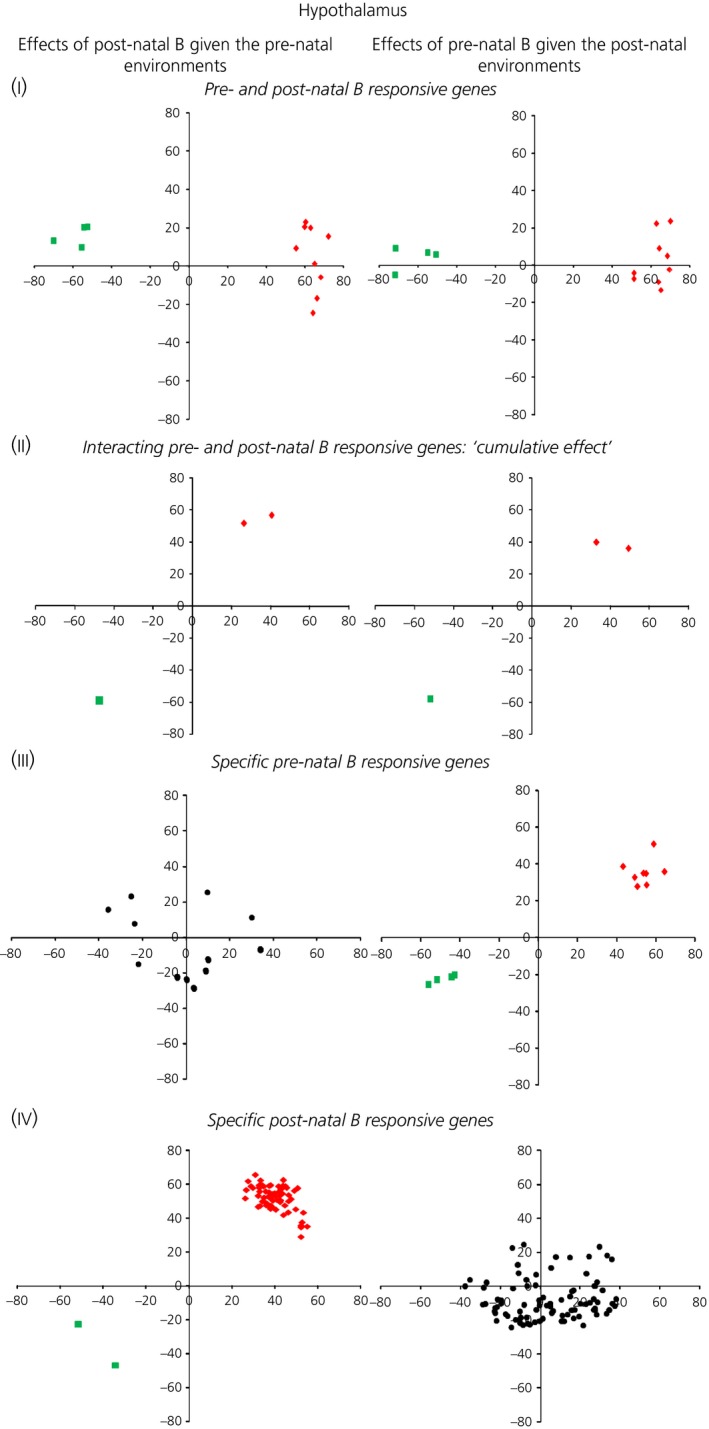
Significant up‐regulated (red) and down‐regulated (green) genes: in black, unchanged (nonstatistically significant differentially expressed genes) within the hypothalamus filtered according to the behavioural categories I, II, III and IV.

A graphical representation of vector analyses data before partition into behavioural categories is provided in the Supporting information (Fig. S4). The number of genes that were filtered according to our vector analysis behavioural categories is reported in Table 2 and Figs 3 and 4 (a complete gene list per each category is provided in the Supporting information, Table S5).

**Table 2:** Number of Genes Belonging to Each Behavioural Category in the Hippocampal and Hypothalamic Samples

Behavioural category	Hippocampus	Hypothalamus
I. Pre‐ and post‐natal B responsive genes	53	13
II. Pre‐and post‐natal B responsive genes: cumulative effect	24	3
III. Pre‐natal B responsive genes	26	12
IV. Post‐natal B responsive genes	0	71

#### Hippocampus

There were 53 genes in which the effects of early‐life stress, regardless of timing, induced similar expression changes in the hippocampus across all the B‐treated phenotypes compared to the controls (Fig. 3‐I, Category I). The changes in expression estimates of such genes were skewed towards a down‐regulation (40 genes were down‐regulated and 13 were up‐regulated). Interestingly, included amongst the up‐regulated genes are the genes encoding MR (nuclear receptor subfamily 3, group C, member 2 or NR3C2), brain‐derived neurotrophic factor (BDNF) and Ca^2+^‐dependent activator protein for secretion 2 (CADPS2); a full gene list is provided in the Supporting information (Table S5a). There were 24 genes in which the combined long‐term effects of pre‐ and post‐natal B induced ‘cumulative’ changes in the hippocampus of the birds that experienced both early stress protocols: 23 genes were down‐regulated and 1 gene was up‐regulated (Fig. 3‐II, Category II). Although there were 26 genes specifically and uniquely regulated by pre‐natal B (Fig. 3‐III, Category III), no genes were specifically regulated by post‐natal B alone (Category IV). The changes observed in these pre‐natal B responsive genes were strongly skewed towards a repression of their expression, with 25 genes down‐regulated and 1 gene up‐regulated. Among these down‐regulated genes, there were hormone receptors, including the melanocortin receptors (type 4 and 5), growth hormone precursor and TTR.

#### Hypothalamus

By contrast to what was observed in the hippocampus, only a few genes (n = 13; 9 of which were up‐regulated and 4 down‐regulated) showed the same dynamic responses to the pre‐ and post‐natal B protocols compared to the controls (Fig. 3‐I, Category I). Cumulative interacting changes in the birds that experienced the combined stress protocols were identified in only 3 genes (2 up‐regulated and 1 down‐regulated) (Fig. 4‐II, Category II). Although relatively few genes (12 genes: 8 and 4 genes were up‐ and down‐regulated, respectively) were specifically regulated by the exposure to B *in ovo* (Fig. 3‐III, Category III), more genes (71 genes: 69 and 2 genes were up‐ and down‐regulated, respectively) were specifically modulated by B administered during the post‐natal development (Fig. 3‐IV, Category IV). Among the genes specifically altered by the post‐natal B protocol, there were hormone receptors implicated in the regulation of serotonin, somatostatin and corticotrophin‐releasing factor (a full gene list is provided in the Supporting information, Table S5b).

### Functional analysis

#### Hippocampus

**Figure 5:**
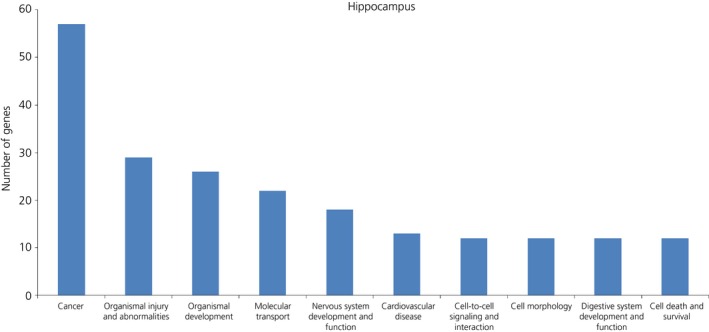
Functional gene ontology using the pre‐ and post‐natal B responsive and cumulative B genes in the hippocampus (vector analysis behavioural category I and II combined together). The top 10 functional groups with larger number of genes were categorised using Ingenuity Pathway Analysis software (IPA; http://www.ingenuity.com/products/ipa).

**Figure 6:**
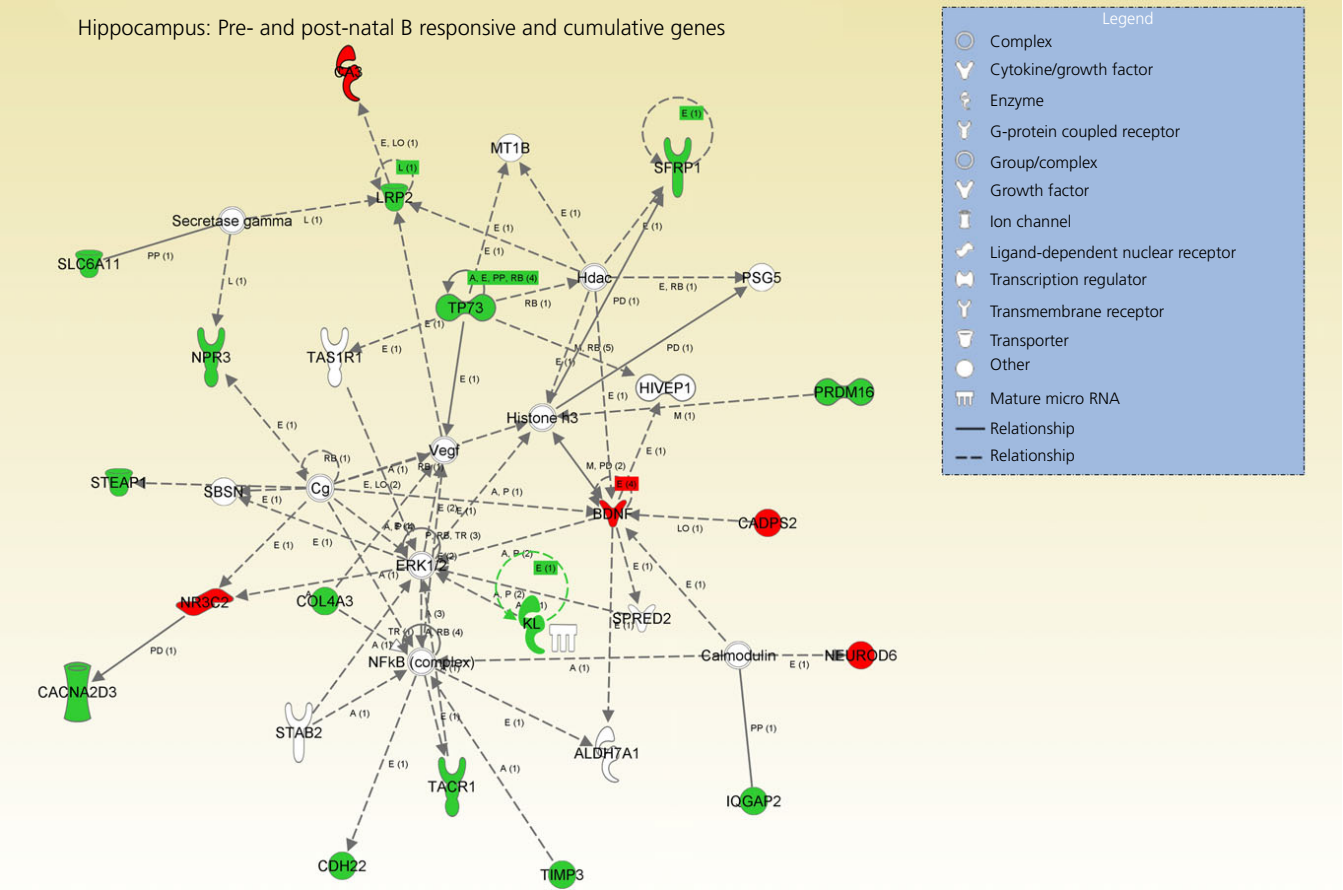
Top significant network generated by ingenuity pathway analysis (IPA) showing the the down‐regulated genes (green) and up‐regulated genes (red) in the hippocampus (score: 39) in response to the overall and cumulative effects of pre‐ and post‐natal exposure to B regardless of timing (gene lists obtained by vector analysis, behavioural categories I and II combined together). The network is displayed with nodes (i.e. genes) and edges (i.e. biological interactions among nodes); in white, the not user‐specific genes added into the network as a result of interactions with the submitted (i.e. user‐specific) genes. Solid lines connecting the genes indicate direct interactions between the nodes and dashed lines implied indirect interactions.

Of the 77 genes submitted to IPA, 62 successfully mapped, and 53 of these were considered as nonredundant ‘focus’ genes with records in the IPA database (see Supporting information, Table S6a). The top 10 significant and enriched biological functions associated with the long‐term and cumulative effects of early‐life B exposure (regardless of timing) are shown in Fig. 5. ‘Cancer’ was the most represented function, with 57 genes, including BDNF, NR2C2 and CADPS2 (1.99 × 10^–8^ < P* *<* *1.23 × 10^–2^). Individual genes such as BDNF, NR3C2 and CADPS2 were also found in multiple significant functions, including in the category ‘Behaviour’ because these genes are well known modulators of spatial memory, cognition, learning (2.73 × 10^–4^ < P* *<* *1.38 × 10^–2^) 15. The network analysis revealed the existence of 3 significant networks with scores between 39 and 17, which together contained 41 out of 53 focus genes (Table 3). The top network (Fig. 6), which was associated with ‘Psychological Disorders, Cardiovascular System, Development and Function, Hematological System Development and Function’, contained 19 ‘focus’ genes and included NR3C2, CAPPS2 and BDNF.

**Table 3:** The Significant Networks Identified in the Ingenuity Pathway Analysis Using the Gene Lists Belonging to the Behavioural Category I and II in the Hippocampus

Network	Score	Focus genes	Genes
1	39	19	ALDH7A1, **BDNF, CA3, CACNA2D3, CADPS2**, Calmodulin, **CDH22,** Cg, **COL4A3**, ERK1/2, Hdac,Histone h3, HIVEP1, **IQGAP2**,** KL, LRP2**, MT1B, **NEUROD6**, NFkB (complex), **NPR3, NR3C2, PRDM16**, PSG5, SBSN, Secretase gamma, **SFRP1, SLC6A11**, SPRED2, STAB2, **STEAP1, TACR1**, TAS1R1, **TIMP3, TP73**, Vegf
2	22	12	**ALK,** APH1B, BASP1, CCS, **CDH6**, CDH18, CDKN1B, **CEMIP**, CKB, COL8A1, DAZ2, DUSP9, EIF5A, ESR1, **FOLR1**, GAS5, HIF1A, INHA, **KCNJ13, MEIS2**, mir‐140, **MYO7A**, OTX2, **PDZD2**, PES1, PRSS23, PSEN1, RDH5, SLC38A2, **SLC6A20, SLC01C1**, SOX2, TOP2B, **TYRP1, ZFHX3**
3	17	10	ACSL5, ACY1, **AGRN**, ALDH9A1, AQP8, **ATP7B, COL14A1**, CSAD, DBI, DCLRE1A, FASN, **HKDC1**, HNF4A, HRASLS, HSD17B13JFI30, **IGDCC4**, IRF1, ITIH3, **KAL1**, mir‐28, PDP1, PPARD, **RHPN2**, S100A2, SHISA5, **SLC13A1**, SMARCA4, SREBF1, TDO2, TP53, TRIM24, USP22, **VIPR1, WFIKKN2**

‘Focus’ genes are highlighted in bold (for the complete list, see the Supporting information, Table S6a).

#### Hypothalamus

**Figure 7:**
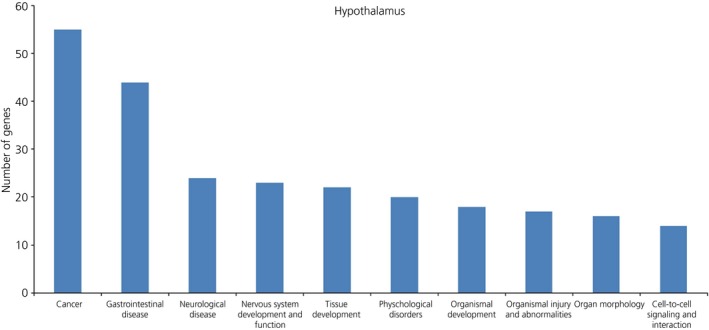
Functional gene ontology for differentially expressed hypothalamic genes in response to post‐natal exposure to B (vector analysis behavioural category IV). The top 10 functional groups with larger number of genes were categorised using Ingenuity Pathway Analysis software (IPA; http://www.ingenuity.com/products/ipa)

**Figure 8:**
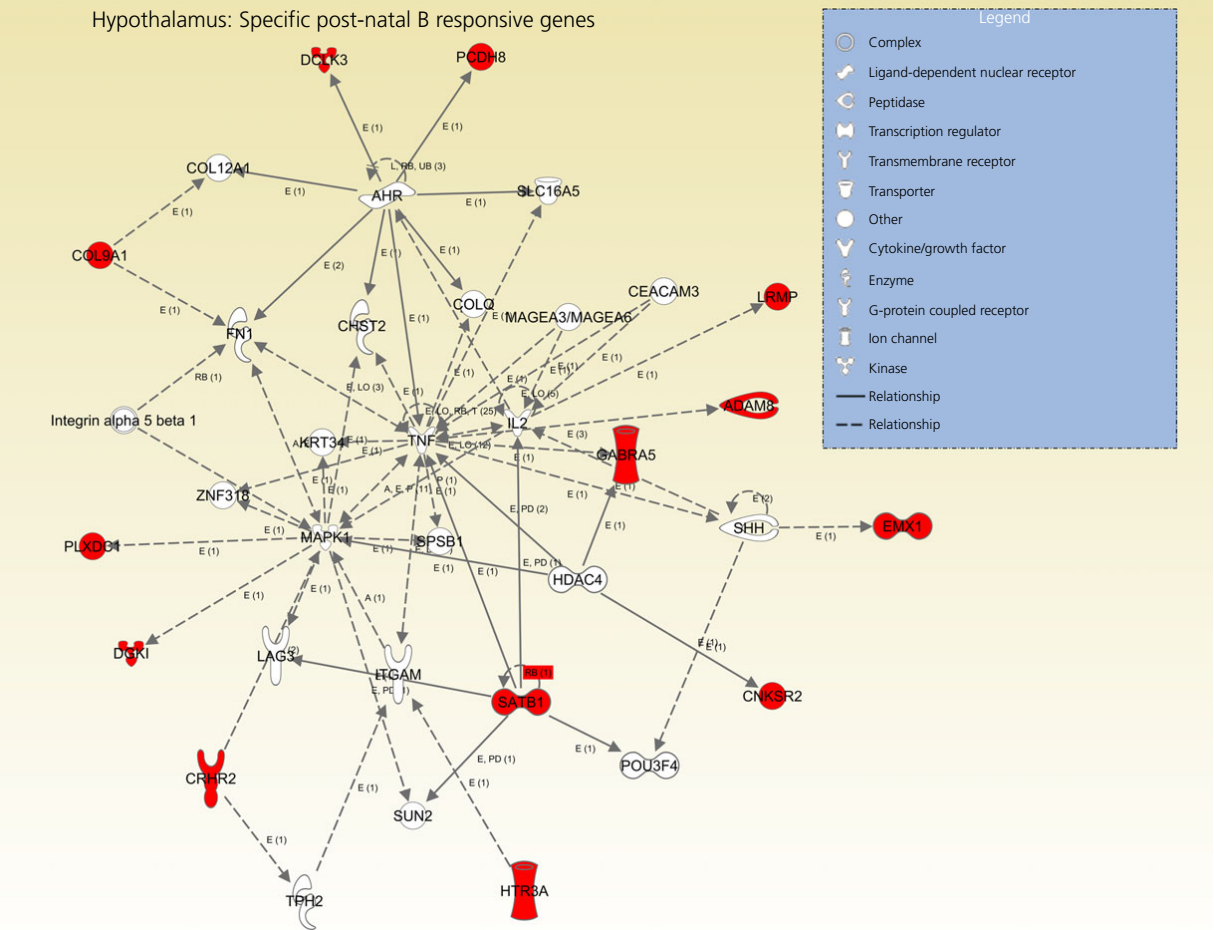
The second most significant network generated by ingenuity pathway analysis (IPA) showing the up‐regulated genes (red) in the hypothalamus (score: 24) that were specifically altered by post‐natal exposure to B (gene list obtained by vector analysis behavioural filtering, category IV). The network is displayed with nodes (i.e. genes) and edges (i.e. biological interactions among nodes); in white, the not user‐specific genes as a result of interactions with the submitted (i.e. user specific) genes. Solid lines connecting the genes indicate direct interactions between the nodes and dashed lines implied indirect interactions.

Out of 71 genes submitted in IPA, 60 successfully mapped, and 47 of these were considered as nonredundant ‘focus’ genes (see Supporting information, Table S6b). The top 10 significant and enriched biological functions specifically and uniquely associated with the long‐term effects of post‐natal B exposure are shown in Fig. 7. Similar to the hippocampus, ‘Cancer’ was the most represented function, with 55 genes (1.66 × 10^–5^ < P* *<* *4.26 × 10^–2^). IPA identified four significant networks with scores ranging from 26 to 15, which together contained 47 out of 49 focus genes (Table 4). As shown in Fig. 8, the second most significant network (score: 24 and 13 ‘focus’ genes) included the gene encoding the corticotrophin‐releasing hormone receptor 2 (CRHR2) and the serotonin receptor HTR3A and was associated with ‘Cellular Development, Cellular Growth and Proliferation, Hematological System Development and Function’.

**Table 4:** The Significant Networks Identified in the Ingenuity Pathway Analysis Using the Gene Lists Belonging to the Behavioural Category IV Hypothalamus

Network	Score	Focus genes	Genes
1	26	14	**AD0RA1**, Akr1c19, **ALDH1A2**, AMOTL2, **ANTXR1**, AR, CDKN1B, CHST12, CISD1, **COL4A2**, CTNNB1, **DACH2**, DPEP1, DUSP9, **FILIP1L**, FSH, IL5, **KCNJ4**, KCTD11, **LMO7**,** LRRK1**, MAGEA11, mir‐224, **MRAS**, MSMB, MT1L, PCCA, **RASL11B**,** RGS12**,** SEMA5A**, SPCS2, TGFB1, TMEM97, TP53, **TYRO3**
2	24	13	**ADAM8**, AHR, CEACAM3, CHST2, **CNKSR2**, COL12A1, **COL9A1**, COLQ, **CRHR2, DCLK3**,** DGKI, EMX1**, FN1, **GABRA5**, HDAC4, **HTR3A**, IL2, lntegrin alpha 5 beta1, ITGAM, KRT34, LAG3, **LRMP**, MAGEA3/MAGEA6, MAPK1, **PCDH8**,** PLXDC1**_,_ POU3F4_,_ **SATB1**, SHH, SLC16A5, SPSB1, SUN2, TNF, TPH2, ZNF318
3	19	11	AGAP2, Akt, ARHGEF2, **ARHGEF6, ARPP21**, ATP1B2, BAIAP2, CACNA1B, CACNA2D1, **CRTAC1**, EPB41L3, ERG, **GRIN2A**, GRM2, GRM3, HTT, KCTD12, **KCTD16, MFSD4**, MPEG1, **NGEF**, PACSIN2, **PIK3R6**, PREX2, PTPN4, PTPN5, **RASD2**, RPH3A, RSU1, SCGB3A1, **SPTBN5, ST8SIA6**, TRAF2, TRIM3, Vegf
4	15	9	ABCC2, ABCG5, ABCG8, BHLHE22, **BHLHE40**,** CDHR2**, Cg, CLOCK, CUL5, **CYP27A1**,** EPHA3**, FHL1, GIP, HES6, HNF4A, HSD11B2, ID1, INS, **KCNS1**, miR‐30c‐5p (and other miRNAs w/seed GUAAACA), **NEUROD6**, OXT, PERP, RNA polymerase II, **SATB2**, SDHC, SLC20A1, SLC25A1, SLC27A2, SORBS1, Sprr2f, SRR, **SSTR5**, TENM4, **ZBTB18**

‘Focus’ genes are highlighted in bold (for the complete list, see the Supporting information, Table S6b).

### qPCR

The three selected genes for qPCR (TTR, SOD3 and GNG11) showed concordant expression values (see Supporting information, Fig S5) and treatment pairwise directional fold changes with RNA‐seq, with a few discrepancies for expression differences of low‐intensity fold changes < 2 (see Supporting information, Fig S6).

## Discussion

The findings reported in the present study indicate that Japanese quail exogenously exposed to the stress hormone corticosterone during pre‐ and/or post‐natal stages of development showed distinct modifications in global gene expression patterns depending on the brain region (hippocampus and hypothalamus) and, most importantly, on the developmental stage at which the stressful treatment occurred. At least to some extent, the key results from the present study are in accordance with our predictions because we found two main treatment‐dependent tissue‐specific transcriptome profiles with (i) genes that responded in a similar way to both pre‐ and post‐natal corticosterone, given that they were commonly differentially expressed across all the stress‐exposed individuals compared to the control birds, or with (ii) genes that responded specifically and uniquely to one of the corticosterone treatment in the pre‐ or post‐natal corticosterone‐exposed birds compared to the pre‐ or post‐natal control birds. As discussed below in detail, our final functional analyses revealed that part of such long‐term treatment‐dependent changes in the brain transcriptome involved functional networks of genes containing conserved regulatory targets of the vertebrate stress system, and including known markers of the programming actions of early‐life adversity. Hence, our experimental data broadly support the hypothesis that overexposure to glucocorticoids during early life is highly likely to be a key driver and a co‐evolved phenomenon underlying the long‐term phenotypic ‘memory’ or ‘storage’ caused by a range of early‐life adverse events 4. Importantly, the present study represents the first attempt to experimentally quantify transcriptome responses resulting from both pre‐ and/or post‐natal stress exposure in a nongenomic model species using RNA‐sequencing. We are aware of limitations of the present study because the analysis was limited to pooled RNA samples and we included both sexes within each biological pool. We have attempted to minimise these limitations by using a very conservative approach throughout the statistical analysis work‐flow that allowed us to control for biological variability and to identify and focus the final functional analyses only on the most consistent responses of long‐term genes to the early‐life protocols on the basis of the specific biological questions addressed in the present study. Furthermore, our qPCR data on three selected genes in the hippocampal pooled samples showed highly similar expression signals and fold changes across the treatment groups, as demonstrated by RNA‐seq. These patterns, although based on only three genes, are indicative of good technical reproducibility of the RNA‐seq data. Nevertheless, the effects observed in the present study on certain target genes would need to be confirmed and biologically further validated in future experiments.

### Long‐term transcriptome‐dependent changes associated with exposure to corticosterone during development regardless of timing

Within the hippocampus, we found a relatively large number of commonly differentially expressed genes (i.e. 53 genes) among all the corticosterone‐exposed birds compared to the controls (i.e. pre‐ and post‐natal corticosterone sensitive genes, behavioural category I) and, of particular interest, there was a significant up‐regulation of the genes encoding the genes MR and BDNF across all the corticosterone‐exposed birds compared to the controls (Fig. 6). Furthermore, we also detected pre‐ and post‐natal corticosterone sensitive genes (i.e. 24 genes) that were modified by the cumulative effects of both pre‐ and post‐natal stressful treatments (behavioural category II). As a result of the biological similarities between these two behavioural categories, we grouped the gene lists together to perform the functional analysis and explored the underlying functional links. Importantly, both MR and BDNF clustered together in the most significant functional network (Fig. 6) via the extracellular signal‐regulated kinase 1/2 (ERK1/2) pathway, which is known to mediate cell proliferation and apoptosis 40. The ERK1/2 pathway is also a redox‐sensitive site 40, suggesting that the oxidative balance in the hippocampus was possibly altered in the treated birds. Indeed, corticosterone‐exposed birds showed down‐regulated expression of TYRP1 (tyrosinase‐related protein 1) (Table 3), a gene implicated in antioxidant defences with respect to detoxifying hydrogen peroxide into water. Intriguingly, we have previously shown that the activity of the antioxidant glutathione peroxidase in the red blood cells was up to 50% higher in the pre‐ and post‐natal corticosterone treated birds relatively to the controls at adulthood, and a marginal nonsignificant treatment increase in the activity of this enzyme (approximately 25%) was also detected in one of the brain tissue examined: the cerebellum 11. Effects such as these on the antioxidant machinery might have avoided rises in terminal damage products given that the levels of oxidative damage to proteins (i.e. protein carbonyls) did not differ across the treatment groups in both of the brain tissues examined (cerebellum and midbrain) and in the erythrocytes 11. However, linking the data from the present study to the latter work is not straightforward because oxidative balance varies among differing tissues, and there may not be any linear relationships between the levels of expression of antioxidant genes and actual antioxidant enzymatic activity. Nevertheless, it appears to be counterintuitive to speculate that the exposure to glucocorticoids during development can result in subsequent alterations in oxidative stress‐dependent pathways within post‐mitotic neuronal tissues. Whether or not such long‐term adjustments could have consequences at the organismal level, affecting the probability of illness, longevity trajectories or reproductive performance, remains an open question. The over‐expression of BDNF in the corticosterone‐treated birds relative to the controls was directly associated with the over‐expression of CADPS2, the gene encoding Ca^2+^ dependent activator protein for secretion 2. This was another interesting result given that, in mice, the latter protein is co‐localised with BDNF in hippocampal neurones and promotes BDNF secretion in this brain region 41. Our data suggest that such a mechanism might be conserved in birds. In rats, hippocampal BDNF mRNA is altered by both pre‐natal stress protocols, including maternal restraint, and post‐natal stressful manipulations, such as maternal separation 42, 43. Reproducing chickens subjected to an unpredictable food regime showed up‐regulated BDNF mRNA in the brain regions analysed (i.e. hypothalamus and pituitary pooled together) compared to controls, and the same directional change was also found in the offspring of the treated birds 23. Overall, our results for the hippocampal BDNF corroborate previous work and support the hypothesis that this neurotrophic factor may be a very important marker of early‐life stress in birds, as it is in mammals. In mammals, decreased BDNF levels are assumed to impair hippocampus‐related learning 44 and can alter anxiety‐like behaviours 45. A study in the zebra finch showed that reduced mRNA expression of MR in the hippocampus can be linked with impaired spatial cognition in birds selected to respond to an acute stressor with high plasma corticosterone levels 46. Hence, it is plausible that the combined over‐expression of both hippocampal BDNF and MR mRNA levels in the early‐life corticosterone‐exposed quail might have triggered adaptive coping strategies to prevent potential hippocampal learning impairments or increased anxiety‐like behaviours. Future studies combining gene expression measurements with behavioural tests would be needed to test this possibility.

In the hypothalamus only, the number of genes incorporated into the behavioural category I and II was limited (13 and 3 genes, respectively) and, hence, commonly up‐ or down‐regulated across all the corticosterone‐exposed birds relative to the controls. These results may be indicative of a limited power in the analyses as a result of the small sample size, or might actually reflect a true biological effect suggesting that, in the Japanese quail, transcriptome changes in the hypothalamus, in contrast to that observed in the hippocampus, are strictly dependent on the developmental stage in which the stressor is experienced.

It should be noted that, although the gene MR in the hippocampus was affected by the early‐life stress protocols, no effect of the developmental environment was detected for the gene encoding GR in either of the brain regions. GR, together with MR, has a key role in mediating the vertebrate stress response 14. This was somewhat unexpected in light of our previous study showing that developmental exposure to pre‐ and post‐natal corticosterone altered acute stress responses in adulthood in our full experimental population, with the pre‐natally exposed birds showing heightened stress responses, whereas such an effect was not observed in the birds exposed to both pre‐ and post‐natal corticosterone 10. Such apparent discrepancies may be explained because, in the present study, the birds were tested only under baseline nonstressful conditions. Under such conditions, only MR remains tonically activated by basal glucocorticoid levels and it is especially active in the hippocampus 14. This possible explanation is supported by a previous study in the chicken showing that the long‐term effects of early‐life adversity (i.e. intermittent social isolation) on the brain transcriptome differed depending on whether the animals were tested at baseline or after restraint stress 25. Future studies should take these environmental‐context dependencies into account when attempting to interpret physiological, behavioural and transcriptome measurements.

### Long‐term transcriptome‐dependent changes associated with either pre‐natal or post‐natal exposure to corticosterone

Relatively fewer genes (i.e. 26) compared to those incorporated in the previous behavioural categories were specifically and uniquely regulated by pre‐natal corticosterone exposure in the hippocampus to perform a functional analysis (i.e. pre‐natal corticosterone sensitive genes, behavioural category III). However, it is worth noting the down‐regulation across the pre‐natally treated quail (in comparison with the pre‐natal controls) of the gene transthyretin (TTR), the carrier of thyroid hormones and retinal binding protein in the cerebrospinal fluid. Such a pre‐natal specific treatment effect might be very important given that the expression of this gene in the rat brain is controlled by MR and GR receptors 47 and can be altered by early‐life experience, such as maternal separation 48. This result also suggests that pre‐natal exposure to corticosterone can alter multiple hormonal signalling pathways linked with the HPA axis. Our pre‐natal protocol was designed to mimic higher embryonic exposure to glucocorticoids of maternal origin, given that chronically stressed mothers deposit higher levels of corticosterone in the yolk compared to control mothers (e.g. 26). However, we do not know the extent to which this presumed long‐term maternal effect on the offspring brain transcriptome should be uniquely attributable to passive and indirect maternal coding signals or is the result of interacting responses of the embryos' own physiology to maternal stress. We need more studies clarifying the ontogeny of the stress physiology in both precocial and altricial species and specifically addressing potential pre‐natal sensitive windows and hence the capacity of the embryo to actively respond to maternal signalling as it develops in the egg.

Although no genes were specifically and uniquely changed by exposure to post‐natal corticosterone upon adulthood in the hippocampus (post‐natal corticosterone sensitive genes, behavioural category IV), several genes (i.e. 71) in the hypothalamus were incorporated in this category. The functional network analysis suggested a central role for the serotonergic system in this context (Fig. 8). These data support the hypothesis of the cross‐talk between serotonin and the HPA axis and also highlight the importance of these two systems in driving the long‐term effects of stress developmental programming 49. A study in mice suggested significant regulatory interactions between serotonin and the corticotrophin‐releasing hormone signalling pathway via the activation of the serotonin receptor HTR2C within the hypothalamus 50. The latter findings support the results from our functional analysis that showed links between the up‐regulated gene coding the serotonin receptor 3A (HTR3A) and the gene coding the corticotrophin‐releasing hormone receptor 2 (CRHR2), which was also up‐regulated in response to post‐natal stress; these two genes clustered together in the second top significant network (Fig. 8). In the chicken, *in ovo* exposure of corticosterone caused dose‐dependent expression changes in hypothalamic serotonergic genes (including up‐regulation of the serotonin receptor HTR1A and down‐regulation of the serotonin biosynthetic enzyme tryptophan hydroxylase) and HPA axis genes (including down‐regulation of corticotrophin‐releasing hormone), and these changes were associated with enhanced aggressive behaviour 20. Although aggressive behaviours were not measured in our experimental birds, it is plausible to speculate that the observed hypothalamic transcriptome changes in the post‐natally treated quail may be associated with distinct behavioural strategies.

### Main organismal functions that were altered by pre‐ and post‐natal exposure to corticosterone

The two pathway analyses performed in the present study suggested that exposure to developmental stress de‐regulated and impaired various biological functions that were associated overall with increased risks of neurological diseases and organismal injuries (Figs 5 and 7) and, in both the hippocampus and hypothalamus, cancer was the most representative functional category. Although these data suggest a harmful and presumably ‘maladaptive’ outcome of early‐life stressful circumstances in line with biomedical studies 4, these results may be biased by the large amount of data originating from cancer research. The idea that developmental stressful conditions may actually be beneficial and confer Darwinian fitness advantages, at least during certain age periods and depending on the environmental context, is based on recent experimental studies in the field of behavioural ecology and eco‐physiology 1, 5, 6. The integration of transcriptome metrics in studying the ‘ecology of stress’ should heavily contribute to opening different scenarios when attempting to investigate the extent to which developmental stress programming may maximise an individual's fitness outcomes and also to furthering our understanding of the integrative role in adaptation of the stress system.

## Conclusions

In summary, we have shown that exposure of the Japanese quail to exogenous corticosterone during pre‐ and post‐natal stages of development caused long‐term changes in the brain transcriptome signature within the hippocampus and hypothalamus, which are two key regulatory regions of the HPA axis. Interestingly, the gene expression responses observed as a consequence of early‐life corticosterone exposure were functionally inter‐related and included known markers of the programming actions of early‐life adversity. These included brain‐derived neurotrophic factor and mineralocorticoid receptor within the hippocampus, as well as corticotrophin‐releasing hormone and serotonin receptors in the hypothalamus. These results indicate important overlaps with the previous literature on early‐life adversity. Our data suggest that increases in glucocorticoid stress hormones may be a fundamental mechanism through which the long‐term phenotypic effects of early‐life stressful conditions emerge and can potentially persist throughout an individual's lifespan. We also showed that the effects of exposure to corticosterone on the brain transcriptome were developmental‐stage dependent. Within the hippocampus, the largest number of differentially expressed genes were the genes similarly altered across all of the stress‐exposed individuals compared to the controls, suggesting that this brain region is similarly sensitive to both pre‐ and post‐natal stress. By contrast, within the hypothalamus, transcriptome profiling was mostly influenced by post‐natal stress alone. These data strongly emphasise the need for more work across different vertebrate species with respect to developmental timings and potential sensitive windows. Future work in this area would also contribute to understanding the potential adaptive role of long‐term transcriptional adjustments in response to early‐life stress and the extent to which they relate to phenotypic differences at the individual level, especially those affecting key organismal fitness traits such as reproduction and longevity outputs. We also need more work aiming to understand whether early‐life environmentally‐driven phenotypic variation could be transferred to the next generations and, in this context, the integration of transcriptome measurements with targeted epigenetic analyses will be of key importance

## Supporting information


**Table S1** TopHat arguments that were deviated from the default settings in the final alignment of the RNA‐seq quail reads to the chicken reference genome.
**Table S2** The assignment of a particular gene to one of the four behavioural categories was established by filtering the data according to the assignment of specific vector analysis classes within each vector analysis performed (as shown in Fig. 1 and Fig. 2 in the Materials and methods).
**Table S3** Alignment basic statistics across the three biological replicates in each treatment [pre‐natal and post‐natal control birds (CC), pre‐natal B‐treated (single injection of corticosterone) and post‐natal control birds (BC), pre‐natal control and post‐natal B‐treated birds (CB), pre‐natal B treated and post‐natal B treated birds (BB)] in the (a) hippocampus and (b) hypothalamus.
**Table S4** Top 20 up‐ or down‐regulated significant transcripts (false discovery rate ≤ 0.10; highlighted below in red and green, respectively) across the pairwise contrasts in the (a) hippocampus and (b) hypothalamus.
**Table S5** List of down‐ and up‐regulated genes (highlighted in green and red, respectively; Ensembl IDs are in ascending order) that met the behavioural filtering categories in the (a) hippocampus and (b) hypothalamus.
**Table S6** Lists of down‐ and up‐regulated genes (highlighted in green and red, respectively) in the (a) hippocampus and (b) hypothalamus submitted to ingenuity pathway analysis (IPA) after filtering the vector analysis data according to the behavioural categories.
**Fig S1** Average Illumina quality Phred scores and their corresponding standard deviations (i.e. ‘SD’ in the legend, estimate of the error score) in each cycle per base‐call along the 76 base reads in the (a) hippocampus (n = 12) and (b) hypothalamus (n = 12) across the three pooled biological replicates (repl 1, repl 2 and repl 3) in each experimental treatment group [pre‐natal and post‐natal control birds (CC), pre‐natal B‐treated (single injection of corticosterone) and post‐natal control birds (BC), pre‐natal control and post‐natal B‐treated birds (CB), pre‐natal B treated and post‐natal B treated birds (BB)].
**Fig S2** Principal component analysis (PCA) plot of all RNA‐seq sample (n = 24) using the RNA‐seq normalised counts [log2 (normalised count + 32)].
**Fig S3** Principal component analysis (PCA) plots of RNA‐seq samples using normalised counts [log2 (normalised count + 32)] in the (a) hippocampal samples (n = 12) and (b) hypothalamic samples (n = 12).
**Fig S4** Graphical representation of vector analyses (full details on the two analyses in Fig. 1; see also Materials and methods) in the (a) hippocampus and (b) hypothalamus before partitioning and filtering the data according to specific gene behavioural categories (for full details, Materials and methods).
**Fig S5** Expression values for the genes transthyretin (TTR); superoxide dismutase extracellular 3 (SOD3); and guanine nucleotide binding protein (G‐protein), Gamma 11 (GNG11) from qPCR and RNA‐seq.
**Fig S6** Fold changes (FC) for the genes transthyretin (TTR); superoxide dismutase extracellular 3 (SOD3); and guanine nucleotide binding protein (G‐protein), Gamma 11 (GNG11) derived on the basis of samples processed using RNA‐seq and the quantitative polymerase chain reaction (PCR) across the six pairwise comparisons. Plotted values represent expression averages from the three biological replicates.
