# Supplementary material for: Pre‐ and Post‐Natal Stress Programming: Developmental Exposure to Glucocorticoids Causes Long‐Term Brain‐Region Specific Changes to Transcriptome in the Precocial Japanese Quail
Source: J Neuroendocrinol. 2016 May 25;28(5):10.1111/jne.12387. doi: 10.1111/jne.12387 (PMC5103168; doi:10.1111/jne.12387)
Supplement: Supplementary file 1 — Table S1 TopHat arguments that were deviated from the default settings in the final alignment of the RNA‐seq quail reads to the chicken reference genome. Table S2 The assignment of a particular gene to one of the four behavioural categories was established by filtering the data according to the assignment of specific vector analysis classes within each vector analysis performed (as shown in Fig. 1 and Fig. 2 in the Materials and methods). Table S3 Alignment basic statistics across the three biological replicates in each treatment [pre‐natal and post‐natal control birds (CC), pre‐natal B‐treated (single injection of corticosterone) and post‐natal control birds (BC), pre‐natal control and post‐natal B‐treated birds (CB), pre‐natal B treated and post‐natal B treated birds (BB)] in the (a) hippocampus and (b) hypothalamus. Table S4 Top 20 up‐ or down‐regulated significant transcripts (false discovery rate ≤ 0.10; highlighted below in red and green, respectively) across the pairwise contrasts in the (a) hippocampus and (b) hypothalamus. Table S5 List of down‐ and up‐regulated genes (highlighted in green and red, respectively; Ensembl IDs are in ascending order) that met the behavioural filtering categories in the (a) hippocampus and (b) hypothalamus. Table S6 Lists of down‐ and up‐regulated genes (highlighted in green and red, respectively) in the (a) hippocampus and (b) hypothalamus submitted to ingenuity pathway analysis (IPA) after filtering the vector analysis data according to the behavioural categories. Fig S1 Average Illumina quality Phred scores and their corresponding standard deviations (i.e. ‘SD’ in the legend, estimate of the error score) in each cycle per base‐call along the 76 base reads in the (a) hippocampus (n = 12) and (b) hypothalamus (n = 12) across the three pooled biological replicates (repl 1, repl 2 and repl 3) in each experimental treatment group [pre‐natal and post‐natal control birds (CC), pre‐natal B‐treated (single injection of corticosterone [file JNE-28-0-s001.docx]

**Supplementary**

**Pre- and post-natal stress programming: developmental exposure to glucocorticoids causes long-term brain-region specific changes to transcriptome in the precocial Japanese quail**

**1. Supplementary Materials and Methods**

***1.1 cDNA library preparation and high-throughput RNA-seq***

The RNA pooled samples were processed for RNA-seq using standard TruSeq™ RNA Sample Preparation kit (Illumina, Little Chesterford, Essex, UK). Briefly, the poly-A containing mRNA molecules were purified using poly-T oligo-attached magnetic beads. The mRNA was fragmented and the fragments were then synthesised into first strand c-DNA using reverse transcriptase. Then, second strand cDNA was obtained using DNA polymerase I and RNase H. In the next steps, cDNA fragments were processed in order to allow the ligation of the adapters at the fragment ends, generating flow-cell-suitable templates. The products were then purified and enriched by PCR to obtain the final cDNA libraries. Fragment distribution among the libraries was assessed using the Agilent 2100 Bioanalyzer DNA 100 chip. As indicated by the Manufacturer, fragment size averaged 309.16 ± 2.25 bases (mean ± s.e.m).

Sequencing was performed on a Genome Analyzer IIX (GAIIX) platform at the Glasgow Polyomics Facility (University of Glasgow, UK) as follow. The cDNA libraries were first hybridised onto the flow cell covered with complementary surface-bound primers. Each sample was loaded in one lane, resulting in a total of 24 lanes in 6 different flow cells; samples from different treatments were randomised across sequencing flow cells and lanes within flow cells. Hybridised cDNA templates were then extended via isothermal bridging amplification in order to create an ultra-high density sequencing flow-cell with approximately 30.000.000 clusters per lane. The fragments in each cluster were sequenced using a four-color sequencing-by-synthesis technology that employs reversible terminators with removable florescent dyes (Sequencing reagents version 5, Illumina, Little Chesterford, Essex, UK) and the signals emitted recorded. The sequencing run terminated after 76 cycles and yielded reads (i.e. sequences of nucleotides) with a maximum length of 76 bases.

***1.2 qPCR assays***

First-stranded cDNA was synthesised from total RNA (approximately 250ng) from each sample pool in a reaction mixture (50µl) containing Moloney-Murine Leukaemia Virus (M-MLV) Reverse Transcriptase (200units/µl; Invitrogen, Life Technologies, Paisley, UK); random hexamers (50µM; Promega, Southampton, UK); dNTPs (10mM); Rnasin (40units/µl; Promega), dithiothreitol (DTT; 0.1M), and the appropriate volume of free DNAse/RNAse free water (1). The reaction was first incubated at 65ºC for 5min, then at 37ºC for 50min and finally at 70ºC for 15min. The cDNA formed was used as a template for qPCR for the genes of interest and the housekeeping geneβ-actin. β-actin was regarded as the best reference gene (M = 0.30; M ≥ 0.34 for all the others) for our samples using a chicken (*Gallus gallus*) GeNorm kit (Primerdesign, Southampton, UK). Specific PerfectProbe™ primers (Primerdesign) were designed based on the reference chicken genome and validated using a pool of quail cDNA from our experimental birds by Primer Design. These primers amplified single products with no dimer pairs. TTR sense primer: ATGAATATGCTGATGTGGTGTTC, anti-sense primer: GCAGTTGTTGAGTAAGAGAAAGG (GenBank accession no. NM_205335). SOD3 sense primer: CCAACCTCTTCGCCACAAT, anti-sense primer: CAGCATTTCCATTTTCCAGACT (GenBank accession no. XM_420760). GNG11 sense primer: GATGATCTGAGCGAGAAGGAC, anti-sense primer: TCGGAGCACTTGGACACC (GenBank accession no. XM_00123433). All qPCR reactions were run in duplicate. Each qPCR reaction mixture (20μl) contained 10μl of 2x Brilliant III Ultra-Fast QPCR Master Mix (Agilent technologies), 1μl of specific PerfectProbe™ primer (Primerdesign) at a working concentration of 300nM, 0.3μl of reference dye, 5µl of cDNA template (concentration: 1ng/µl), and 3.7μl of RNAse/DNAase-free water with no-template controls and blanks. Reactions were carried out on a Stratagene MX 3005P (Agilent Technologies) at 95°C for 3 min, then 50 cycles of 95°C for 15s, 60°C for 20s. We used the Delta Ct method (ΔCt) to quantify the relative expression of the gene of interest relative to β-actin (2).

***1.3 Raw data quality control***

The raw sequencing data were generated using Casava version 1.7.0 and stored in 24 files in fastQ format (3). Initial standard investigations using FastQC software (version .10.0) confirmed the presence of adapter sequences in small proportion (<0.5%) of 3’end of reads due to the corresponding cDNA fragments being shorter than 76 bases. To avoid problems with downstream analyses, we used an “*in-house*” software routinely employed in the Glasgow Polyomics Facility to remove these contaminated reads.

***1.4 Trimming and mapping of the RNA-seq reads***

Preliminary alignment of quail reads to the chicken genome using the Bowtie aligning software (4) showed that only a very small percentage of reads (< 40%) aligned to the chicken reference genome when up to two mismatches were allowed. Consequently, the 76 bases long reads were shortened to 36 bases. The trimming of the reads also assured high quality reads (Q scores range: 38-34) with a flatter error profile along the read (Fig. S1), and provided a reasonable compromise between the high number of aligned reads and the small number of reads mapped to more than one location in the genome.

**Reference list**

1. O'Shaughnessy PJ, Murphy L. Cytochrome P-450 17 alpha-hydroxylase protein and mRNA in the testis of the testicular feminized (Tfm) mouse. *Journal of Molecular Endocrinology* 1993; 1:77-82.
2. Schmittgen TD, Livak KJ. Analyzing real-time PCR data by the comparative CT method. *Nature Protocols* 2008; 3:1101-1108.
3. Cock PJA, Fields CJ, Goto N, Heuer ML, Rice PM. The Sanger FASTQ file format for sequences with quality scores, and the Solexa/Illumina FASTQ variants. *Nucleic Acids Research* 2010;38:1767-1771.
4. Langmead B, Trapnell C, Pop M, Salzberg SL. Ultrafast and memory-efficient alignment of short DNA sequences to the human genome. *Genome Biology* 2009;10:R25.

**Table S1.** TopHat arguments that were deviated from the default settings in the final alignment of the RNA-seq quail reads to the chicken reference genome. These deviations allowed us to obtain a reasonably high number of quail aligned reads to the chicken reference minimising the change of reads mapped to more than one location in the genome.

| **TopHat parameters** | **Setting used** |
| --- | --- |
| --initial-read-mismatches  (i.e. number of mismatches allowed for each read) | 3 (default 2) |
| --segment-length  (i.e. minimum segment read length) | 18 (default 25) |
| --segment-mismatches  (i.e. number of mismatches allowed in each segment alignment) | 1 (default 2) |
| --min-anchor-length  (i.e. number of bases supporting every junction involved in sliced alignments by at least one read) | 12 (default 8) |

**Table S2.** The assignment of a particular gene to one of the four behavioral categories was established by filtering the data according to the assignment of specific vector analysis classes within each Vector Analysis performed (as shown in Fig 1 and Fig 2 in Material and Methods). Gene responses to B in one or both pre- and post-natal environments were classified as “Specific” or “Same” if Vsum vector heads were found in the corresponding sectors of the Cartesian space (see Fig 1, Material and Methods) and |Vsum| ≥ 40 and *p* ≤ 0.05; a gene response was defined as unchanged when |Vsum| ≤ 40 and *p* ≥ 0.05 regardless of the Vsum vector head position.

**Table S3.** Alignment basic statistics across the 3 biological replicates in each treatment (CC, BC, CB, and BB) in (a) hippocampus and (b) hypothalamus. Data were expressed as % relatively to the total number of sequenced reads. The latter information was extracted from the FastQC summary outputs.


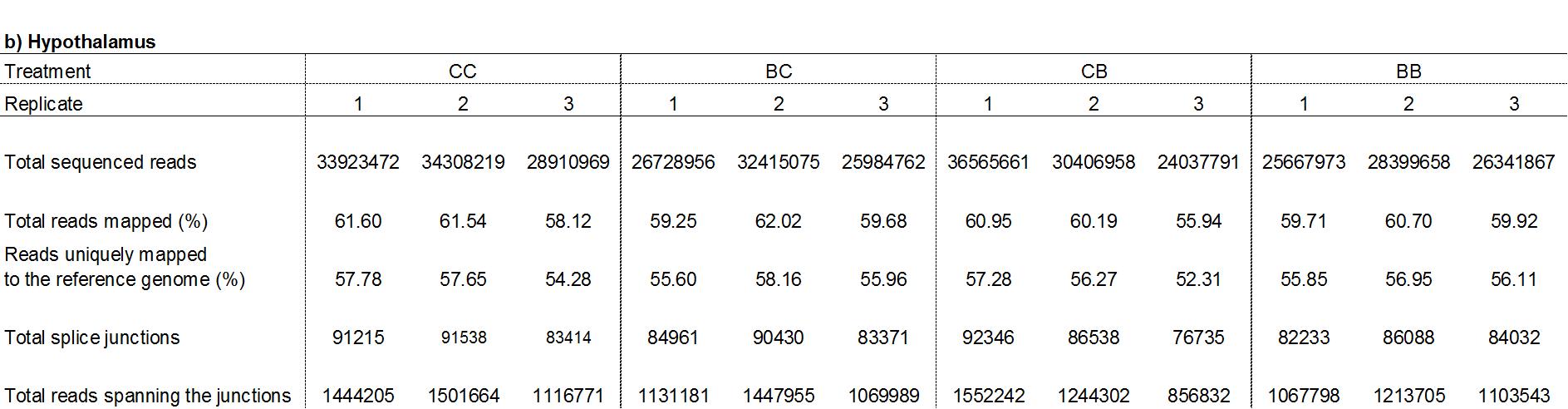

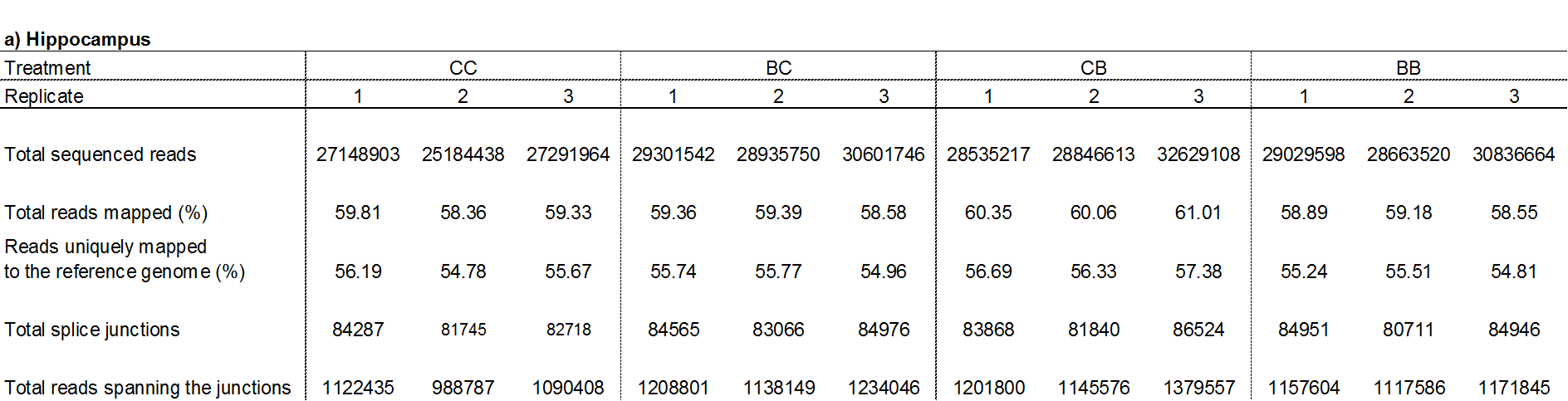


**Table S4.** Top 20 up- or down-regulated significant transcripts (FDR ≤ 0.10; highlighted below in red and green, respectively) across the pair-wise contrasts in the (a) hippocampus and (b) hypothalamus. Genes were sorted according to the RankProducts statistics in ascending order and cut at the level of FDR 0.1. FC denotes the fold change.

|  | 1. **Hippocampus** | | | |  | | | | | | | | | | | | | | |  | | | | |  | | | | | | | |  | | | |
| --- | --- | --- | --- | --- | --- | --- | --- | --- | --- | --- | --- | --- | --- | --- | --- | --- | --- | --- | --- | --- | --- | --- | --- | --- | --- | --- | --- | --- | --- | --- | --- | --- | --- | --- | --- | --- |
|  | **Contrast:**  **BC *vs* CC**  (1^st^ class *vs* 2^nd^ class) | | | | | | | | | | | | | | | | | | | | | | | | | | | | | | | |  | | | |
|  | *(1) Top 20 up-regulated genes under 1*^st^ *class* | | | | | | | | | | | | | | | | | | |  | | | | |  | | | | | | | |  | | | |
|  |  | Ensembl ID | | | | | | | | Description | | | | | | | | | | FDR | | | | | FC | | | | | | | |  | | | |
|  |  | ENSGALG00000006137 | | | | | | | | Rho GTPase activating protein 22 | | | | | | | | | | 0.0020 | | | | | 1.9148 | | | | | | | |  | | | |
|  |  | ENSGALG00000012544 | | | | | | | | UDP-N-acetyl-alpha-D-galactosamine: polypeptide N-cetylgalactosaminyltransferase 5 (GalNAc-T5) | | | | | | | | | | 0.0023 | | | | | 1.9347 | | | | | | | |  | | | |
|  |  | ENSGALG00000008723 | | | | | | | | complement component 1, q subcomponent-like 3 | | | | | | | | | | 0.0025 | | | | | 1.9635 | | | | | | | |  | | | |
|  |  | ENSGALG00000016465 | | | | | | | | similar to egg envelope component ZPAX | | | | | | | | | | 0.0040 | | | | | 1.9502 | | | | | | | |  | | | |
|  |  | ENSGALG00000014414 | | | | | | | | gamma-aminobutyric acid (GABA) receptor, rho 3 | | | | | | | | | | 0.0050 | | | | | 1.9303 | | | | | | | |  | | | |
|  |  | ENSGALG00000012327 | | | | | | | | inhibin, beta A | | | | | | | | | | 0.0051 | | | | | 1.8766 | | | | | | | |  | | | |
|  |  | ENSGALG00000000184 | | | | | | | | solute carrier family 27 (fatty acid transporter), member 6 | | | | | | | | | | 0.0053 | | | | | 1.8676 | | | | | | | |  | | | |
|  |  | ENSGALG00000008135 | | | | | | | | SATB homeobox 2 | | | | | | | | | | 0.0053 | | | | | 1.7825 | | | | | | | |  | | | |
|  |  | ENSGALG00000006676 | | | | | | | | retinaldehyde binding protein 1 | | | | | | | | | | 0.0060 | | | | | 1.9513 | | | | | | | |  | | | |
|  |  | ENSGALG00000016095 | | | | | | | | empty spiracles homeobox 1 | | | | | | | | | | 0.0064 | | | | | 1.7828 | | | | | | | |  | | | |
|  |  | ENSGALG00000016499 | | | | | | | | hypothetical protein LOC770429 | | | | | | | | | | 0.0075 | | | | | 1.7465 | | | | | | | |  | | | |
|  |  | ENSGALG00000000507 | | | | | | | | copine VII | | | | | | | | | | 0.0078 | | | | | 1.7435 | | | | | | | |  | | | |
|  |  | ENSGALG00000005347 | | | | | | | | similar to ADAMTS18 protein | | | | | | | | | | 0.0079 | | | | | 1.7224 | | | | | | | |  | | | |
|  |  | ENSGALG00000005802 | | | | | | | | fms-related tyrosine kinase 4 | | | | | | | | | | 0.0082 | | | | | 1.7570 | | | | | | | |  | | | |
|  |  | ENSGALG00000016500 | | | | | | | | FK506 binding protein 1B, 12.6 kDa | | | | | | | | | | 0.0083 | | | | | 1.7559 | | | | | | | |  | | | |
|  |  | ENSGALG00000005772 | | | | | | | | BCL2-related ovarian killer | | | | | | | | | | 0.0085 | | | | | 1.7838 | | | | | | | |  | | | |
|  |  | ENSGALG00000015720 | | | | | | | | chondrolectin | | | | | | | | | | 0.0088 | | | | | 1.7736 | | | | | | | |  | | | |
|  |  | ENSGALG00000015271 | | | | | | | | filamin A interacting protein 1-like | | | | | | | | | | 0.0090 | | | | | 1.6675 | | | | | | | |  | | | |
|  |  | ENSGALG00000013925 | | | | | | | | v-kit Hardy-Zuckerman 4 feline  sarcoma viral oncogene homolog | | | | | | | | | | 0.0096 | | | | | 1.6287 | | | | | | | |  | | | |
|  |  | ENSGALG00000017064 | | | | | | | | replication factor C (activator 1) 3, 38kDa | | | | | | | | | | 0.0114 | | | | | 1.6222 | | | | | | | |  | | | |
|  |  |  | | | | | | | |  | | | | | | | | | |  | | | | |  | | | | | | | |  | | | |
|  | *(2) Top 20 down-regulated genes under 1*^st^ *class* | | | | | | | | | | | | | | | | | | |  | | | | |  | | | | | | | |  | | | |
|  |  | Ensembl ID | | | | | | Description | | | | | | | | FDR | | | | | | | | | FC | | | | | | | |  | | | |
|  |  | ENSGALG00000001696 | | | | | | S-antigen; retina and pineal gland (arrestin) | | | | | | | | < 0.0001 | | | | | | | | | -18.196 | | | | | | | |  | | | |
|  |  | ENSGALG00000013154 | | | | | | solute carrier organic anion transporter family, member 1C1 | | | | | | | | < 0.0001 | | | | | | | | | -8.0053 | | | | | | | |  | | | |
|  |  | ENSGALG00000015143 | | | | | | transthyretin | | | | | | | | < 0.0001 | | | | | | | | | -5.8174 | | | | | | | |  | | | |
|  |  | ENSGALG00000007875 | | | | | | endothelin converting enzyme-like 1 | | | | | | | | 0.0003 | | | | | | | | | -3.2126 | | | | | | | |  | | | |
|  |  | ENSGALG00000011859 | | | | | | eye-globin | | | | | | | | 0.0012 | | | | | | | | | -3.5947 | | | | | | | |  | | | |
|  |  | ENSGALG00000011369 | | | | | | LIM homeobox 8 | | | | | | | | 0.0013 | | | | | | | | | -3.6024 | | | | | | | |  | | | |
|  |  | ENSGALG00000016553 | | | | | | transmembrane protein 27 | | | | | | | | 0.0015 | | | | | | | | | -3.2546 | | | | | | | |  | | | |
|  |  | ENSGALG00000001490 | | | | | | Potassium inwardly-rectifying channel, subfamily J, member 13 | | | | | | | | 0.0017 | | | | | | | | | -2.8839 | | | | | | | |  | | | |
|  |  | ENSGALG00000018557 | | | | | | similar to extracellular-superoxide dismutase (EC 1.15.1.1); superoxide dismutase 3, extracellular | | | | | | | | 0.0018 | | | | | | | | | -2.8748 | | | | | | | |  | | | |
|  |  | ENSGALG00000012908 | | | | | | solute carrier family 13 (sodium/sulfate symporters), member 4 | | | | | | | | 0.0019 | | | | | | | | | -3.6478 | | | | | | | |  | | | |
|  |  | ENSGALG00000007367 | | | | | | WAP, follistatin/kazal, immunoglobulin,  kunitz and netrin domain containing 2 | | | | | | | | 0.0019 | | | | | | | | | -2.6331 | | | | | | | |  | | | |
|  |  | ENSGALG00000016020 | | | | | | chloride intracellular channel 6 | | | | | | | | 0.0021 | | | | | | | | | -2.9338 | | | | | | | |  | | | |
|  |  | ENSGALG00000017343 | | | | | | folate receptor 1 (adult) | | | | | | | | 0.0027 | | | | | | | | | -2.4622 | | | | | | | |  | | | |
|  |  | ENSGALG00000007179 | | | | | | ATPase type 13A5 | | | | | | | | 0.0028 | | | | | | | | | -2.6189 | | | | | | | |  | | | |
|  |  | ENSGALG00000003034 | | | | | | somatostatin II | | | | | | | | 0.0028 | | | | | | | | | -2.4014 | | | | | | | |  | | | |
|  |  | ENSGALG00000015918 | | | | | | EF-hand calcium binding protein 1 | | | | | | | | 0.0030 | | | | | | | | | -2.3182 | | | | | | | |  | | | |
|  |  | ENSGALG00000015595 | | | | | | G protein-coupled receptor 78 | | | | | | | | 0.0031 | | | | | | | | | -2.3334 | | | | | | | |  | | | |
|  |  | ENSGALG00000000733 | | | | | | myosin VIIA | | | | | | | | 0.0031 | | | | | | | | | -2.3271 | | | | | | | |  | | | |
|  |  | ENSGALG00000003573 | | | | | | hippocalcin | | | | | | | | 0.0033 | | | | | | | | | -2.4069 | | | | | | | |  | | | |
|  |  | ENSGALG00000013168 | | | | | | islet amyloid polypeptide | | | | | | | | 0.0041 | | | | | | | | | -2.2804 | | | | | | | |  | | | |
|  | **Contrast:**  **CB *vs* CC** (1^st^ class *vs* 2^nd^ class) | | | | | | | | | | | | | | | | | | | | | | | | | | | | | | | |  | | | |
|  | *(1) Top 20 up-regulated genes under 1*^st^ *class* | | | | | | | | | | | | | | | | | |  | | | | | | | |  | | | | | |  | | | |
|  |  | Ensembl ID | | | | | | Description | | | | | | | | FDR | | | | | | | | | FC | | | | | | | |  | | | |
|  |  | ENSGALG00000002577 | | | | | | [StAR-related lipid transfer (START) domain containing 10](http://david.abcc.ncifcrf.gov/geneReportFull.jsp?rowids=1211115) | | | | | | | | 0.0300 | | | | | | | | | 1.9365 | | | | | | | |  | | | |
|  |  | ENSGALG00000004322 | | | | | | uncharacterised | | | | | | | | 0.0773 | | | | | | | | | 1.6685 | | | | | | | |  | | | |
|  |  | ENSGALG00000014414 | | | | | | [gamma-aminobutyric acid (GABA) receptor, rho 3](http://david.abcc.ncifcrf.gov/geneReportFull.jsp?rowids=1205517) | | | | | | | | 0.0840 | | | | | | | | | 1.8285 | | | | | | | |  | | | |
|  |  |  | | | | | |  | | | | | | | |  | | | | | | | | |  | | | | | | | |  | | | |
|  | *(2) Top 20 down-regulated genes under 1*^st^ *class* | | | | | | | | | | | | | | |  | | | | | | | | |  | | | | | | | |  | | | |
|  |  | Ensembl ID | | | | | | Description | | | | | | | | FDR | | | | | | | | | FC | | | | | | | |  | | | |
|  |  | ENSGALG00000013154 | | | | | | solute carrier organic anion transporter family, member 1C1 | | | | | | | | 0.0045 | | | | | | | | | -3.3707 | | | | | | | |  | | | |
|  |  | ENSGALG00000012908 | | | | | | solute carrier family 13 (sodium/sulfate symporters), member 4 | | | | | | | | 0.0050 | | | | | | | | | -2.8656 | | | | | | | |  | | | |
|  |  | ENSGALG00000007367 | | | | | | WAP, follistatin/kazal, immunoglobulin,  kunitz and netrin domain containing 2 | | | | | | | | 0.0090 | | | | | | | | | -2.6494 | | | | | | | |  | | | |
|  |  | ENSGALG00000011859 | | | | | | eye-globin | | | | | | | | 0.0205 | | | | | | | | | -2.6732 | | | | | | | |  | | | |
|  |  | ENSGALG00000015143 | | | | | | transthyretin | | | | | | | | 0.0242 | | | | | | | | | -4.1646 | | | | | | | |  | | | |
|  |  | ENSGALG00000001490 | | | | | | uncharacterised | | | | | | | | 0.0427 | | | | | | | | | -2.1829 | | | | | | | |  | | | |
|  |  | ENSGALG00000016553 | | | | | | transmembrane protein 27 | | | | | | | | 0.0441 | | | | | | | | | -2.2201 | | | | | | | |  | | | |
|  |  | ENSGALG00000001696 | | | | | | S-antigen; retina and pineal gland (arrestin) | | | | | | | | 0.0503 | | | | | | | | | -2.9875 | | | | | | | |  | | | |
|  |  | ENSGALG00000011858 | | | | | | potassium voltage-gated channel,  subfamily H (eag-related), member 5 | | | | | | | | 0.0530 | | | | | | | | | -1.9626 | | | | | | | |  | | | |
|  |  | ENSGALG00000010934 | | | | | | InaD-like (Drosophila) | | | | | | | | 0.0650 | | | | | | | | | -1.9627 | | | | | | | |  | | | |
|  |  | ENSGALG00000018557 | | | | | | similar to extracellular-superoxide dismutase (EC 1.15.1.1); superoxide dismutase 3, extracellular | | | | | | | | 0.0673 | | | | | | | | | -2.0535 | | | | | | | |  | | | |
|  |  | ENSGALG00000017343 | | | | | | folate receptor 1 (adult) | | | | | | | | 0.0718 | | | | | | | | | -1.9214 | | | | | | | |  | | | |
|  |  | ENSGALG00000016017 | | | | | | solute carrier family 4, sodium borate transporter, member 11 | | | | | | | | 0.0871 | | | | | | | | | -1.8846 | | | | | | | |  | | | |
|  |  | ENSGALG00000007179 | | | | | | ATPase type 13A5 | | | | | | | | 0.0915 | | | | | | | | | -1.9503 | | | | | | | |  | | | |
|  |  | ENSGALG00000013515 | | | | | | solute carrier family 4, sodium bicarbonate cotransporter, member 5;  similar to sodium bicarbonate cotransporter-like protein | | | | | | | | 0.0940 | | | | | | | | | -1.8976 | | | | | | | |  | | | |
|  |  | ENSGALG00000008874 | | | | | | solute carrier family 13 (sodium/sulfate symporters), member 1 | | | | | | | | 0.0943 | | | | | | | | | -1.9148 | | | | | | | |  | | | |
|  |  | ENSGALG00000016554 | | | | | | angiotensin I converting enzyme (peptidyl-dipeptidase A) 2 | | | | | | | | 0.0952 | | | | | | | | | -1.8784 | | | | | | | |  | | | |
|  |  | ENSGALG00000006838 | | | | | | similar to iron binding protein | | | | | | | | 0.0970 | | | | | | | | | -1.6450 | | | | | | | |  | | | |
|  |  | ENSGALG00000011813 | | | | | | HEG homolog 1 (zebrafish) | | | | | | | | 0.0974 | | | | | | | | | -1.7349 | | | | | | | |  | | | |
|  |  | ENSGALG00000017059 | | | | | | mab-21-like 1 (C. elegans) | | | | | | | | 0.0979 | | | | | | | | | -2.0561 | | | | | | | |  | | | |
|  | **Contrast:**  **BB *vs* CC** (1^st^ class *vs* 2^nd^ class) | | | | | | | | | | | | | | | | | | | | | | | | | | | | | | |  |  | | | |
|  | *(1) Top 20 up-regulated genes under 1*^st^ *class* | | | | | | | | | | | | | | | | | | | | | | | |  | | | | | | |  |  | | | |
|  |  | | | Ensembl ID | | Description | | | | | | | | | | FDR | | | | | | | | | FC | | | | | | |  | | | | |
|  |  | | | ENSGALG00000012226 | | chromosome 7 open reading frame 16 | | | | | | | | | | 0.0473 | | | | | | | | | 3.1520 | | | | | | |  | | | | |
|  |  | | | ENSGALG00000004322 | | uncharacterised | | | | | | | | | | 0.0502 | | | | | | | | | 1.9054 | | | | | | |  | | | | |
|  |  | | | ENSGALG00000015018 | | calsequestrin 2 (cardiac muscle) | | | | | | | | | | 0.0533 | | | | | | | | | 3.1243 | | | | | | |  | | | | |
|  |  | | | ENSGALG00000015857 | | carbonic anhydrase III, muscle specific | | | | | | | | | | 0.0548 | | | | | | | | | 1.9855 | | | | | | |  | | | | |
|  |  | | | ENSGALG00000008193 | | reelin | | | | | | | | | | 0.0615 | | | | | | | | | 2.4139 | | | | | | |  | | | | |
|  |  | | | ENSGALG00000012285 | | BAI1-associated protein 2-like 2 | | | | | | | | | | 0.0620 | | | | | | | | | 3.2025 | | | | | | |  | | | | |
|  |  | | | ENSGALG00000005985 | | growth differentiation factor 10 | | | | | | | | | | 0.0683 | | | | | | | | | 2.1699 | | | | | | |  | | | | |
|  |  | | | ENSGALG00000024278 | | uncharacterised | | | | | | | | | | 0.0725 | | | | | | | | | 2.8673 | | | | | | |  | | | | |
|  |  | | | ENSGALG00000003894 | | cerebellin 1 precursor | | | | | | | | | | 0.0730 | | | | | | | | | 2.9982 | | | | | | |  | | | | |
|  |  | | | ENSGALG00000008908 | | neurogenic differentiation 1 | | | | | | | | | | 0.0756 | | | | | | | | | 2.1326 | | | | | | |  | | | | |
|  |  | | | ENSGALG00000004527 | | hypothetical protein LOC776119  unc-13 homolog C (C. elegans); similar to Munc13-3 | | | | | | | | | | 0.0852 | | | | | | | | | 2.0334 | | | | | | |  | | | | |
|  |  | | | ENSGALG00000023430 | | uncharacterised | | | | | | | | | | 0.0865 | | | | | | | | | 2.4479 | | | | | | |  | | | | |
|  |  | | | ENSGALG00000001695 | | gamma-aminobutyric acid (GABA) A receptor, alpha 6 | | | | | | | | | | 0.0878 | | | | | | | | | 2.6068 | | | | | | |  | | | | |
|  |  | | | ENSGALG00000000920 | | cingulin | | | | | | | | | | 0.0896 | | | | | | | | | 2.5918 | | | | | | |  | | | | |
|  |  | | | ENSGALG00000012544 | | UDP-N-acetyl-alpha-D-galactosamine: polypeptide N-acetylgalactosaminyltransferase 5 (GalNAc-T5) | | | | | | | | | | 0.0962 | | | | | | | | | 1.6314 | | | | | | |  | | | | |
|  |  | | | ENSGALG00000009241 | | secreted frizzled-related protein 2 | | | | | | | | | | 0.0968 | | | | | | | | | 2.0671 | | | | | | |  | | | | |
|  |  | | | ENSGALG00000017417 | | similar to ubiquitin specific proteinase 43 | | | | | | | | | | 0.0984 | | | | | | | | | 2.2327 | | | | | | |  | | | | |
|  |  | | | ENSGALG00000000441 | | potassium voltage-gated channel,  shaker-related subfamily, member 10 | | | | | | | | | | 0.0994 | | | | | | | | | 2.5667 | | | | | | |  | | | | |
|  |  | | | ENSGALG00000005842 | | glycerophosphodiester phosphodiesteras e domain containing 2 | | | | | | | | | | 0.0995 | | | | | | | | | 2.8442 | | | | | | |  | | | | |
|  |  | | |  | |  | | | | | | | | | |  | | | | | | | | |  | | | | | | |  | | | | |
|  | *(2) Top 20 down-regulated genes under 1*^st^ *class* | | | | | | | | | | | | | | |  | | | | | | | | |  | | | | | | |  | | | | |
|  |  | | | Ensembl ID | | Description | | | | | | | | | | FDR | | | | | | | | | FC | | | | | | |  | | | | |
|  |  | | | ENSGALG00000015143 | | transthyretin | | | | | | | | | | < 0.0001 | | | | | | | | | -28.0977 | | | | | | |  | | | | |
|  |  | | | ENSGALG00000013154 | | solute carrier organic anion transporter family, member 1C1 | | | | | | | | | | < 0.0001 | | | | | | | | | -6.5400 | | | | | | |  | | | | |
|  |  | | | ENSGALG00000011859 | | eye-globin | | | | | | | | | | < 0.0001 | | | | | | | | | -4.5587 | | | | | | |  | | | | |
|  |  | | | ENSGALG00000001696 | | S-antigen; retina and pineal gland (arrestin) | | | | | | | | | | < 0.0001 | | | | | | | | | -9.8715 | | | | | | |  | | | | |
|  |  | | | ENSGALG00000007367 | | WAP, follistatin/kazal, immunoglobulin,  kunitz and netrin domain containing 2 | | | | | | | | | | 0.0003 | | | | | | | | | -3.0221 | | | | | | |  | | | | |
|  |  | | | ENSGALG00000016553 | | transmembrane protein 27 | | | | | | | | | | 0.0003 | | | | | | | | | -3.5302 | | | | | | |  | | | | |
|  |  | | | ENSGALG00000001490 | | uncharacterised | | | | | | | | | | 0.0004 | | | | | | | | | -3.2100 | | | | | | |  | | | | |
|  |  | | | ENSGALG00000016020 | | chloride intracellular channel 6 | | | | | | | | | | 0.0004 | | | | | | | | | -3.3355 | | | | | | |  | | | | |
|  |  | | | ENSGALG00000000733 | | myosin VIIA | | | | | | | | | | 0.0004 | | | | | | | | | -2.8969 | | | | | | |  | | | | |
|  |  | | | ENSGALG00000016017 | | solute carrier family 4, sodium borate transporter, member 11 | | | | | | | | | | 0.0007 | | | | | | | | | -2.7615 | | | | | | |  | | | | |
|  |  | | | ENSGALG00000012908 | | solute carrier family 13 (sodium/sulfate symporters), member 4 | | | | | | | | | | 0.0014 | | | | | | | | | -3.7024 | | | | | | |  | | | | |
|  |  | | | ENSGALG00000014967 | | synaptic vesicle glycoprotein 2C | | | | | | | | | | 0.0018 | | | | | | | | | -2.4284 | | | | | | |  | | | | |
|  |  | | | ENSGALG00000001063 | | PR domain containing 16 | | | | | | | | | | 0.0030 | | | | | | | | | -2.2823 | | | | | | |  | | | | |
|  |  | | | ENSGALG00000017343 | | folate receptor 1 (adult) | | | | | | | | | | 0.0031 | | | | | | | | | -2.3754 | | | | | | |  | | | | |
|  |  | | | ENSGALG00000001115 | | membrane metallo-endopeptidase-like 1 | | | | | | | | | | 0.0032 | | | | | | | | | -2.4286 | | | | | | |  | | | | |
|  |  | | | ENSGALG00000018557 | | similar to extracellular-superoxide dismutase (EC 1.15.1.1); superoxide dismutase 3, extracellular | | | | | | | | | | 0.0032 | | | | | | | | | -2.8380 | | | | | | |  | | | | |
|  |  | | | ENSGALG00000011369 | | LIM homeobox 8 | | | | | | | | | | 0.0033 | | | | | | | | | -3.2484 | | | | | | |  | | | | |
|  |  | | | ENSGALG00000015419 | | proenkephalin | | | | | | | | | | 0.0043 | | | | | | | | | -2.6196 | | | | | | |  | | | | |
|  |  | | | ENSGALG00000005628 | | collagen, type IX, alpha 3 | | | | | | | | | | 0.0065 | | | | | | | | | -2.7236 | | | | | | |  | | | | |
|  |  | | | ENSGALG00000017068 | | klotho | | | | | | | | | | 0.0072 | | | | | | | | | -2.2514 | | | | | | |  | | | | |
|  | **Contrast:**  **CB *vs* BC** (1^st^ class *vs* 2^nd^ class) | | | | | | | | | | | | | | | | | | | | | | | | | | | | | | | | | | | |
|  | *(1) Top 20 up-regulated genes under 1*^st^ *class* | | | | | | | | | | | | | | | | | | | | | | | | | | | | |  | |  | | | | |
|  |  | | | Ensembl ID | | | | Description | | | | | | | | FDR | | | | | | | | | FC | | | | | | | | | |  | |
|  |  | | | ENSGALG00000015143 | | | | transthyretin | | | | | | | | 0.0010 | | | | | | | | | 4.3747 | | | | | | | | | |  | |
|  |  | | | ENSGALG00000014117 | | | | arginine vasopressin (neurophysin II, antidiuretic hormone,  diabetes insipidus, neurohypophyseal) | | | | | | | | 0.0020 | | | | | | | | | 4.2093 | | | | | | | | | |  | |
|  |  | | | ENSGALG00000001696 | | | | S-antigen; retina and pineal gland (arrestin) | | | | | | | | 0.0373 | | | | | | | | | 2.7064 | | | | | | | | | |  | |
|  |  | | | ENSGALG00000020975 | | | | transmembrane protein 233 | | | | | | | | 0.0465 | | | | | | | | | 1.7710 | | | | | | | | | |  | |
|  | *(2) Top 20 down-regulated genes under 1*^st^ *class* | | | | | | | | | | | | | | |  | | | | | | | | |  | | | | | | | | | |  | |
|  |  | | | Ensembl ID | | | | Description | | | | | | | | FDR | | | | | | | | | FC | | | | | | | | | |  | |
|  |  | | ENSGALG00000004320 | | | | FAT tumor suppressor homolog 2 (Drosophila) | | | | | | | | 0.0020 | | | | | | | | | -2.6134 | | | | | | | | | |  | |  |
|  |  | | ENSGALG00000008723 | | | | complement component 1, q subcomponent-like 3 | | | | | | | | 0.0080 | | | | | | | | | -1.8987 | | | | | | | | | |  | |  |
|  |  | | ENSGALG00000005842 | | | | glycerophosphodiester phosphodiesterase domain containing 2 | | | | | | | | 0.0115 | | | | | | | | | -2.4758 | | | | | | | | | |  | |  |
|  |  | | ENSGALG00000012544 | | | | UDP-N-acetyl-alpha-D-galactosamine: polypeptide N-acetylgalactosaminyltransferase 5 (GalNAc-T5) | | | | | | | | 0.0128 | | | | | | | | | -1.7961 | | | | | | | | | |  | |  |
|  |  | | ENSGALG00000012285 | | | | BAI1-associated protein 2-like 2 | | | | | | | | 0.0320 | | | | | | | | | -1.8686 | | | | | | | | | |  | |  |
|  |  | | ENSGALG00000008135 | | | | SATB homeobox 2 | | | | | | | | 0.0338 | | | | | | | | | -1.5855 | | | | | | | | | |  | |  |
|  |  | | ENSGALG00000009431 | | | | uncharacterised | | | | | | | | 0.0881 | | | | | | | | | -1.6929 | | | | | | | | | |  | |  |
|  |  | | ENSGALG00000005802 | | | | fms-related tyrosine kinase 4 | | | | | | | | 0.0906 | | | | | | | | | -1.3155 | | | | | | | | | |  | |  |
|  |  | | ENSGALG00000016465 | | | | similar to egg envelope component ZPAX | | | | | | | | 0.0907 | | | | | | | | | -1.5696 | | | | | | | | | |  | |  |
|  |  | | ENSGALG00000002945 | | | | chromosome 15 open reading frame 27 | | | | | | | | 0.0948 | | | | | | | | | -1.5645 | | | | | | | | | |  | |  |
|  | **Contrast:**  **BB *vs* BC** (1^st^ class *vs* 2^nd^ class) | | | | | | | | | | | | | | | | | | | | | | | | | | | | | | | | | |  | |
|  | *(1) Top 20 up-regulated genes under 1*^st^ *class* | | | | | | | | | | | | | | | | | | | | | | | | | | | | | | | | | |  | |
|  |  | Ensembl ID | | | | | | Description | | | | | | | | FDR | | | | | | | | | FC | | | | | | | | | |  | |
|  |  | ENSGALG00000008945 | | | | | | nexilin (F actin binding protein) | | | | | | | | 0.0228 | | | | | | | | | | | 2.8774 | | | | | | | |  | |
|  |  | ENSGALG00000009012 | | | | | | zinc finger protein 533 | | | | | | | | 0.0242 | | | | | | | | | | | 3.2279 | | | | | | | |  | |
|  |  | ENSGALG00000004320 | | | | | | FAT tumor suppressor homolog 2 (Drosophila) | | | | | | | | 0.0284 | | | | | | | | | | | 2.6243 | | | | | | | |  | |
|  |  | ENSGALG00000011262 | | | | | | potassium voltage-gated channel, subfamily H (eag-related), member 8 | | | | | | | | 0.0293 | | | | | | | | | | | 2.2552 | | | | | | | |  | |
|  |  | ENSGALG00000003894 | | | | | | cerebellin 1 precursor | | | | | | | | 0.0350 | | | | | | | | | | | 2.9906 | | | | | | | |  | |
|  |  | ENSGALG00000004527 | | | | | | hypothetical protein LOC776119;  unc-13 homolog C (C. elegans); similar to Munc13-3 | | | | | | | | 0.0364 | | | | | | | | | | | 3.0010 | | | | | | | |  | |
|  |  | ENSGALG00000000920 | | | | | | cingulin | | | | | | | | 0.0373 | | | | | | | | | | | 2.9528 | | | | | | | |  | |
|  |  | ENSGALG00000008193 | | | | | | reelin | | | | | | | | 0.0379 | | | | | | | | | | | 2.6058 | | | | | | | |  | |
|  |  | ENSGALG00000023430 | | | | | | uncharacterised | | | | | | | | 0.0394 | | | | | | | | | | | 3.0591 | | | | | | | |  | |
|  |  | ENSGALG00000006811 | | | | | | Zic family member 1 (odd-paired homolog, Drosophila) | | | | | | | | 0.0404 | | | | | | | | | | | 2.9334 | | | | | | | |  | |
|  |  | ENSGALG00000012226 | | | | | | chromosome 7 open reading frame 16 | | | | | | | | 0.0409 | | | | | | | | | | | 2.1956 | | | | | | | |  | |
|  |  | ENSGALG00000003354 | | | | | | potassium voltage-gated channel, subfamily H (eag-related), member 4 | | | | | | | | 0.0471 | | | | | | | | | | | 1.9794 | | | | | | | |  | |
|  |  | ENSGALG00000015018 | | | | | | calsequestrin 2 (cardiac muscle) | | | | | | | | 0.0485 | | | | | | | | | | | 2.4365 | | | | | | | |  | |
|  |  | ENSGALG00000005842 | | | | | | glycerophosphodiester phosphodiesterase domain containing 2 | | | | | | | | 0.0486 | | | | | | | | | | | 2.3907 | | | | | | | |  | |
|  |  | ENSGALG00000009431 | | | | | | uncharacterised | | | | | | | | 0.0490 | | | | | | | | | | | 2.5611 | | | | | | | |  | |
|  |  | ENSGALG00000002161 | | | | | | similar to MGC80370 protein | | | | | | | | 0.0495 | | | | | | | | | | | 2.0372 | | | | | | | |  | |
|  |  | ENSGALG00000010934 | | | | | | InaD-like (Drosophila) | | | | | | | | 0.0499 | | | | | | | | | | | 2.5613 | | | | | | | |  | |
|  |  | ENSGALG00000001172 | | | | | | kainate binding protein | | | | | | | | 0.0506 | | | | | | | | | | | 2.6320 | | | | | | | |  | |
|  |  | ENSGALG00000000681 | | | | | | similar to PAK3 protein; p21 (CDKN1A)-activated kinase 3; p21 protein (Cdc42/Rac)-activated kinase 1 | | | | | | | | 0.0522 | | | | | | | | | | | 1.9490 | | | | | | | |  | |
|  |  | ENSGALG00000005409 | | | | | | LIM homeobox 1 | | | | | | | | 0.0526 | | | | | | | | | | | 2.6154 | | | | | | | |  | |
|  |  |  | | | | | |  | | | | | | | |  | | | | | | | | | | |  | | | | | | | |  | |
|  | *(2) Top 20 down-regulated genes under 1*^st^ *class* | | | | | | | | | | | | | | | |  | | | | | | | | | | |  | | | | | | | | |
|  |  | Ensembl ID | | | | | | | Description | | | | | | | | FDR | | | | | | | | | FC | | | | | | | | | | |
|  |  | ENSGALG00000006473 | | | | | | | plexin A4, B | | | | | | | | 0.0160 | | | | | | | | | -1.8857 | | | | | | | | | | |
|  |  | ENSGALG00000017173 | | | | | | | guanylate cyclase 1, soluble, alpha 2 | | | | | | | | 0.0292 | | | | | | | | | -1.7813 | | | | | | | | | | |
|  |  | ENSGALG00000005347 | | | | | | | similar to ADAMTS18 protein | | | | | | | | 0.0307 | | | | | | | | | -1.9930 | | | | | | | | | | |
|  |  | ENSGALG00000007871 | | | | | | | similar to similar to glutamate transporter 1 variant;  solute carrier family 1 (glial high affinity glutamate transporter), member 2 | | | | | | | | 0.0310 | | | | | | | | | -1.8935 | | | | | | | | | | |
|  |  | ENSGALG00000014967 | | | | | | | synaptic vesicle glycoprotein 2C | | | | | | | | 0.0310 | | | | | | | | | -1.8210 | | | | | | | | | | |
|  |  | ENSGALG00000011577 | | | | | | | contactin associated protein-like 5 | | | | | | | | 0.0329 | | | | | | | | | -1.7609 | | | | | | | | | | |
|  |  | ENSGALG00000008885 | | | | | | | phosphodiesterase 1A, calmodulin-dependent | | | | | | | | 0.0362 | | | | | | | | | -1.7029 | | | | | | | | | | |
|  |  | ENSGALG00000006485 | | | | | | | uncharacterised | | | | | | | | 0.0378 | | | | | | | | | -1.7609 | | | | | | | | | | |
|  |  | ENSGALG00000005802 | | | | | | | fms-related tyrosine kinase 4 | | | | | | | | 0.0405 | | | | | | | | | -1.6421 | | | | | | | | | | |
|  |  | ENSGALG00000001608 | | | | | | | unc-5 homolog D (C. elegans) | | | | | | | | 0.0483 | | | | | | | | | -1.6998 | | | | | | | | | | |
|  |  | ENSGALG00000015080 | | | | | | | solute carrier family 24 (sodium/potassium/calcium exchanger), member 2 | | | | | | | | 0.0513 | | | | | | | | | -1.6969 | | | | | | | | | | |
|  |  | ENSGALG00000009737 | | | | | | | tachykinin, precursor 1 (substance K, substance P, neurokinin 1, neurokinin 2,  neuromedin L, neurokinin alpha, neuropeptide K, neuropeptide gamma) | | | | | | | | 0.0525 | | | | | | | | | -1.6675 | | | | | | | | | | |
|  |  | ENSGALG00000017281 | | | | | | | uncharacterised | | | | | | | | 0.0525 | | | | | | | | | -1.6908 | | | | | | | | | | |
|  |  | ENSGALG00000012248 | | | | | | | similar to MAP3K9 protein; mitogen-activated protein kinase kinase kinase 9 | | | | | | | | 0.0528 | | | | | | | | | -1.6407 | | | | | | | | | | |
|  |  | ENSGALG00000008723 | | | | | | | complement component 1, q subcomponent-like 3 | | | | | | | | 0.0541 | | | | | | | | | -1.6211 | | | | | | | | | | |
|  |  | ENSGALG00000002799 | | | | | | | chromosome 2 open reading frame 21 | | | | | | | | 0.0554 | | | | | | | | | -1.6813 | | | | | | | | | | |
|  |  | ENSGALG00000008544 | | | | | | | similar to Na+/Ca2+ exchanger; solute carrier family 8 (sodium/calcium exchanger), member 1 | | | | | | | | 0.0567 | | | | | | | | | -1.6168 | | | | | | | | | | |
|  |  | ENSGALG00000000184 | | | | | | | solute carrier family 27 (fatty acid transporter), member 6 | | | | | | | | 0.0568 | | | | | | | | | -1.6835 | | | | | | | | | | |
|  |  | ENSGALG00000015737 | | | | | | | neural cell adhesion molecule 2 | | | | | | | | 0.0569 | | | | | | | | | -1.6369 | | | | | | | | | | |
|  |  | ENSGALG00000007993 | | | | | | | doublecortex | | | | | | | | 0.0569 | | | | | | | | | -1.6449 | | | | | | | | | | |
|  | **Contrast:**  **BB *vs* CB** (1^st^ class *vs* 2^nd^ class) | | | | | | | | | | | | | | | | | | | | | | | | | | | | | | |  | | | | |
|  | *(1) Top 20 up-regulated genes under 1*^st^ *class* | | | | | | | | | | | | | | | | | | | | | | | | | | | | | | |  | | | | |
|  |  | Ensembl ID | | | | | | | Description | | | | | | | | FDR | | | | | | FC | | | | | | | | | | | | | |
|  |  | ENSGALG00000003894 | | | | | | | cerebellin 1 precursor | | | | | | | | 0.0095 | | | | | | 4.4751 | | | | | | | | | | | | | |
|  |  | ENSGALG00000004320 | | | | | | | FAT tumor suppressor homolog 2 (Drosophila) | | | | | | | | 0.0100 | | | | | | 6.8750 | | | | | | | | | | | | | |
|  |  | ENSGALG00000005842 | | | | | | | glycerophosphodiester phosphodiesterase domain containing 2 | | | | | | | | 0.0108 | | | | | | 5.9259 | | | | | | | | | | | | | |
|  |  | ENSGALG00000012285 | | | | | | | BAI1-associated protein 2-like 2 | | | | | | | | 0.0117 | | | | | | 6.1420 | | | | | | | | | | | | | |
|  |  | ENSGALG00000000920 | | | | | | | cingulin | | | | | | | | 0.0122 | | | | | | 4.6988 | | | | | | | | | | | | | |
|  |  | ENSGALG00000004527 | | | | | | | hypothetical protein LOC776119; unc-13 homolog C (C. elegans); similar to Munc13-3 | | | | | | | | 0.0133 | | | | | | 4.4623 | | | | | | | | | | | | | |
|  |  | ENSGALG00000024278 | | | | | | | uncharacterised | | | | | | | | 0.0164 | | | | | | 3.5586 | | | | | | | | | | | | | |
|  |  | ENSGALG00000006811 | | | | | | | Zic family member 1 (odd-paired homolog, Drosophila) | | | | | | | | 0.0181 | | | | | | 3.4901 | | | | | | | | | | | | | |
|  |  | ENSGALG00000008945 | | | | | | | nexilin (F actin binding protein) | | | | | | | | 0.0220 | | | | | | 2.8214 | | | | | | | | | | | | | |
|  |  | ENSGALG00000009012 | | | | | | | zinc finger protein 533 | | | | | | | | 0.0238 | | | | | | 3.4529 | | | | | | | | | | | | | |
|  |  | ENSGALG00000009431 | | | | | | | uncharacterised | | | | | | | | 0.0245 | | | | | | 4.3605 | | | | | | | | | | | | | |
|  |  | ENSGALG00000012226 | | | | | | | chromosome 7 open reading frame 16 | | | | | | | | 0.0254 | | | | | | 3.1649 | | | | | | | | | | | | | |
|  |  | ENSGALG00000008908 | | | | | | | neurogenic differentiation 1 | | | | | | | | 0.0258 | | | | | | 2.5441 | | | | | | | | | | | | | |
|  |  | ENSGALG00000003354 | | | | | | | potassium voltage-gated channel, subfamily H (eag-related), member 4 | | | | | | | | 0.0268 | | | | | | 2.1464 | | | | | | | | | | | | | |
|  |  | ENSGALG00000016988 | | | | | | | chromosome 13 open reading frame 18 | | | | | | | | 0.0328 | | | | | | 3.3360 | | | | | | | | | | | | | |
|  |  | ENSGALG00000008193 | | | | | | | reelin | | | | | | | | 0.0331 | | | | | | 3.6453 | | | | | | | | | | | | | |
|  |  | ENSGALG00000023430 | | | | | | | uncharacterised | | | | | | | | 0.0333 | | | | | | 3.8019 | | | | | | | | | | | | | |
|  |  | ENSGALG00000015018 | | | | | | | calsequestrin 2 (cardiac muscle) | | | | | | | | 0.0335 | | | | | | 3.2215 | | | | | | | | | | | | | |
|  |  | ENSGALG00000003149 | | | | | | | inositol 1,4,5-triphosphate receptor, type 3 | | | | | | | | 0.0336 | | | | | | 2.9759 | | | | | | | | | | | | | |
|  |  | ENSGALG00000015778 | | | | | | | gamma-aminobutyric acid (GABA) receptor, rho 1 | | | | | | | | 0.0337 | | | | | | 3.5362 | | | | | | | | | | | | | |
|  | *(2) Top 20 down-regulated genes under 1*^st^ *class* | | | | | | | | | | | | | | | |  | | | | | |  | | | | | | | | | | | | | |
|  |  | Ensembl ID | | | | | | | Description | | | | | | | | FDR | | | | | | FC | | | | | | | | | | | | | |
|  |  | ENSGALG00000015143 | | | | | | | transthyretin | | | | | | | | 0.0020 | | | | | | -6.7550 | | | | | | | | | | | | | |
|  |  | ENSGALG00000001696 | | | | | | | S-antigen; retina and pineal gland (arrestin) | | | | | | | | 0.0590 | | | | | | -3.3422 | | | | | | | | | | | | | |
|  | **(b) Hypothalamus** | | | | | | | | | | | |  | | | | | | | | | | | | | | | | | | | | | | | |
|  | **Contrast:**  **BC *vs* CC** (1^st^ class *vs* 2^nd^ class) | | | | | | | | | | | | | | | | | | | | | | | | | | | | | | | | | | | |
|  | *(1) Top 20 up-regulated genes under 1*^st^ *class* | | | | | | | | | | | | | | | | | | | | | | | | | | | | | | | | | | | |
|  |  | Ensembl ID | | | | | | | | | | Description | | | | | FDR | | | | | | FC | | | | | | | | | | | | | |
|  |  | ENSGALG00000009740 | | | | | | | | | | PR domain containing 16 | | | | | 0.0000 | | | | | | 3.4815 | | | | | | | | | | | | | |
|  |  | ENSGALG00000011369 | | | | | | | | | | LIM homeobox 8 | | | | | 0.0005 | | | | | | 2.3797 | | | | | | | | | | | | | |
|  |  | ENSGALG00000015529 | | | | | | | | | | Wolfram syndrome 1 (wolframin) | | | | | 0.0007 | | | | | | 2.4260 | | | | | | | | | | | | | |
|  |  | ENSGALG00000017194 | | | | | | | | | | transient receptor potential cation channel, subfamily C, member 6 | | | | | 0.0016 | | | | | | 2.0623 | | | | | | | | | | | | | |
|  |  | ENSGALG00000007980 | | | | | | | | | | phosphodiesterase 1A, calmodulin-dependent | | | | | 0.0016 | | | | | | 2.2307 | | | | | | | | | | | | | |
|  |  | ENSGALG00000001074 | | | | | | | | | | LIM homeobox 6 | | | | | 0.0018 | | | | | | 2.1270 | | | | | | | | | | | | | |
|  |  | ENSGALG00000001347 | | | | | | | | | | PR domain containing 12 | | | | | 0.0020 | | | | | | 2.0098 | | | | | | | | | | | | | |
|  |  | ENSGALG00000017044 | | | | | | | | | | transient receptor potential cation channel, subfamily C, member 4 | | | | | 0.0041 | | | | | | 2.1187 | | | | | | | | | | | | | |
|  |  | ENSGALG00000001063 | | | | | | | | | | hypothetical gene supported by CR385622 | | | | | 0.0041 | | | | | | 1.9595 | | | | | | | | | | | | | |
|  |  | ENSGALG00000007139 | | | | | | | | | | potassium voltage-gated channel, subfamily G, member 1 | | | | | 0.0051 | | | | | | 2.0885 | | | | | | | | | | | | | |
|  |  | ENSGALG00000009853 | | | | | | | | | | forkhead box G1 | | | | | 0.0055 | | | | | | 1.9958 | | | | | | | | | | | | | |
|  |  | ENSGALG00000014804 | | | | | | | | | | thrombospondin 4 | | | | | 0.0066 | | | | | | 1.8216 | | | | | | | | | | | | | |
|  |  | ENSGALG00000003895 | | | | | | | | | | family with sequence similarity 107, member A | | | | | 0.0069 | | | | | | 2.0431 | | | | | | | | | | | | | |
|  |  | ENSGALG00000015857 | | | | | | | | | | carbonic anhydrase III, muscle specific | | | | | 0.0072 | | | | | | 1.8534 | | | | | | | | | | | | | |
|  |  | ENSGALG00000014843 | | | | | | | | | | tumor protein D52-like 1 | | | | | 0.0076 | | | | | | 1.9745 | | | | | | | | | | | | | |
|  |  | ENSGALG00000008885 | | | | | | | | | | similar to RAS guanyl releasing protein 1 (calcium and DAG-regulated);  RAS guanyl releasing protein 1 (calcium and DAG-regulated) | | | | | 0.0084 | | | | | | 1.8739 | | | | | | | | | | | | | |
|  |  | ENSGALG00000009799 | | | | | | | | | | Meis homeobox 2 | | | | | 0.0085 | | | | | | 1.8950 | | | | | | | | | | | | | |
|  |  | ENSGALG00000011170 | | | | | | | | | | WAS/WASL interacting protein family, member 3 | | | | | 0.0132 | | | | | | 1.8970 | | | | | | | | | | | | | |
|  |  | ENSGALG00000014484 | | | | | | | | | | uncharacterised | | | | | 0.0207 | | | | | | 1.8127 | | | | | | | | | | | | | |
|  |  | ENSGALG00000016866 | | | | | | | | | | fibroblast growth factor 14 | | | | | 0.0358 | | | | | | 1.6506 | | | | | | | | | | | | | |
|  |  |  | | | | | | | | | |  | | | | |  | | | | | |  | | | | | | | | | | | | | |
|  | *(2) Top 20 down-regulated genes under 1*^st^ *class* | | | | | | | | | | | | | | | |  | | | | | |  | | | | | | | | | | | | | |
|  |  | Ensembl ID | | | | | | | | | | Description | | | | | FDR | | | | | | FC | | | | | | | | | | | | | |
|  |  | ENSGALG00000000507 | | | | | | | | | | copine VII | | | | | 0.0080 | | | | | | -2.7013 | | | | | | | | | | | | | |
|  |  | ENSGALG00000003839 | | | | | | | | | | glutamate receptor, metabotropic 2 | | | | | 0.0110 | | | | | | -2.4097 | | | | | | | | | | | | | |
|  |  | ENSGALG00000009095 | | | | | | | | | | luteinizing hormone/choriogonadotropin receptor | | | | | 0.0118 | | | | | | -2.2222 | | | | | | | | | | | | | |
|  |  | ENSGALG00000008883 | | | | | | | | | | transcription factor 7-like 2 (T-cell specific, HMG-box) | | | | | 0.0120 | | | | | | -2.2245 | | | | | | | | | | | | | |
|  |  | ENSGALG00000004919 | | | | | | | | | | uncharacterised | | | | | 0.0135 | | | | | | -3.0885 | | | | | | | | | | | | | |
|  |  | ENSGALG00000009791 | | | | | | | | | | prospero-related homeobox 1 | | | | | 0.0185 | | | | | | -1.9404 | | | | | | | | | | | | | |
|  |  | ENSGALG00000002331 | | | | | | | | | | calbindin 2, 29kDa (calretinin) | | | | | 0.0207 | | | | | | -1.8891 | | | | | | | | | | | | | |
|  |  | ENSGALG00000024278 | | | | | | | | | | uncharacterised | | | | | 0.0207 | | | | | | -1.6992 | | | | | | | | | | | | | |
|  |  | ENSGALG00000006838 | | | | | | | | | | similar to iron binding protein | | | | | 0.0211 | | | | | | -1.6637 | | | | | | | | | | | | | |
|  |  | ENSGALG00000003894 | | | | | | | | | | cerebellin 1 precursor | | | | | 0.0213 | | | | | | -1.7183 | | | | | | | | | | | | | |
|  |  | ENSGALG00000003562 | | | | | | | | | | neuronal pentraxin II | | | | | 0.0231 | | | | | | -1.8905 | | | | | | | | | | | | | |
|  |  | ENSGALG00000016600 | | | | | | | | | | proopiomelanocortin (adrenocorticotropin/ beta-lipotropin/ alpha-melanocyte stimulating hormone/  beta-melanocyte stimulating hormone/ beta-endorphin) | | | | | 0.0234 | | | | | | -1.5726 | | | | | | | | | | | | | |
|  |  | ENSGALG00000014477 | | | | | | | | | | CD4 molecule | | | | | 0.0234 | | | | | | -2.0514 | | | | | | | | | | | | | |
|  |  | ENSGALG00000013193 | | | | | | | | | | iroquois homeobox 2 | | | | | 0.0304 | | | | | | -1.9073 | | | | | | | | | | | | | |
|  |  | ENSGALG00000002223 | | | | | | | | | | LIM homeobox 9 | | | | | 0.0343 | | | | | | -1.8817 | | | | | | | | | | | | | |
|  |  | ENSGALG00000016083 | | | | | | | | | | similar to Angiopoietin 1; angiopoietin 1 | | | | | 0.0350 | | | | | | -1.8203 | | | | | | | | | | | | | |
|  |  | ENSGALG00000021567 | | | | | | | | | | uncharacterised | | | | | 0.0353 | | | | | | -1.7714 | | | | | | | | | | | | | |
|  |  | ENSGALG00000008735 | | | | | | | | | | beaded filament structural protein 1, filensin | | | | | 0.0377 | | | | | | -1.8273 | | | | | | | | | | | | | |
|  |  | ENSGALG00000015824 | | | | | | | | | | glycoprotein hormones, alpha polypeptide | | | | | 0.0536 | | | | | | -1.6450 | | | | | | | | | | | | | |
|  |  | ENSGALG00000016904 | | | | | | | | | | SLIT and NTRK-like family, member 6 | | | | | 0.0555 | | | | | | -1.7264 | | | | | | | | | | | | | |
|  | **Contrast:**  **CB *vs* CC** (1^st^ class *vs* 2^nd^ class) | | | | | | | | | | | | | | | | | | | | | | | | | | | | | | | | | | | |
|  | *(1) Top 20 up-regulated genes under 1*^st^ *class* | | | | | | | | | | | | | | | | | | | | | | | | | | | | | | | | | | | |
|  |  | Ensembl ID | | | | | | | | | | Description | | | | | FDR | | | | | | FC | | | | | | | | | | | | | |
|  |  | ENSGALG00000008135 | | | | | | | | | | SATB homeobox 2 | | | | | 0.0037 | | | | | | 3.0762 | | | | | | | | | | | | | |
|  |  | ENSGALG00000014907 | | | | | | | | | | discoidin, CUB and LCCL domain containing 1 | | | | | 0.0038 | | | | | | 3.2824 | | | | | | | | | | | | | |
|  |  | ENSGALG00000002821 | | | | | | | | | | gastrin-releasing peptide | | | | | 0.0040 | | | | | | 3.0643 | | | | | | | | | | | | | |
|  |  | ENSGALG00000012235 | | | | | | | | | | neurogenic differentiation 6 | | | | | 0.0055 | | | | | | 2.9570 | | | | | | | | | | | | | |
|  |  | ENSGALG00000006406 | | | | | | | | | | bombesin-like receptor 3 | | | | | 0.0122 | | | | | | 2.8584 | | | | | | | | | | | | | |
|  |  | ENSGALG00000014011 | | | | | | | | | | lymphoid-restricted membrane protein | | | | | 0.0168 | | | | | | 2.4149 | | | | | | | | | | | | | |
|  |  | ENSGALG00000001282 | | | | | | | | | | gamma-aminobutyric acid (GABA) A receptor, delta | | | | | 0.0170 | | | | | | 2.3082 | | | | | | | | | | | | | |
|  |  | ENSGALG00000015626 | | | | | | | | | | regulator of G-protein signalling 12 | | | | | 0.0171 | | | | | | 2.5448 | | | | | | | | | | | | | |
|  |  | ENSGALG00000004270 | | | | | | | | | | aldehyde dehydrogenase 1 family, member A2 | | | | | 0.0175 | | | | | | 2.5447 | | | | | | | | | | | | | |
|  |  | ENSGALG00000007141 | | | | | | | | | | leucine-rich repeat kinase 1 | | | | | 0.0184 | | | | | | 2.6677 | | | | | | | | | | | | | |
|  |  | ENSGALG00000011122 | | | | | | | | | | uncharacterised | | | | | 0.0208 | | | | | | 2.7867 | | | | | | | | | | | | | |
|  |  | ENSGALG00000015271 | | | | | | | | | | filamin A interacting protein 1-like | | | | | 0.0247 | | | | | | 2.3942 | | | | | | | | | | | | | |
|  |  | ENSGALG00000014645 | | | | | | | | | | MADS box transcription enhancer factor 2, polypeptide C (myocyte enhancer factor 2C) | | | | | 0.0321 | | | | | | 2.2620 | | | | | | | | | | | | | |
|  |  | ENSGALG00000008885 | | | | | | | | | | phosphodiesterase 1A, calmodulin-dependent | | | | | 0.0367 | | | | | | 2.2236 | | | | | | | | | | | | | |
|  |  | ENSGALG00000016920 | | | | | | | | | | LIM domain 7 | | | | | 0.0371 | | | | | | 2.1656 | | | | | | | | | | | | | |
|  |  | ENSGALG00000019842 | | | | | | | | | | transcription factor AP-2delta | | | | | 0.0374 | | | | | | 2.2918 | | | | | | | | | | | | | |
|  |  | ENSGALG00000023441 | | | | | | | | | | reticulon 4 receptor-like 2 | | | | | 0.0406 | | | | | | 1.9892 | | | | | | | | | | | | | |
|  |  | ENSGALG00000018942 | | | | | | | | | | neurogranin  (protein kinase C substrate, RC3) | | | | | 0.0765 | | | | | | 1.9119 | | | | | | | | | | | | | |
|  |  | ENSGALG00000020515 | | | | | | | | | | uncharacterised | | | | | 0.0794 | | | | | | 1.9019 | | | | | | | | | | | | | |
|  |  | ENSGALG00000012254 | | | | | | | | | | potassium inwardly-rectifying channel, subfamily J, member 4 | | | | | 0.0853 | | | | | | 2.0097 | | | | | | | | | | | | | |
|  |  |  | | | | | | | | | |  | | | | |  | | | | | |  | | | | | | | | | | | | | |
|  | *(2) Top 20 down-regulated genes under 1*^st^ *class* | | | | | | | | | | | | | | | |  | | | | | |  | | | | | | | | | | | | | |
|  |  | Ensembl ID | | | | | | | | | | Description | | | | | FDR | | | | | | FC | | | | | | | | | | | | | |
|  |  | ENSGALG00000004919 | | | | | | | | | | uncharacterised | | | | | 0.0020 | | | | | | -4.6928 | | | | | | | | | | | | | |
|  |  | ENSGALG00000015143 | | | | | | | | | | transthyretin | | | | | 0.0065 | | | | | | -4.1969 | | | | | | | | | | | | | |
|  |  | ENSGALG00000004572 | | | | | | | | | | natriuretic peptide precursor C | | | | | 0.0467 | | | | | | -2.2076 | | | | | | | | | | | | | |
|  |  | ENSGALG00000003894 | | | | | | | | | | cerebellin 1 precursor | | | | | 0.0510 | | | | | | -2.0654 | | | | | | | | | | | | | |
|  |  | ENSGALG00000012464 | | | | | | | | | | SOUL protein | | | | | 0.0740 | | | | | | -2.1329 | | | | | | | | | | | | | |
|  | **Contrast:**  **BB *vs* CC** (1^st^ class *vs* 2^nd^ class) | | | | | | | | | | | | | | | | | | | | | | | | | | | | | | | | | |  | |
|  | *(1) Top 20 up-regulated genes under 1*^st^ *class* | | | | | | | | | | | | | | | | | | | | | | | | | | | | | | | | | |  | |
|  |  | Ensembl ID | | | | | | | | | Description | | | | | FDR | | | | | | FC | | | | | | | | | | | | |  | |
|  |  | ENSGALG00000006406 | | | | | | | | | bombesin-like receptor 3 | | | | | 0.0000 | | | | | | 3.3041 | | | | | | | | | | | | |  | |
|  |  | ENSGALG00000008135 | | | | | | | | | SATB homeobox 2 | | | | | 0.0000 | | | | | | 4.4087 | | | | | | | | | | | | |  | |
|  |  | ENSGALG00000011122 | | | | | | | | | uncharacterised | | | | | 0.0000 | | | | | | 3.4907 | | | | | | | | | | | | |  | |
|  |  | ENSGALG00000012235 | | | | | | | | | neurogenic differentiation 6 | | | | | 0.0000 | | | | | | 4.0161 | | | | | | | | | | | | |  | |
|  |  | ENSGALG00000014907 | | | | | | | | | discoidin, CUB and LCCL domain containing 1 | | | | | 0.0000 | | | | | | 4.2754 | | | | | | | | | | | | |  | |
|  |  | ENSGALG00000014645 | | | | | | | | | MADS box transcription enhancer factor 2, polypeptide C (myocyte enhancer factor 2C) | | | | | 0.0001 | | | | | | 2.5816 | | | | | | | | | | | | |  | |
|  |  | ENSGALG00000001282 | | | | | | | | | gamma-aminobutyric acid (GABA) A receptor, delta | | | | | 0.0001 | | | | | | 2.7574 | | | | | | | | | | | | |  | |
|  |  | ENSGALG00000004270 | | | | | | | | | aldehyde dehydrogenase 1 family, member A2 | | | | | 0.0001 | | | | | | 2.9466 | | | | | | | | | | | | |  | |
|  |  | ENSGALG00000015626 | | | | | | | | | regulator of G-protein signalling 12 | | | | | 0.0001 | | | | | | 2.7538 | | | | | | | | | | | | |  | |
|  |  | ENSGALG00000007141 | | | | | | | | | leucine-rich repeat kinase 1 | | | | | 0.0001 | | | | | | 2.8333 | | | | | | | | | | | | |  | |
|  |  | ENSGALG00000008885 | | | | | | | | | phosphodiesterase 1A, calmodulin-dependent | | | | | 0.0002 | | | | | | 2.5087 | | | | | | | | | | | | |  | |
|  |  | ENSGALG00000009740 | | | | | | | | | similar to RAS guanyl releasing protein 1 (calcium and DAG-regulated);  RAS guanyl releasing protein 1 (calcium and DAG-regulated) | | | | | 0.0002 | | | | | | 2.5902 | | | | | | | | | | | | |  | |
|  |  | ENSGALG00000019842 | | | | | | | | | transcription factor AP-2delta | | | | | 0.0002 | | | | | | 2.9304 | | | | | | | | | | | | |  | |
|  |  | ENSGALG00000014011 | | | | | | | | | lymphoid-restricted membrane protein | | | | | 0.0004 | | | | | | 2.5451 | | | | | | | | | | | | |  | |
|  |  | ENSGALG00000015271 | | | | | | | | | filamin A interacting protein 1-like | | | | | 0.0006 | | | | | | 2.3232 | | | | | | | | | | | | |  | |
|  |  | ENSGALG00000009853 | | | | | | | | | forkhead box G1 | | | | | 0.0006 | | | | | | 2.3101 | | | | | | | | | | | | |  | |
|  |  | ENSGALG00000011721 | | | | | | | | | A kinase (PRKA) anchor protein 5 | | | | | 0.0008 | | | | | | 2.2408 | | | | | | | | | | | | |  | |
|  |  | ENSGALG00000018942 | | | | | | | | | neurogranin (protein kinase C substrate, RC3) | | | | | 0.0008 | | | | | | 2.2257 | | | | | | | | | | | | |  | |
|  |  | ENSGALG00000013154 | | | | | | | | | solute carrier organic anion transporter family, member 1C1 | | | | | 0.0010 | | | | | | 2.3958 | | | | | | | | | | | | |  | |
|  |  | ENSGALG00000011254 | | | | | | | | | SATB homeobox 1 | | | | | 0.0013 | | | | | | 2.1602 | | | | | | | | | | | | |  | |
| *(2) Top 20 down-regulated genes under 1*^st^ *class* | | | | | | | | | | | | | |  | | | | | | |  | | | | | | | | | | |  | | | | |
|  |  | Ensembl ID | | | | | | | | | Description | | | | | FDR | | | | | | FC | | | | | | | | | | | | |  | |
|  |  | ENSGALG00000004919 | | | | | | | | | uncharacterised | | | | | 0.0000 | | | | | | -4.8313 | | | | | | | | | | | | |  | |
|  |  | ENSGALG00000009791 | | | | | | | | | prospero-related homeobox 1 | | | | | 0.0425 | | | | | | -1.8623 | | | | | | | | | | | | |  | |
|  |  | ENSGALG00000014477 | | | | | | | | | CD4 molecule | | | | | 0.0427 | | | | | | -1.8734 | | | | | | | | | | | | |  | |
|  |  | ENSGALG00000008900 | | | | | | | | | tetra-peptide repeat homeobox-like | | | | | 0.0544 | | | | | | -1.5701 | | | | | | | | | | | | |  | |
|  |  | ENSGALG00000009095 | | | | | | | | | luteinizing hormone/choriogonadotropin receptor | | | | | 0.0555 | | | | | | -1.7583 | | | | | | | | | | | | |  | |
|  |  | ENSGALG00000002331 | | | | | | | | | calbindin 2, 29kDa (calretinin) | | | | | 0.0565 | | | | | | -1.6316 | | | | | | | | | | | | |  | |
|  |  | ENSGALG00000016904 | | | | | | | | | SLIT and NTRK-like family, member 6 | | | | | 0.0571 | | | | | | -1.7489 | | | | | | | | | | | | |  | |
|  |  | ENSGALG00000002223 | | | | | | | | | LIM homeobox 9 | | | | | 0.0597 | | | | | | -1.7723 | | | | | | | | | | | | |  | |
|  |  | ENSGALG00000008883 | | | | | | | | | transcription factor 7-like 2 (T-cell specific, HMG-box) | | | | | 0.0624 | | | | | | -1.7920 | | | | | | | | | | | | |  | |
|  |  | ENSGALG00000006236 | | | | | | | | | tryptophan hydroxylase 1 | | | | | 0.0693 | | | | | | -1.4720 | | | | | | | | | | | | |  | |
|  |  | ENSGALG00000013193 | | | | | | | | | iroquois homeobox 2 | | | | | 0.0694 | | | | | | -1.7682 | | | | | | | | | | | | |  | |
|  |  | ENSGALG00000012911 | | | | | | | | | synaptotagmin X | | | | | 0.0700 | | | | | | -1.7574 | | | | | | | | | | | | |  | |
|  |  | ENSGALG00000012495 | | | | | | | | | uncharacterised | | | | | 0.0822 | | | | | | -1.6020 | | | | | | | | | | | | |  | |
|  |  | ENSGALG00000007772 | | | | | | | | | cerebellin 4 precursor | | | | | 0.0841 | | | | | | -1.6096 | | | | | | | | | | | | |  | |
|  |  | ENSGALG00000008671 | | | | | | | | | ST8 alpha-N-acetyl-neuraminide alpha-2,8-sialyltransferase 6 | | | | | 0.0859 | | | | | | -1.5017 | | | | | | | | | | | | |  | |
|  |  | ENSGALG00000010461 | | | | | | | | | early B-cell factor 3 | | | | | 0.0869 | | | | | | -1.5718 | | | | | | | | | | | | |  | |
|  |  | ENSGALG00000010402 | | | | | | | | | prostaglandin-D synthase | | | | | 0.0909 | | | | | | -1.4618 | | | | | | | | | | | | |  | |
|  |  | ENSGALG00000023036 | | | | | | | | | uncharacterised | | | | | 0.0931 | | | | | | -1.5130 | | | | | | | | | | | | |  | |
|  |  | ENSGALG00000006384 | | | | | | | | | interferon-induced protein with tetratricopeptide repeats 5 | | | | | 0.0956 | | | | | | -1.6101 | | | | | | | | | | | | |  | |
|  |  | ENSGALG00000003894 | | | | | | | | | cerebellin 1 precursor | | | | | 0.0983 | | | | | | -1.4688 | | | | | | | | | | | | |  | |
|  | **Contrast:**  **CB *vs* BC** (1^st^ class *vs* 2^nd^ class) | | | | | | | | | | | | | | | | | | | | | | | | | | | | | | | | | | | |
|  | *(1) Top 20 up-regulated genes under 1*^st^ *class* | | | | | | | | | | | | | | | | | | | | | | | | | | | | | |  | | | | | |
|  |  | Ensembl ID | | | | | | | | | Description | | | | | FDR | | | | | | FC | | | | | | | | | | | | |  | |
|  |  | ENSGALG00000002821 | | | | | | | | | gastrin-releasing peptide | | | | | 0.0010 | | | | | | 2.8454 | | | | | | | | | | | | |  | |
|  |  | ENSGALG00000012235 | | | | | | | | | neurogenic differentiation 6 | | | | | 0.0020 | | | | | | 3.7793 | | | | | | | | | | | | |  | |
|  |  | ENSGALG00000008135 | | | | | | | | | SATB homeobox 2 | | | | | 0.0083 | | | | | | 2.8284 | | | | | | | | | | | | |  | |
|  |  | ENSGALG00000007141 | | | | | | | | | leucine-rich repeat kinase 1 | | | | | 0.0172 | | | | | | 2.4867 | | | | | | | | | | | | |  | |
|  |  | ENSGALG00000004270 | | | | | | | | | aldehyde dehydrogenase 1 family, member A2 | | | | | 0.0200 | | | | | | 2.4217 | | | | | | | | | | | | |  | |
|  |  | ENSGALG00000014011 | | | | | | | | | lymphoid-restricted membrane protein | | | | | 0.0200 | | | | | | 2.4001 | | | | | | | | | | | | |  | |
|  |  | ENSGALG00000023441 | | | | | | | | | reticulon 4 receptor-like 2 | | | | | 0.0204 | | | | | | 2.2214 | | | | | | | | | | | | |  | |
|  |  | ENSGALG00000001564 | | | | | | | | | ATPase, Ca++ transporting, ubiquitous | | | | | 0.0205 | | | | | | 2.7068 | | | | | | | | | | | | |  | |
|  |  | ENSGALG00000019842 | | | | | | | | | transcription factor AP-2delta | | | | | 0.0206 | | | | | | 2.4529 | | | | | | | | | | | | |  | |
|  |  | ENSGALG00000014907 | | | | | | | | | discoidin, CUB and LCCL domain containing 1 | | | | | 0.0298 | | | | | | 2.1306 | | | | | | | | | | | | |  | |
|  |  | ENSGALG00000011271 | | | | | | | | | lumican | | | | | 0.0318 | | | | | | 1.8437 | | | | | | | | | | | | |  | |
|  |  | ENSGALG00000011122 | | | | | | | | | uncharacterised | | | | | 0.0322 | | | | | | 1.8973 | | | | | | | | | | | | |  | |
|  |  | ENSGALG00000000507 | | | | | | | | | copine VII | | | | | 0.0328 | | | | | | 2.1166 | | | | | | | | | | | | |  | |
|  |  | ENSGALG00000012254 | | | | | | | | | potassium inwardly-rectifying channel, subfamily J, member 4 | | | | | 0.0328 | | | | | | 2.2816 | | | | | | | | | | | | |  | |
|  |  | ENSGALG00000015271 | | | | | | | | | filamin A interacting protein 1-like | | | | | 0.0342 | | | | | | 2.2372 | | | | | | | | | | | | |  | |
|  |  | ENSGALG00000018942 | | | | | | | | | neurogranin (protein kinase C substrate, RC3) | | | | | 0.0346 | | | | | | 1.8758 | | | | | | | | | | | | |  | |
|  |  | ENSGALG00000003839 | | | | | | | | | glutamate receptor, metabotropic 2 | | | | | 0.0352 | | | | | | 2.0836 | | | | | | | | | | | | |  | |
|  |  | ENSGALG00000008908 | | | | | | | | | neurogenic differentiation 1 | | | | | 0.0361 | | | | | | 2.1278 | | | | | | | | | | | | |  | |
|  |  | ENSGALG00000008032 | | | | | | | | | G protein-coupled receptor 22 | | | | | 0.0374 | | | | | | 2.1532 | | | | | | | | | | | | |  | |
|  |  | ENSGALG00000015626 | | | | | | | | | regulator of G-protein signalling 12 | | | | | 0.0424 | | | | | | 1.9791 | | | | | | | | | | | | |  | |
|  |  |  | | | | | | | | |  | | | | |  | | | | | |  | | | | | | | | | | | | |  | |
|  | *(2) Top 20 down-regulated genes under 1*^st^ *class* | | | | | | | | | | | | | | |  | | | | | |  | | | | | | | | | | | | |  | |
|  |  | Ensembl ID | | | | | | | | | Description | | | | | FDR | | | | | | FC | | | | | | | | | | | | |  | |
|  |  | ENSGALG00000001063 | | | | | | | | | PR domain containing 16 | | | | | 0.0230 | | | | | | -2.0024 | | | | | | | | | | | | |  | |
|  |  | ENSGALG00000017044 | | | | | | | | | transient receptor potential cation channel, subfamily C, member 4 | | | | | 0.0258 | | | | | | -2.0181 | | | | | | | | | | | | |  | |
|  |  | ENSGALG00000006112 | | | | | | | | | sodium channel, voltage-gated,  type V, alpha subunit | | | | | 0.0327 | | | | | | -1.9275 | | | | | | | | | | | | |  | |
|  |  | ENSGALG00000009740 | | | | | | | | | similar to RAS guanyl releasing protein 1 (calcium and DAG-regulated);  RAS guanyl releasing protein 1 (calcium and DAG-regulated) | | | | | 0.0358 | | | | | | -1.8443 | | | | | | | | | | | | |  | |
|  |  | ENSGALG00000012464 | | | | | | | | | SOUL protein | | | | | 0.0445 | | | | | | -2.1554 | | | | | | | | | | | | |  | |
|  |  | ENSGALG00000013294 | | | | | | | | | cytochrome P450, family 19, subfamily A, polypeptide 1 | | | | | 0.0459 | | | | | | -2.0576 | | | | | | | | | | | | |  | |
|  |  | ENSGALG00000008621 | | | | | | | | | similar to neuropilin-2a1 receptor; neuropilin 2 | | | | | 0.0514 | | | | | | -1.6908 | | | | | | | | | | | | |  | |
|  |  | ENSGALG00000017194 | | | | | | | | | transient receptor potential cation channel, subfamily C, member 6 | | | | | 0.0522 | | | | | | -1.7468 | | | | | | | | | | | | |  | |
|  |  | ENSGALG00000014843 | | | | | | | | | tumor protein D52-like 1 | | | | | 0.0541 | | | | | | -1.8108 | | | | | | | | | | | | |  | |
|  |  | ENSGALG00000003895 | | | | | | | | | PR domain containing 12 | | | | | 0.0546 | | | | | | -1.8305 | | | | | | | | | | | | |  | |
|  |  | ENSGALG00000007047 | | | | | | | | | galanin prepropeptide | | | | | 0.0549 | | | | | | -1.9036 | | | | | | | | | | | | |  | |
|  |  | ENSGALG00000016707 | | | | | | | | | chloride intracellular channel 5 | | | | | 0.0554 | | | | | | -1.7380 | | | | | | | | | | | | |  | |
|  |  | ENSGALG00000007972 | | | | | | | | | transient receptor potential cation channel, subfamily C, member 5 | | | | | 0.0556 | | | | | | -1.7112 | | | | | | | | | | | | |  | |
|  |  | ENSGALG00000016455 | | | | | | | | | uncharacterised | | | | | 0.0560 | | | | | | -1.8658 | | | | | | | | | | | | |  | |
|  |  | ENSGALG00000014484 | | | | | | | | | uncharacterised | | | | | 0.0584 | | | | | | -1.7314 | | | | | | | | | | | | |  | |
|  |  | ENSGALG00000011369 | | | | | | | | | LIM homeobox 8 | | | | | 0.0600 | | | | | | -1.7260 | | | | | | | | | | | | |  | |
|  |  | ENSGALG00000011022 | | | | | | | | | neuropeptide VF precursor | | | | | 0.0625 | | | | | | -1.5799 | | | | | | | | | | | | |  | |
|  |  | ENSGALG00000009173 | | | | | | | | | GDNF family receptor alpha 1 | | | | | 0.0630 | | | | | | -1.6382 | | | | | | | | | | | | |  | |
|  |  | ENSGALG00000015143 | | | | | | | | | transthyretin | | | | | 0.0635 | | | | | | -2.5067 | | | | | | | | | | | | |  | |
|  |  | ENSGALG00000003149 | | | | | | | | | inositol 1,4,5-triphosphate receptor,  type 3 | | | | | 0.0660 | | | | | | -2.0682 | | | | | | | | | | | | |  | |
|  | **Contrast:**  **BB *vs* BC** (1^st^ class *vs* 2^nd^ class) | | | | | | | | | | | | | | | | | | | | | | | | | | | | | | | | | | | |
|  | *(1) Top 20 up-regulated genes under 1*^st^ *class* | | | | | | | | | | | | | | |  | | |  | | | | | | | | | | | | | | | | | |
|  |  | Ensembl ID | | | | | | | | | Description | | | | | FDR | | | | | | FC | | | | | | | | | | | | |  | |
|  |  | ENSGALG00000001564 | | | | | | | | | ATPase, Ca++ transporting, ubiquitous | | | | | <0.0001 | | | | | | 2.8468 | | | | | | | | | | | | |  | |
|  |  | ENSGALG00000004270 | | | | | | | | | aldehyde dehydrogenase 1 family, member A2 | | | | | <0.0001 | | | | | | 2.8057 | | | | | | | | | | | | |  | |
|  |  | ENSGALG00000008135 | | | | | | | | | SATB homeobox 2 | | | | | <0.0001 | | | | | | 4.0256 | | | | | | | | | | | | |  | |
|  |  | ENSGALG00000012235 | | | | | | | | | neurogenic differentiation 6 | | | | | <0.0001 | | | | | | 5.1060 | | | | | | | | | | | | |  | |
|  |  | ENSGALG00000019842 | | | | | | | | | transcription factor AP-2delta | | | | | <0.0001 | | | | | | 3.0998 | | | | | | | | | | | | |  | |
|  |  | ENSGALG00000011122 | | | | | | | | | Uncharacterised | | | | | 0.00013 | | | | | | 2.3724 | | | | | | | | | | | | |  | |
|  |  | ENSGALG00000013154 | | | | | | | | | solute carrier organic anion transporter family, member 1C1 | | | | | 0.00014 | | | | | | 2.6388 | | | | | | | | | | | | |  | |
|  |  | ENSGALG00000014907 | | | | | | | | | discoidin, CUB and LCCL domain containing 1 | | | | | 0.00017 | | | | | | 2.7727 | | | | | | | | | | | | |  | |
|  |  | ENSGALG00000023441 | | | | | | | | | reticulon 4 receptor-like 2 | | | | | 0.00018 | | | | | | 2.2530 | | | | | | | | | | | | |  | |
|  |  | ENSGALG00000012254 | | | | | | | | | potassium inwardly-rectifying channel, subfamily J, member 4 | | | | | 2.00E-04 | | | | | | 2.2675 | | | | | | | | | | | | |  | |
|  |  | ENSGALG00000007141 | | | | | | | | | leucine-rich repeat kinase 1 | | | | | 0.00022 | | | | | | 2.6386 | | | | | | | | | | | | |  | |
|  |  | ENSGALG00000003839 | | | | | | | | | glutamate receptor, metabotropic 2 | | | | | 0.00031 | | | | | | 2.3058 | | | | | | | | | | | | |  | |
|  |  | ENSGALG00000018942 | | | | | | | | | neurogranin (protein kinase C substrate, RC3) | | | | | 0.00033 | | | | | | 2.1826 | | | | | | | | | | | | |  | |
|  |  | ENSGALG00000015271 | | | | | | | | | filamin A interacting protein 1-like | | | | | 0.00071 | | | | | | 2.1682 | | | | | | | | | | | | |  | |
|  |  | ENSGALG00000014829 | | | | | | | | | R-spondin 3 homolog (Xenopus laevis) | | | | | 0.00138 | | | | | | 2.0225 | | | | | | | | | | | | |  | |
|  |  | ENSGALG00000008032 | | | | | | | | | G protein-coupled receptor 22 | | | | | 0.0014 | | | | | | 2.1012 | | | | | | | | | | | | |  | |
|  |  | ENSGALG00000014011 | | | | | | | | | lymphoid-restricted membrane protein | | | | | 0.00141 | | | | | | 2.4858 | | | | | | | | | | | | |  | |
|  |  | ENSGALG00000015626 | | | | | | | | | regulator of G-protein signalling 12 | | | | | 0.00189 | | | | | | 2.1415 | | | | | | | | | | | | |  | |
|  |  | ENSGALG00000008940 | | | | | | | | | spectrin, beta, non-erythrocytic 5 | | | | | 0.00205 | | | | | | 1.8481 | | | | | | | | | | | | |  | |
|  |  | ENSGALG00000000820 | | | | | | | | | 5-hydroxytryptamine (serotonin) receptor 1D | | | | | 0.0043 | | | | | | 1.8261 | | | | | | | | | | | | |  | |
|  | *(2) Top 20 down-regulated genes under 1*^st^ *class* | | | | | | | | | | | | | | |  | | | | | |  | | | | | | | | | | | | |  | |
|  |  | Ensembl ID | | | | | | | | | Description | | | | | FDR | | | | | | FC | | | | | | | | | | | | |  | |
|  |  | ENSGALG00000013294 | | | | | | | | | cytochrome P450, family 19, subfamily A, polypeptide 1 | | | | | 0.0060 | | | | | | -1.7109 | | | | | | | | | | | | |  | |
|  |  | ENSGALG00000004754 | | | | | | | | | obscurin, cytoskeletal calmodulin and titin-interacting RhoGEF | | | | | 0.0070 | | | | | | -1.7063 | | | | | | | | | | | | |  | |
|  |  | ENSGALG00000011369 | | | | | | | | | LIM homeobox 8 | | | | | 0.0080 | | | | | | -1.7391 | | | | | | | | | | | | |  | |
|  |  | ENSGALG00000011973 | | | | | | | | | sushi domain containing 5 | | | | | 0.0150 | | | | | | -1.7180 | | | | | | | | | | | | |  | |
|  |  | ENSGALG00000014967 | | | | | | | | | synaptic vesicle glycoprotein 2C | | | | | 0.0168 | | | | | | -1.5968 | | | | | | | | | | | | |  | |
|  |  | ENSGALG00000019144 | | | | | | | | | gamma-aminobutyric acid (GABA) A receptor, gamma 3 | | | | | 0.0275 | | | | | | -1.5698 | | | | | | | | | | | | |  | |
|  |  | ENSGALG00000006112 | | | | | | | | | sodium channel, voltage-gated, type V, alpha subunit | | | | | 0.0389 | | | | | | -1.5659 | | | | | | | | | | | | |  | |
|  |  | ENSGALG00000006473 | | | | | | | | | plexin A4, B | | | | | 0.0717 | | | | | | -1.4899 | | | | | | | | | | | | |  | |
|  |  | ENSGALG00000012495 | | | | | | | | | uncharacterised | | | | | 0.0722 | | | | | | -1.4865 | | | | | | | | | | | | |  | |
|  |  | ENSGALG00000009173 | | | | | | | | | GDNF family receptor alpha 1 | | | | | 0.0724 | | | | | | -1.5144 | | | | | | | | | | | | |  | |
|  |  | ENSGALG00000007972 | | | | | | | | | transient receptor potential cation channel,  subfamily C, member 5 | | | | | 0.0775 | | | | | | -1.5231 | | | | | | | | | | | | |  | |
|  |  | ENSGALG00000015529 | | | | | | | | | Wolfram syndrome 1 (wolframin) | | | | | 0.0778 | | | | | | -1.4117 | | | | | | | | | | | | |  | |
|  |  | ENSGALG00000014717 | | | | | | | | | uncharacterised | | | | | 0.0789 | | | | | | -1.4625 | | | | | | | | | | | | |  | |
|  |  | ENSGALG00000004838 | | | | | | | | | BUB1 budding uninhibited by benzimidazoles  1 homolog beta (yeast) | | | | | 0.0799 | | | | | | -1.5141 | | | | | | | | | | | | |  | |
|  |  | ENSGALG00000006485 | | | | | | | | | uncharacterised | | | | | 0.0836 | | | | | | -1.4800 | | | | | | | | | | | | |  | |
|  |  | ENSGALG00000012324 | | | | | | | | | chromosome 7 open reading frame 10 | | | | | 0.0859 | | | | | | -1.4490 | | | | | | | | | | | | |  | |
|  |  | ENSGALG00000013177 | | | | | | | | | branched chain aminotransferase 1, cytosolic | | | | | 0.0872 | | | | | | -1.4502 | | | | | | | | | | | | |  | |
|  |  | ENSGALG00000001608 | | | | | | | | | unc-5 homolog D (C. elegans) | | | | | 0.0952 | | | | | | -1.4394 | | | | | | | | | | | | |  | |
|  |  | ENSGALG00000016804 | | | | | | | | | solute carrier family 5 (choline transporter), member 7 | | | | | 0.0979 | | | | | | -1.4437 | | | | | | | | | | | | |  | |
|  | **Contrast:**  **BB *vs* CB** (1^st^ class *vs* 2^nd^ class) | | | | | | | | | | | | | | | | | | | | | | | | | | | | | | | | | | | |
|  | *(1) Top 20 up-regulated genes under 1*^st^ *class* | | | | | | | | | | | | | | | | | | | | | | | | | | | |  | | | | | | | |
|  |  | Ensembl ID | | | | | | | | | | Description | | | | | | FDR | | | | FC | | | | | | | | | | | | |  | |
|  |  | ENSGALG00000015143 | | | | | | | | | | transthyretin | | | | | | 0.0020 | | | | 7.8399 | | | | | | | | | | | | |  | |
|  |  | ENSGALG00000001696 | | | | | | | | | | S-antigen; retina and pineal gland (arrestin) | | | | | | 0.0200 | | | | 3.2449 | | | | | | | | | | | | |  | |
|  |  | ENSGALG00000014634 | | | | | | | | | | silver homolog (mouse) | | | | | | 0.0300 | | | | 2.3372 | | | | | | | | | | | | |  | |
|  |  | ENSGALG00000016020 | | | | | | | | | | chloride intracellular channel 6 | | | | | | 0.0610 | | | | 1.9277 | | | | | | | | | | | | |  | |
|  |  | ENSGALG00000001063 | | | | | | | | | | PR domain containing 16 | | | | | | 0.0686 | | | | 1.7119 | | | | | | | | | | | | |  | |
|  |  | ENSGALG00000007179 | | | | | | | | | | ATPase type 13A5 | | | | | | 0.0688 | | | | 1.9963 | | | | | | | | | | | | |  | |
|  |  | ENSGALG00000011859 | | | | | | | | | | eye-globin | | | | | | 0.0710 | | | | 1.9526 | | | | | | | | | | | | |  | |
|  |  | ENSGALG00000008941 | | | | | | | | | | uncharacterised | | | | | | 0.0711 | | | | 1.6936 | | | | | | | | | | | | |  | |
|  |  | ENSGALG00000013154 | | | | | | | | | | solute carrier organic anion transporter family, member 1C1 | | | | | | 0.0855 | | | | 2.4170 | | | | | | | | | | | | |  | |
|  |  | ENSGALG00000009867 | | | | | | | | | | WNT inhibitory factor 1 | | | | | | 0.0947 | | | | 1.7129 | | | | | | | | | | | | |  | |
|  | *(2) Top 20 down-regulated genes under 1*^st^ *class* | | | | | | | | | | | | | | | | |  | | | |  | | | | | | | | | | | | |  | |
|  |  | ENSGALG00000014118 | | | | | | | | | | mitochondrial ribosomal protein S26 | | | | | | 0.0000 | | | | -2.2201 | | | | | | | | | | | | |  | |
|  |  | ENSGALG00000018808 | | | | | | | | | | mitochondrial ribosomal protein S26 | | | | | | 0.0075 | | | | -1.9027 | | | | | | | | | | | | |  | |
|  |  | ENSGALG00000002821 | | | | | | | | | | gastrin-releasing peptide | | | | | | 0.0423 | | | | -1.7801 | | | | | | | | | | | | |  | |

**Table S5.** List of down- and up-regulated genes (highlighted in green and red, respectively; Ensembl IDs are in ascending order) that met the behavioural filtering categories in the (a) hippocampus and (b) hypothalamus.

**(a) Hippocampus**

| *Pre- and post-natal B responsive genes (behavioural category I)* | | |
| --- | --- | --- |
|  | Ensembl ID | Description |
|  | ENSGALG00000000713 | zinc finger homeobox 3 |
|  | ENSGALG00000000745 | solute carrier family 26, member 9 |
|  | ENSGALG00000001115 | membrane metallo-endopeptidase-like 1 |
|  | ENSGALG00000001490 | Potassium inwardly-rectifyin channel, subfamily J, member 13 |
|  | ENSGALG00000002041 | agrin |
|  | ENSGALG00000002757 | Uncharacterised |
|  | ENSGALG00000002854 | PDZ domain containing 2 |
|  | ENSGALG00000003115 | Uncharacterised |
|  | ENSGALG00000003473 | secreted frizzled-related protein 1 |
|  | ENSGALG00000004322 | Uncharacterised |
|  | ENSGALG00000004414 | leucine zipper protein 2 |
|  | ENSGALG00000004630 | similar to cHz-cadherin |
|  | ENSGALG00000004814 | rhophilin, Rho GTPase binding protein 2 |
|  | ENSGALG00000005259 | vasoactive intestinal peptide receptor 1 |
|  | ENSGALG00000005956 | annexin A8-like 1 |
|  | ENSGALG00000006269 | Uncharacterised |
|  | ENSGALG00000006306 | urocanase domain containing 1 |
|  | ENSGALG00000006313 | interleukin 4 receptor |
|  | ENSGALG00000007211 | cadherin-like 22 |
|  | ENSGALG00000007226 | osteocrin |
|  | ENSGALG00000007367 | WAP, follistatin/kazal, immunoglobulin, kunitz and netrin domain containing 2 |
|  | ENSGALG00000007410 | similar to hDDM36 |
|  | ENSGALG00000007487 | chromosome 21 open reading frame 58 |
|  | ENSGALG00000007596 | hypothetical LOC416086 |
|  | ENSGALG00000008150 | RAS protein activator like 1 (GAP1 like) |
|  | ENSGALG00000008263 | contactin 4 |
|  | ENSGALG00000008874 | solute carrier family 13 (sodium/sulfate symporters), member 1 |
|  | ENSGALG00000008926 | Ca2+-dependent activator protein for secretion 2 |
|  | ENSGALG00000008980 | von Willebrand factor A domain containing 2 |
|  | ENSGALG00000009006 | six transmembrane epithelial antigen of the prostate 1 |
|  | ENSGALG00000009315 | Uncharacterised |
|  | ENSGALG00000009497 | arginine vasopressin receptor 2 (nephrogenic diabetes insipidus) |
|  | ENSGALG00000009799 | Meis homeobox 2 |
|  | ENSGALG00000010035 | nuclear receptor subfamily 3, group C, member 2 |
|  | ENSGALG00000011717 | hypothetical LOC417937 |
|  | ENSGALG00000011813 | HEG homolog 1 (zebrafish) |
|  | ENSGALG00000011836 | solute carrier family 6 (proline IMINO transporter), member 20 |
|  | ENSGALG00000011858 | potassium voltage-gated channel, subfamily H (eag-related), member 5 |
|  | ENSGALG00000012163 | brain-derived neurotrophic factor |
|  | ENSGALG00000012183 | neuronal pentraxin receptor |
|  | ENSGALG00000012235 | neurogenic differentiation 6 |
|  | ENSGALG00000012421 | Rho GTPase activating protein 15 |
|  | ENSGALG00000012568 | TIMP metallopeptidase inhibitor 3 |
|  | ENSGALG00000013154 | solute carrier organic anion transporter family, member 1C1 |
|  | ENSGALG00000014414 | gamma-aminobutyric acid (GABA) receptor, rho 3 |
|  | ENSGALG00000014978 | IQ motif containing GTPase activating protein 2 |
|  | ENSGALG00000015205 | tyrosinase-related protein 1 |
|  | ENSGALG00000015720 | chondrolectin |
|  | ENSGALG00000016411 | similar to collagen XIV; collagen, type XIV, alpha 1 (undulin); similar to collagen, type XIV, alpha 1 (undulin) |
|  | ENSGALG00000016616 | similar to Kallmann syndrome gene product; |
|  | ENSGALG00000016884 | solute carrier family 15 (oligopeptide transporter), member 1 |
|  | ENSGALG00000017343 | folate receptor 1 (adult) |
|  | ENSGALG00000023580 | Uncharacterised |
| *Interacting pre- and post-natal B responsive genes: “cumulative effect” (behavioral category II)* | | |
|  | Ensembl ID | Description |
|  | ENSGALG00000000733 | myosin VIIA |
|  | ENSGALG00000001006 | tumor protein p73 |
|  | ENSGALG00000001063 | PR domain containing 16 |
|  | ENSGALG00000004879 | solute carrier family 6 (neurotransmitter transporter, GABA), member 11 |
|  | ENSGALG00000005400 | calcium channel, voltage-dependent, alpha 2/delta 3 subunit |
|  | ENSGALG00000006413 | KIAA1199 |
|  | ENSGALG00000006449 | glutamate receptor interacting protein 2 |
|  | ENSGALG00000008445 | solute carrier family 24 (sodium/potassium/calcium exchanger), member 3 |
|  | ENSGALG00000009034 | anaplastic lymphoma kinase (Ki-1) |
|  | ENSGALG00000010781 | glycine receptor, alpha 3 |
|  | ENSGALG00000010858 | low density lipoprotein-related protein 2 |
|  | ENSGALG00000012917 | cadherin 6, type 2, K-cadherin (fetal kidney); similar to CDH6 protein |
|  | ENSGALG00000013953 | tachykinin receptor 1 |
|  | ENSGALG00000015673 | zinc finger homeodomain 4 |
|  | ENSGALG00000015857 | carbonic anhydrase III, muscle specific |
|  | ENSGALG00000015865 | similar to C6orf37 |
|  | ENSGALG00000016017 | solute carrier family 4, sodium borate transporter, member 11 |
|  | ENSGALG00000016866 | fibroblast growth factor 14 |
|  | ENSGALG00000017021 | ATPase, Cu++ transporting, beta polypeptide |
|  | ENSGALG00000017040 | Uncharacterised |
|  | ENSGALG00000017068 | klotho |
|  | ENSGALG00000017405 | Uncharacterised |
|  | ENSGALG00000021039 | hexokinase domain containing 1 |
|  | ENSGALG00000023552 | Uncharacterised |
| *Specific pre-natal B responsive genes (behavioural category III)* | | |
|  | Ensembl ID | Description |
|  | ENSGALG00000001396 | serpin peptidase inhibitor, clade D (heparin cofactor), member 1 |
|  | ENSGALG00000001696 | S-antigen; retina and pineal gland (arrestin) |
|  | ENSGALG00000003842 | growth hormone releasing hormone |
|  | ENSGALG00000003895 | PR domain containing 12 |
|  | ENSGALG00000007025 | copine VIII |
|  | ENSGALG00000007588 | glutamate decarboxylase 2 (pancreatic islets and brain, 65kDa) |
|  | ENSGALG00000007908 | EGF-containing fibulin-like extracellular matrix protein 1 |
|  | ENSGALG00000008188 | tripartite motif-containing 36 |
|  | ENSGALG00000008883 | transcription factor 7-like 2 (T-cell specific, HMG-box) |
|  | ENSGALG00000009129 | distal-less homeobox 5 |
|  | ENSGALG00000009739 | adhesion molecule with Ig-like domain 2 |
|  | ENSGALG00000009740 | similar to RAS guanyl releasing protein 1 (calcium and DAG-regulated); RAS guanyl releasing protein 1 (calcium and DAG-regulated) |
|  | ENSGALG00000010865 | transmembrane protein 196 |
|  | ENSGALG00000012907 | melanocortin 4 receptor |
|  | ENSGALG00000012911 | synaptotagmin X |
|  | ENSGALG00000013294 | cytochrome P450, family 19, subfamily A, polypeptide 1 |
|  | ENSGALG00000013890 | melanocortin 5 receptor |
|  | ENSGALG00000014233 | fibulin 1 |
|  | ENSGALG00000015143 | transthyretin |
|  | ENSGALG00000015419 | proenkephalin |
|  | ENSGALG00000016035 | GFR receptor alpha 4; similar to GFR receptor alpha 4 |
|  | ENSGALG00000016324 | glutathione S-transferase alpha 3 |
|  | ENSGALG00000017418 | neuronal pentraxin I |
|  | ENSGALG00000019277 | solute carrier organic anion transporter family, member 1B3 |
|  | ENSGALG00000020381 | deiodinase, iodothyronine, type III |
|  | ENSGALG00000022819 | Purkinje cell protein 4 |

**(b) Hypothalamus**

| *Pre- and post-natal B responsive genes (behavioural category I)* | | |
| --- | --- | --- |
|  | Ensembl ID | Description |
|  | ENSGALG00000001136 | similar to enhancer of split related protein-7 |
|  | ENSGALG00000001896 | netrin G1 |
|  | ENSGALG00000002331 | calbindin 2, 29kDa (calretinin) |
|  | ENSGALG00000005526 | hairy and enhancer of split 6 (Drosophila) |
|  | ENSGALG00000006271 | Rac GTPase activating protein 1 |
|  | ENSGALG00000006838 | similar to iron binding protein |
|  | ENSGALG00000009058 | ectonucleoside triphosphate diphosphohydrolase 2 |
|  | ENSGALG00000009861 | retinal degeneration 3 |
|  | ENSGALG00000010065 | potassium channel, subfamily K, member 5 |
|  | ENSGALG00000010461 | early B-cell factor 3 |
|  | ENSGALG00000010583 | vitrin |
|  | ENSGALG00000011066 | calmin (calponin-like, transmembrane) |
|  | ENSGALG00000011127 | B-cell CLL/lymphoma 11B (zinc finger protein) |
|  | ENSGALG00000011940 | cholecystokinin |
|  | ENSGALG00000012732 | phosphatase and actin regulator 1 |
| *Interacting pre- and post-natal B responsive genes: “cumulative effect” (behavioural category II)* | | |
|  | Ensembl ID | Description |
|  | ENSGALG00000011883 | C-type lectin domain family 3 |
|  | ENSGALG00000022217 | similar to protocadherin gamma C5 |
|  | ENSGALG00000023036 | Uncharacterised |

| *Specific pre-natal B responsive genes (behavioural category III)* | | |
| --- | --- | --- |
|  | Ensembl ID | Description |
|  | ENSGALG00000001063 | PR domain containing 16 |
|  | ENSGALG00000003149 | inositol 1,4,5-triphosphate receptor, type 3 |
|  | ENSGALG00000003895 | PR domain containing 12 |
|  | ENSGALG00000004860 | RAS, dexamethasone-induced 1 |
|  | ENSGALG00000006014 | protein kinase C, beta |
|  | ENSGALG00000009095 | luteinizing hormone/choriogonadotropin receptor |
|  | ENSGALG00000010402 | prostaglandin-D synthase |
|  | ENSGALG00000013193 | iroquois homeobox 2 |
|  | ENSGALG00000014843 | tumor protein D52-like 1 |
|  | ENSGALG00000015824 | glycoprotein hormones, alpha polypeptide |
|  | ENSGALG00000015857 | carbonic anhydrase III, muscle specific |
|  | ENSGALG00000017044 | transient receptor potential cation channel, subfamily C, member 4 |
| *Specific post-natal B responsive genes (behavioural category IV)* | | |
|  | Ensembl ID | Description |
|  | ENSGALG00000000098 | anthrax toxin receptor 1 |
|  | ENSGALG00000000168 | adenosine A1 receptor |
|  | ENSGALG00000000376 | Uncharacterised |
|  | ENSGALG00000000695 | major facilitator superfamily domain containing 4 |
|  | ENSGALG00000000820 | 5-hydroxytryptamine (serotonin) receptor 1D |
|  | ENSGALG00000001227 | Uncharacterised |
|  | ENSGALG00000001505 | neuronal guanine nucleotide exchange factor |
|  | ENSGALG00000002470 | cytochrome P450, family 27, subfamily A, polypeptide 1 |
|  | ENSGALG00000003285 | protocadherin 24 |
|  | ENSGALG00000003437 | ADAM metallopeptidase domain 8 |
|  | ENSGALG00000003670 | v-maf musculoaponeurotic fibrosarcoma oncogene homolog B |
|  | ENSGALG00000004074 | potassium voltage-gated channel, delayed-rectifier, subfamily S, member 1 |
|  | ENSGALG00000004270 | aldehyde dehydrogenase 1 family, member A2 |
|  | ENSGALG00000005258 | somatostatin receptor 5 |
|  | ENSGALG00000005657 | corticotropin releasing hormone receptor 2 |
|  | ENSGALG00000005853 | 5-hydroxytryptamine (serotonin) receptor 2C |
|  | ENSGALG00000006021 | calcium channel, voltage-dependent, gamma subunit 3 |
|  | ENSGALG00000006439 | Rac/Cdc42 guanine nucleotide exchange factor (GEF) 6 |
|  | ENSGALG00000006886 | dachshund homolog 2 (Drosophila) |
|  | ENSGALG00000007004 | 5-hydroxytryptamine (serotonin) receptor 3A |
|  | ENSGALG00000007141 | leucine-rich repeat kinase 1 |
|  | ENSGALG00000007278 | glutamate receptor, ionotropic, N-methyl D-aspartate 2A |
|  | ENSGALG00000007349 | RAS-like, family 12 |
|  | ENSGALG00000007415 | SH3 domain containing ring finger 2 |
|  | ENSGALG00000008032 | G protein-coupled receptor 22 |
|  | ENSGALG00000008135 | SATB homeobox 2 |
|  | ENSGALG00000008308 | basic helix-loop-helix domain containing, class B, 2 |
|  | ENSGALG00000008631 | TYRO3 protein tyrosine kinase |
|  | ENSGALG00000008671 | ST8 alpha-N-acetyl-neuraminide alpha-2,8-sialyltransferase 6 |
|  | ENSGALG00000008940 | spectrin, beta, non-erythrocytic 5 |
|  | ENSGALG00000009859 | TBC1 domain family, member 30 |
|  | ENSGALG00000010705 | zinc finger protein 238 |
|  | ENSGALG00000011122 | Uncharacterised |
|  | ENSGALG00000011254 | SATB homeobox 1 |
|  | ENSGALG00000011406 | netrin 4 |
|  | ENSGALG00000011592 | muscle RAS oncogene homolog |
|  | ENSGALG00000012046 | similar to ARPP-21 protein |
|  | ENSGALG00000012054 | doublecortin-like kinase 3 |
|  | ENSGALG00000012154 | F-box protein 34 |
|  | ENSGALG00000012235 | neurogenic differentiation 6 |
|  | ENSGALG00000012254 | potassium inwardly-rectifying channel, subfamily J, member 4 |
|  | ENSGALG00000012322 | potassium channel tetramerisation domain containing 16 |
|  | ENSGALG00000012367 | tripartite motif-containing 9 |
|  | ENSGALG00000012542 | RASD family, member 2 |
|  | ENSGALG00000012890 | diacylglycerol kinase, iota |
|  | ENSGALG00000013051 | sema domain, seven thrombospondin repeats (type 1 and type 1-like), transmembrane domain (TM) and short cytoplasmic domain, (semaphorin) 5A |
|  | ENSGALG00000013948 | RAS-like, family 11, member B |
|  | ENSGALG00000014011 | lymphoid-restricted membrane protein |
|  | ENSGALG00000014186 | metallophosphoesterase domain containing 1 |
|  | ENSGALG00000014812 | SID1 transmembrane family, member 1 |
|  | ENSGALG00000014907 | discoidin, CUB and LCCL domain containing 1 |
|  | ENSGALG00000015271 | filamin A interacting protein 1-like |
|  | ENSGALG00000015403 | EPH receptor A3 |
|  | ENSGALG00000015626 | regulator of G-protein signalling 12 |
|  | ENSGALG00000015970 | collagen, type IX, alpha 1 |
|  | ENSGALG00000016084 | R-spondin 2 homolog (Xenopus laevis) |
|  | ENSGALG00000016095 | empty spiracles homeobox 1 |
|  | ENSGALG00000016391 | connector enhancer of kinase suppressor of Ras 2 |
|  | ENSGALG00000016396 | collectin sub-family member 11 |
|  | ENSGALG00000016744 | gamma-aminobutyric acid (GABA) A receptor, alpha 5 |
|  | ENSGALG00000016843 | collagen, type IV, alpha 2 |
|  | ENSGALG00000016920 | LIM domain 7 |
|  | ENSGALG00000016944 | protocadherin 8 |
|  | ENSGALG00000016983 | Uncharacterised |
|  | ENSGALG00000017378 | cartilage acidic protein 1 |
|  | ENSGALG00000017690 | potassium voltage-gated channel, delayed-rectifier, subfamily S, member 2 |
|  | ENSGALG00000019842 | transcription factor AP-2delta |
|  | ENSGALG00000020975 | transmembrane protein 233 |
|  | ENSGALG00000022782 | Uncharacterised |
|  | ENSGALG00000022988 | Uncharacterised |
|  | ENSGALG00000023881 | plexin domain containing 1 |

**Table S6** Lists of down- and up-regulated genes (highlighted in green and red, respectively) in the (a) hippocampus and (b) hypothalamus submitted to Ingenuity Pathway Analysis (IPA) after filtering the Vector Analysis data according to the behavioural categories. The genes in black are the non-redundant “focus” genes with records in the IPA server, whilst in blue are the “non-focus” genes.

| **(a) Hippocampus** | | | |  |  |
| --- | --- | --- | --- | --- | --- |
| *Pre- and post-natal B responsive genes and cumulative responses to the B treatment (behavioural category I and II grouped together)* | | | | | |
|  | Ensembl ID | Symbol | Description | | |
|  | ENSGALG00000000713 | ZFHX3 | zinc finger homeobox 3 | | |
|  | ENSGALG00000000733 | MYO7A | myosin VIIA | | |
|  | ENSGALG00000000745 | SLC26A9 | solute carrier family 26, member 9 | | |
|  | ENSGALG00000001006 | TP73 | tumor protein p73 | | |
|  | ENSGALG00000001063 | PRDM16 | PR domain containing 16 | | |
|  | ENSGALG00000001115 | MMEL1 | membrane metallo-endopeptidase-like 1 | | |
|  | ENSGALG00000001490 | KCNJ13 | Potassium inwardly-rectifying channel, subfamily J, member 13 | | |
|  | ENSGALG00000002041 | AGRN | agrin | | |
|  | ENSGALG00000002854 | PDZD2 | PDZ domain containing 2 | | |
|  | ENSGALG00000003115 | COL4A3 | collagen, type IV, alpha 3 (Goodpasture antigen) | | |
|  | ENSGALG00000003473 | SFRP1 | secreted frizzled-related protein 1 | | |
|  | ENSGALG00000004814 | RHPN2 | rhophilin, Rho GTPase binding protein 2 | | |
|  | ENSGALG00000004879 | SLC6A11 | solute carrier family 6 (neurotransmitter transporter, GABA), member 11 | | |
|  | ENSGALG00000005259 | VIPR1 | vasoactive intestinal peptide receptor 1 | | |
|  | ENSGALG00000005400 | CACNA2D3 | calcium channel, voltage-dependent, alpha 2/delta subunit 3 | | |
|  | ENSGALG00000006306 | UROC1 | urocanate hydratase 1 | | |
|  | ENSGALG00000006413 | KIAA1199 | KIAA1199 | | |
|  | ENSGALG00000006449 | GRIP2 | glutamate receptor interacting protein 2 | | |
|  | ENSGALG00000007211 | CDH22 | cadherin 22, type 2 | | |
|  | ENSGALG00000007226 | OSTN | osteocrin | | |
|  | ENSGALG00000007367 | WFIKKN2 | WAP, follistatin/kazal, immunoglobulin, kunitz and netrin domain containing 2 | | |
|  | ENSGALG00000007410 | IGDCC4 | immunoglobulin superfamily, DCC subclass, member 4 | | |
|  | ENSGALG00000007487 | C21orf58 | chromosome 21 open reading frame 58 | | |
|  | ENSGALG00000008263 | CNTN4 | contactin 4 | | |
|  | ENSGALG00000008445 | SLC24A3 | solute carrier family 24 (sodium/potassium/calcium exchanger), member 3 | | |
|  | ENSGALG00000008874 | SLC13A1 | solute carrier family 13 (sodium/sulfate symporters), member 1 | | |
|  | ENSGALG00000008980 | VWA2 | von Willebrand factor A domain containing 2 | | |
|  | ENSGALG00000009006 | STEAP1 | six transmembrane epithelial antigen of the prostate 1 | | |
|  | ENSGALG00000009034 | ALK | anaplastic lymphoma receptor tyrosine kinase | | |
|  | ENSGALG00000009799 | MEIS2 | Meis homeobox 2 | | |
|  | ENSGALG00000010781 | GLRA3 | glycine receptor, alpha 3 | | |
|  | ENSGALG00000010858 | LRP2 | low density lipoprotein receptor-related protein 2 | | |
|  | ENSGALG00000011717 | 2010107G12Rik | RIKEN cDNA 2010107G12 gene | | |
|  | ENSGALG00000011836 | SLC6A20 | solute carrier family 6 (proline IMINO transporter), member 20 | | |
|  | ENSGALG00000011858 | KCNH5 | potassium voltage-gated channel, subfamily H (eag-related), member 5 | | |
|  | ENSGALG00000012568 | TIMP3 | TIMP metallopeptidase inhibitor 3 | | |
|  | ENSGALG00000012917 | CDH6 | cadherin 6, type 2, K-cadherin (fetal kidney) | | |
|  | ENSGALG00000013154 | SLCO1C1 | solute carrier organic anion transporter family, member 1C1 | | |
|  | ENSGALG00000013953 | TACR1 | tachykinin receptor 1 | | |
|  | ENSGALG00000014978 | IQGAP2 | IQ motif containing GTPase activating protein 2 | | |
|  | ENSGALG00000015205 | TYRP1 | tyrosinase-related protein 1 | | |
|  | ENSGALG00000015673 | ZFHX4 | zinc finger homeobox 4 | | |
|  | ENSGALG00000015865 | FAM46A | family with sequence similarity 46, member A | | |
|  | ENSGALG00000016017 | SLC4A11 | solute carrier family 4, sodium borate transporter, member 11 | | |
|  | ENSGALG00000016411 | COL14A1 | collagen, type XIV, alpha 1 | | |
|  | ENSGALG00000016616 | KAL1 | Kallmann syndrome 1 sequence | | |
|  | ENSGALG00000016884 | SLC15A1 | solute carrier family 15 (oligopeptide transporter), member 1 | | |
|  | ENSGALG00000017021 | ATP7B | ATPase, Cu++ transporting, beta polypeptide | | |
|  | ENSGALG00000017068 | KL | klotho | | |
|  | ENSGALG00000017343 | FOLR1 | folate receptor 1 (adult) | | |
|  | ENSGALG00000017405 | NPR3 | natriuretic peptide receptor C/guanylate cyclase C (atrionatriuretic peptide receptor C) | | |
|  | ENSGALG00000021039 | HKDC1 | hexokinase domain containing 1 | | |
|  | ENSGALG00000023580 | CLDN19 | claudin 19 | | |
|  | ENSGALG00000004414 | LUZP2 | leucine zipper protein 2 | | |
|  | ENSGALG00000008926 | CADPS2 | Ca++-dependent secretion activator 2 | | |
|  | ENSGALG00000010035 | NR3C2 | nuclear receptor subfamily 3, group C, member 2 | | |
|  | ENSGALG00000012163 | BDNF | brain-derived neurotrophic factor | | |
|  | ENSGALG00000012235 | NEUROD6 | neuronal differentiation 6 | | |
|  | ENSGALG00000012421 | ARHGAP15 | Rho GTPase activating protein 15 | | |
|  | ENSGALG00000014414 | Gabrr3 | gamma-aminobutyric acid (GABA) receptor, rho 3 | | |
|  | ENSGALG00000015720 | CHODL | Chondrolectin | | |
|  | ENSGALG00000015857 | CA3 | carbonic anhydrase III, muscle specific | | |

**(b) Hypothalamus**

*Specific post-natal B responsive genes (behavioural category IV)*

|  | Ensembl ID | Symbol | | Description |
| --- | --- | --- | --- | --- |
|  | ENSGALG00000008671 | ST8SIA6 | ST8 alpha-N-acetyl-neuraminide alpha-2,8-sialyltransferase 6 | |
|  | ENSGALG00000013051 | SEMA5A | sema domain, seven thrombospondin repeats (type 1 and type 1-like), transmembrane domain (TM) and short cytoplasmic domain, (semaphorin) 5A | |
|  | ENSGALG00000000098 | ANTXR1 | anthrax toxin receptor 1 | |
|  | ENSGALG00000000168 | ADORA1 | adenosine A1 receptor | |
|  | ENSGALG00000000695 | MFSD4 | major facilitator superfamily domain containing 4 | |
|  | ENSGALG00000000820 | HTR1D | 5-hydroxytryptamine (serotonin) receptor 1D, G protein-coupled | |
|  | ENSGALG00000001227 | PIK3R6 | phosphoinositide-3-kinase, regulatory subunit 6 | |
|  | ENSGALG00000001505 | NGEF | neuronal guanine nucleotide exchange factor | |
|  | ENSGALG00000002470 | CYP27A1 | cytochrome P450, family 27, subfamily A, polypeptide 1 | |
|  | ENSGALG00000003285 | CDHR2 | cadherin-related family member 2 | |
|  | ENSGALG00000003437 | ADAM8 | ADAM metallopeptidase domain 8 | |
|  | ENSGALG00000004074 | KCNS1 | potassium voltage-gated channel, delayed-rectifier, subfamily S, member 1 | |
|  | ENSGALG00000004270 | ALDH1A2 | aldehyde dehydrogenase 1 family, member A2 | |
|  | ENSGALG00000005258 | SSTR5 | somatostatin receptor 5 | |
|  | ENSGALG00000005657 | CRHR2 | corticotropin releasing hormone receptor 2 | |
|  | ENSGALG00000006439 | ARHGEF6 | Rac/Cdc42 guanine nucleotide exchange factor (GEF) 6 | |
|  | ENSGALG00000006886 | DACH2 | dachshund homolog 2 (Drosophila) | |
|  | ENSGALG00000007004 | HTR3A | 5-hydroxytryptamine (serotonin) receptor 3A, ionotropic | |
|  | ENSGALG00000007141 | LRRK1 | leucine-rich repeat kinase 1 | |
|  | ENSGALG00000007278 | GRIN2A | glutamate receptor, ionotropic, N-methyl D-aspartate 2A | |
|  | ENSGALG00000007349 | RASL12 | RAS-like, family 12 | |
|  | ENSGALG00000007415 | SH3RF2 | SH3 domain containing ring finger 2 | |
|  | ENSGALG00000008032 | GPR22 | G protein-coupled receptor 22 | |
|  | ENSGALG00000008135 | SATB2 | SATB homeobox 2 | |
|  | ENSGALG00000008308 | BHLHE40 | basic helix-loop-helix family, member e40 | |
|  | ENSGALG00000008631 | TYRO3 | TYRO3 protein tyrosine kinase | |
|  | ENSGALG00000008940 | SPTBN5 | spectrin, beta, non-erythrocytic 5 | |
|  | ENSGALG00000009859 | TBC1D30 | TBC1 domain family, member 30 | |
|  | ENSGALG00000010705 | ZBTB18 | zinc finger and BTB domain containing 18 | |
|  | ENSGALG00000011254 | SATB1 | SATB homeobox 1 | |
|  | ENSGALG00000011406 | NTN4 | netrin 4 | |
|  | ENSGALG00000011592 | MRAS | muscle RAS oncogene homolog | |
|  | ENSGALG00000012046 | ARPP21 | cAMP-regulated phosphoprotein, 21kDa | |
|  | ENSGALG00000012054 | DCLK3 | doublecortin-like kinase 3 | |
|  | ENSGALG00000012154 | FBXO34 | F-box protein 34 | |
|  | ENSGALG00000012235 | NEUROD6 | neuronal differentiation 6 | |
|  | ENSGALG00000012254 | KCNJ4 | potassium inwardly-rectifying channel, subfamily J, member 4 | |
|  | ENSGALG00000012322 | KCTD16 | potassium channel tetramerisation domain containing 16 | |
|  | ENSGALG00000012542 | RASD2 | RASD family, member 2 | |
|  | ENSGALG00000012890 | DGKI | diacylglycerol kinase, iota | |
|  | ENSGALG00000013948 | RASL11B | RAS-like, family 11, member B | |
|  | ENSGALG00000014011 | LRMP | lymphoid-restricted membrane protein | |
|  | ENSGALG00000014186 | MPPED1 | metallophosphoesterase domain containing 1 | |
|  | ENSGALG00000014812 | SIDT1 | SID1 transmembrane family, member 1 | |
|  | ENSGALG00000014907 | DCBLD1 | discoidin, CUB and LCCL domain containing 1 | |
|  | ENSGALG00000015271 | FILIP1L | filamin A interacting protein 1-like | |
|  | ENSGALG00000015403 | EPHA3 | EPH receptor A3 | |
|  | ENSGALG00000015626 | RGS12 | regulator of G-protein signaling 12 | |
|  | ENSGALG00000015970 | COL9A1 | collagen, type IX, alpha 1 | |
|  | ENSGALG00000016095 | EMX1 | empty spiracles homeobox 1 | |
|  | ENSGALG00000016391 | CNKSR2 | connector enhancer of kinase suppressor of Ras 2 | |
|  | ENSGALG00000016744 | GABRA5 | gamma-aminobutyric acid (GABA) A receptor, alpha 5 | |
|  | ENSGALG00000016843 | COL4A2 | collagen, type IV, alpha 2 | |
|  | ENSGALG00000016920 | LMO7 | LIM domain 7 | |
|  | ENSGALG00000016944 | PCDH8 | protocadherin 8 | |
|  | ENSGALG00000017378 | CRTAC1 | cartilage acidic protein 1 | |
|  | ENSGALG00000017690 | KCNS2 | potassium voltage-gated channel, delayed-rectifier, subfamily S, member 2 | |
|  | ENSGALG00000019842 | TFAP2D | transcription factor AP-2delta | |
|  | ENSGALG00000020975 | TMEM233 | transmembrane protein 233 | |
|  | ENSGALG00000023881 | PLXDC1 | plexin domain containing 1 | |


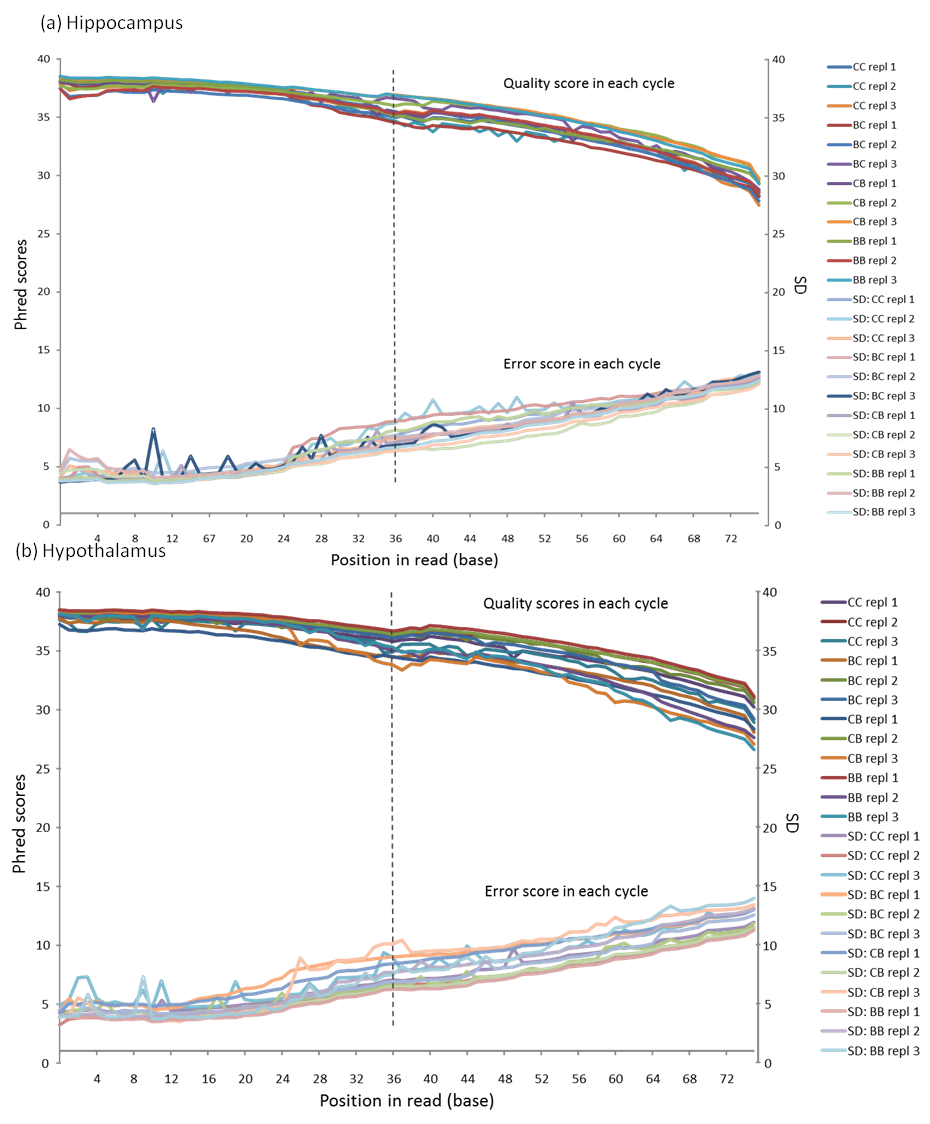


**Fig. S1.** Average Illumina quality Phred scores and their corresponding standard deviations (i.e. “SD” in the legend, estimate of the error score) in each cycle per base-call along the 76 base reads in the (a) hippocampus (n = 12) and (b) hypothalamus (n = 12) across the three pooled biological replicates (repl 1, repl 2 and repl 3) in each experimental treatment group (CC, BC, CB, or BB). The values were calculated using our *in-house* script (by P.H.). The dotted line at the 36^th^ base represents the length chosen to trim the reads that were then used in the further analyses.

**
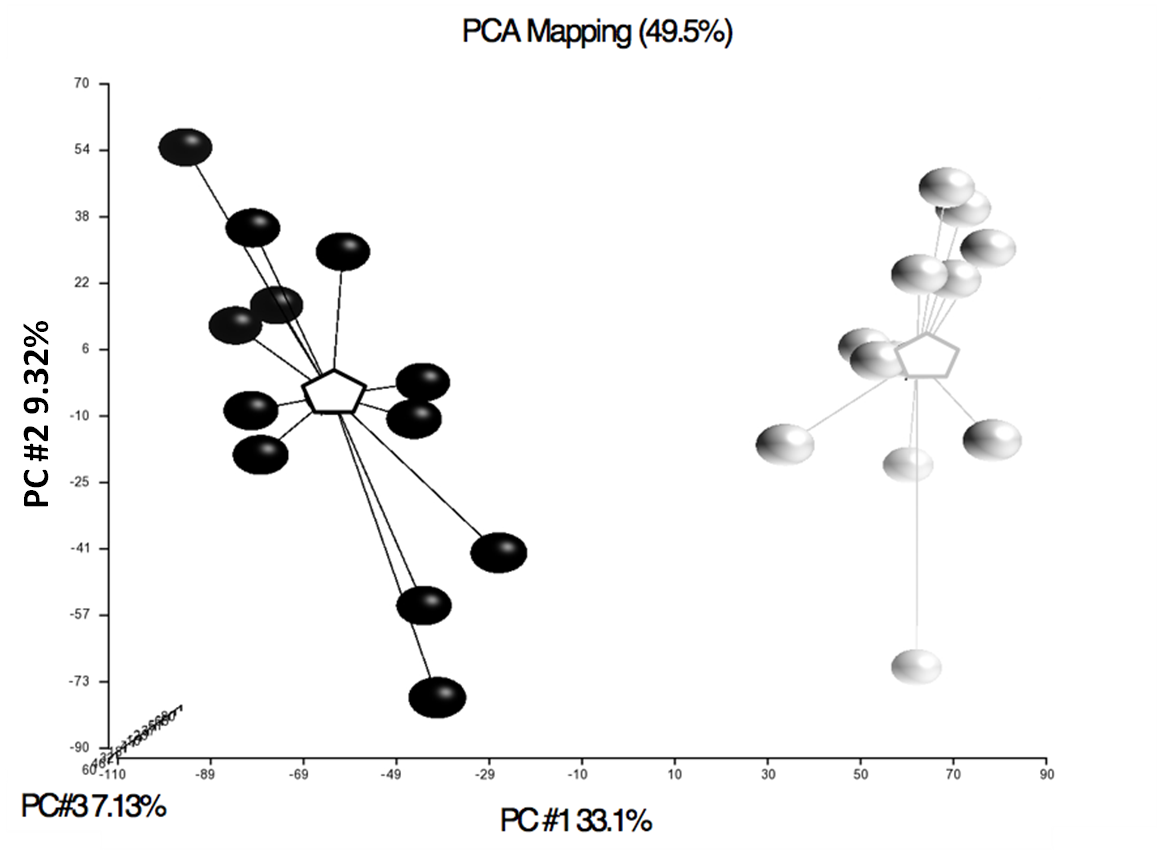
**

**Fig. S2.** Principal Component Analysis (PCA) plot of all RNA-seq sample (n = 24) using the RNA-seq normalized counts [log2 (normalized count + 32)]. Data are clustered by tissue type using the centroid function, regardless of the treatment groups. Filled black circles: hippocampal samples; filled grey circles: hypothalamic samples. PC #1 = first component, explaining 33.1% of the variation across genes; PC #2 = second component, explaining 9.32% of the variation across genes, and PC #3 = third component, explaining 7.13% of the variation across genes. The cluster centroids (represented by pentagons) are theoretical and do not represent real samples.


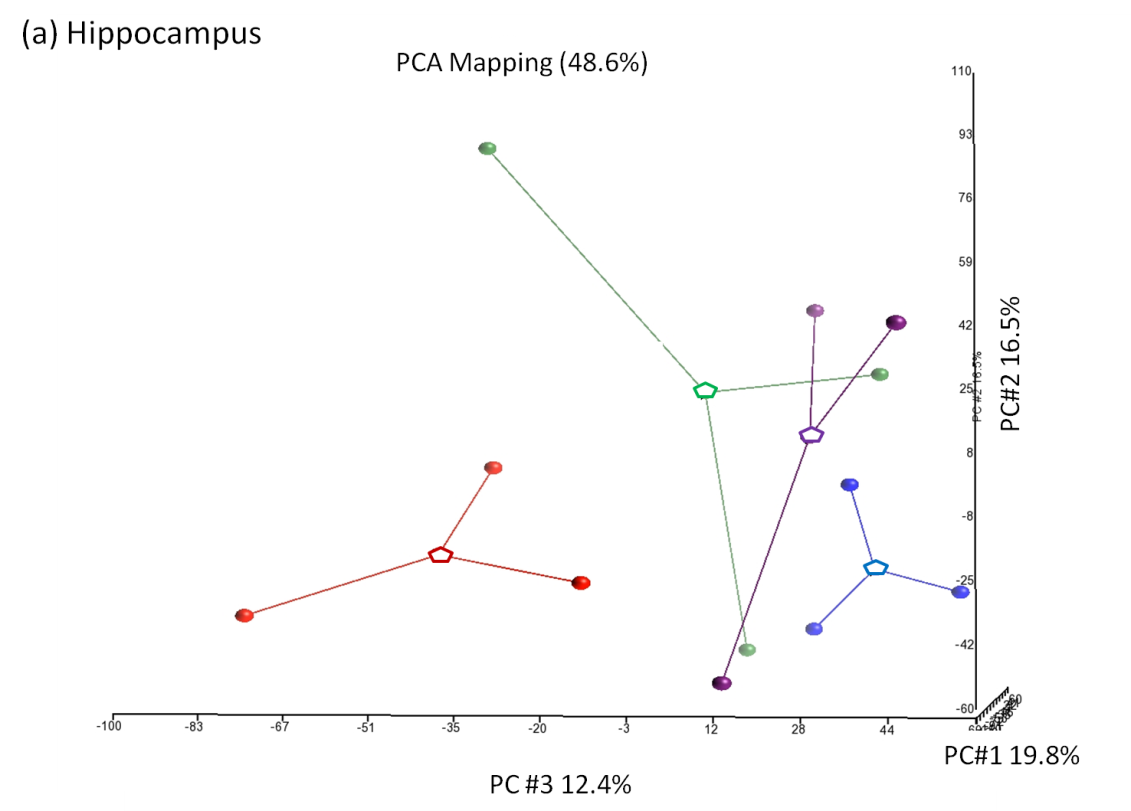


**
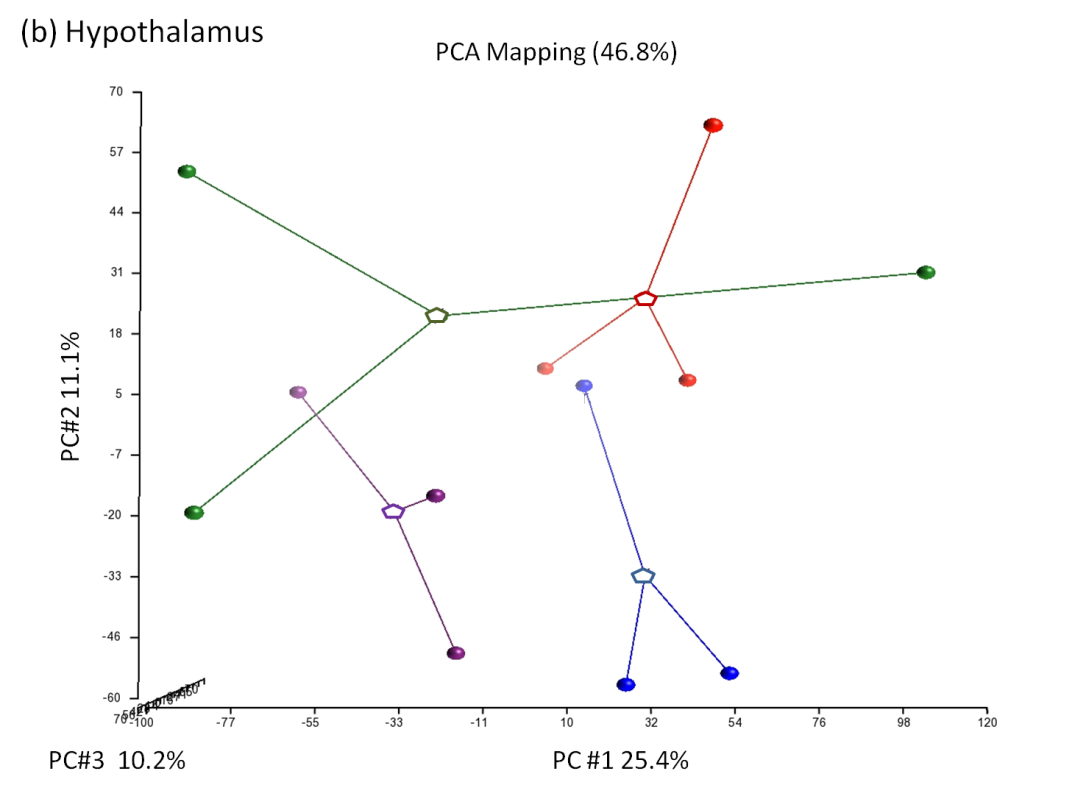
**

**Fig. S3.** Principal Component Analysis (PCA) plots of RNA-seq samples using normalized counts [log2 (normalized count + 32)] in the (a) hippocampal samples (n = 12) and (b) hypothalamic samples (n = 12). Data are clustered by treatment groups (using the centroid function). Red circles = CC; blue circles = BC; green circles = CB, and purple circles = BB. PC #1 = first component, PC #2 = second component; PC #3 = third component; % values measure the variation explained by each principal component. The cluster centroids (represented by pentagons) are theoretical and do not represent real samples.


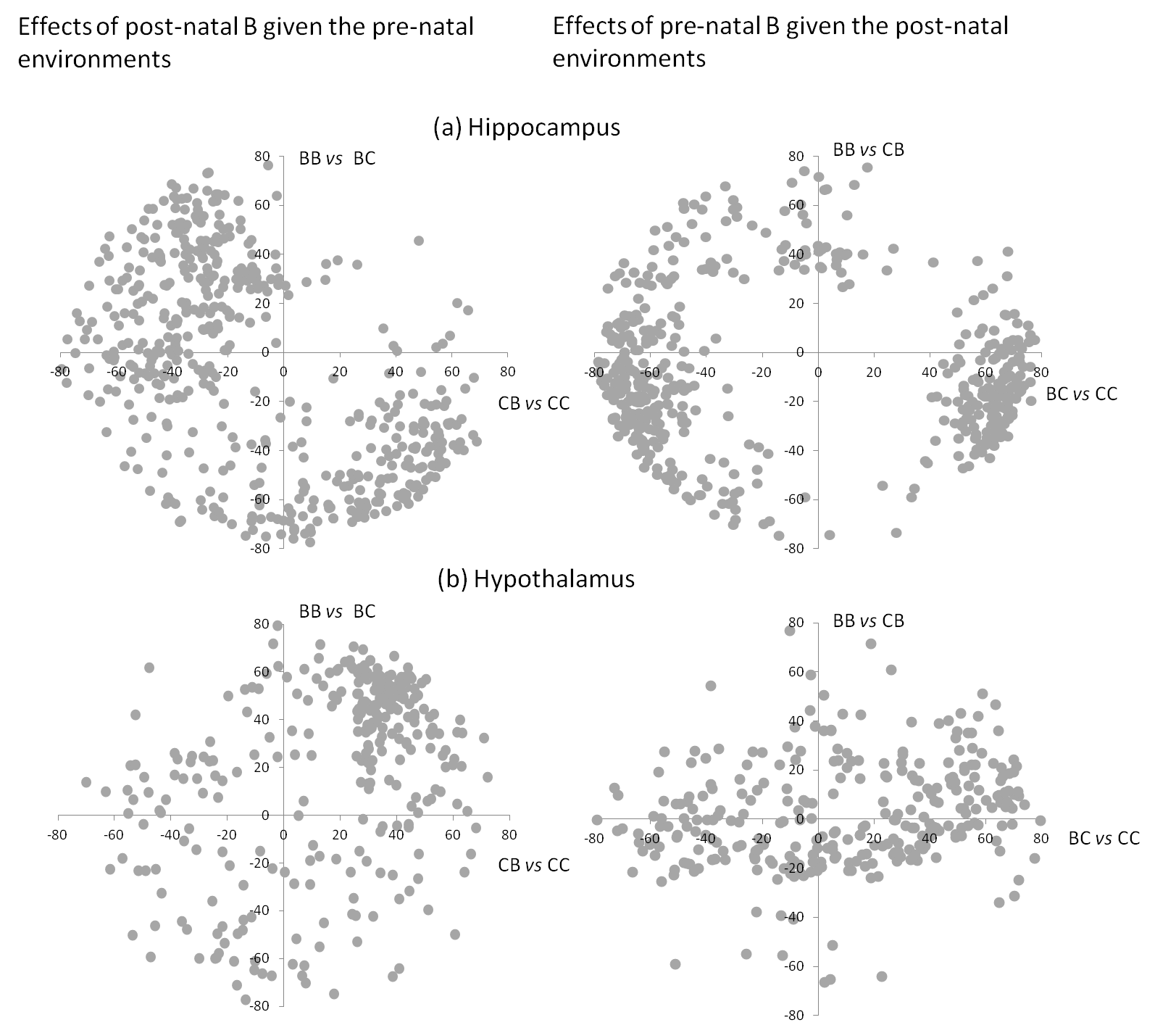


**Fig. S4** Graphical representation of Vector Analyses (full details on the two analyses in Fig. 1, Material and Methods) in the (a) hippocampus and (b) hypothalamus before partitioning and filtering the data according to specific gene behavioral categories (full details in Material and Methods). On the two axes are shown the sum vectors length for pre-natal or post-natal control (CB *vs* CC, and BC *vs* CC, respectively) and pre-natal and post-natal B (BB *vs* BC, and BB *vs* CB, respectively).


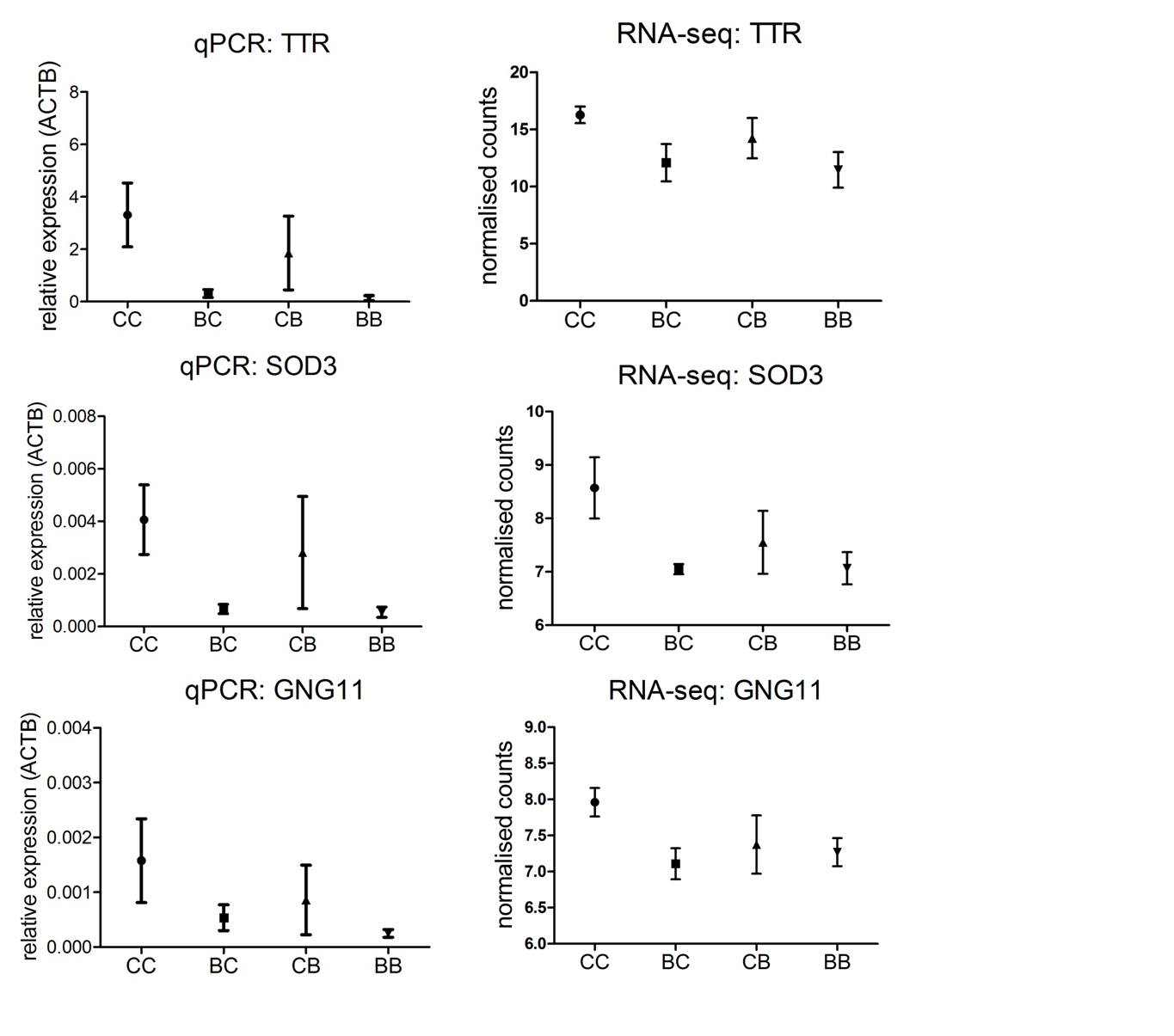


**Fig. S5.** Expression values for the genes TTR, SOD3, and GNG11 from qPCR and RNA-seq.


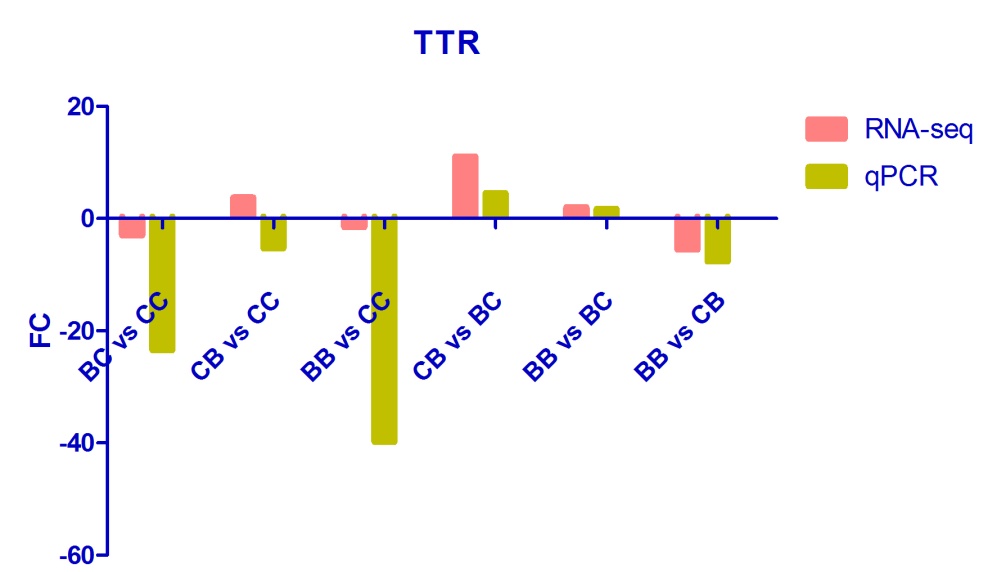


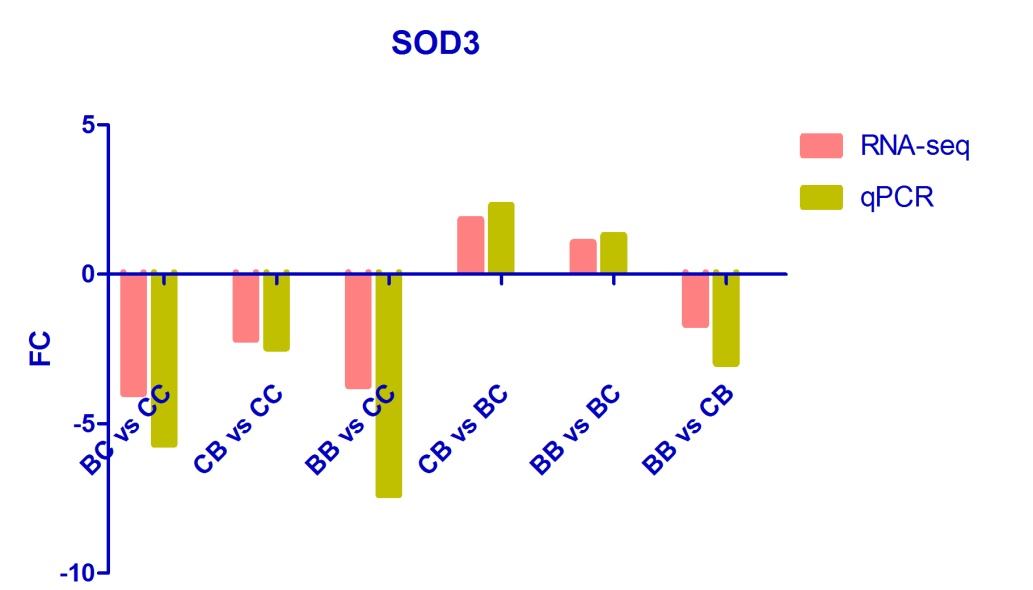


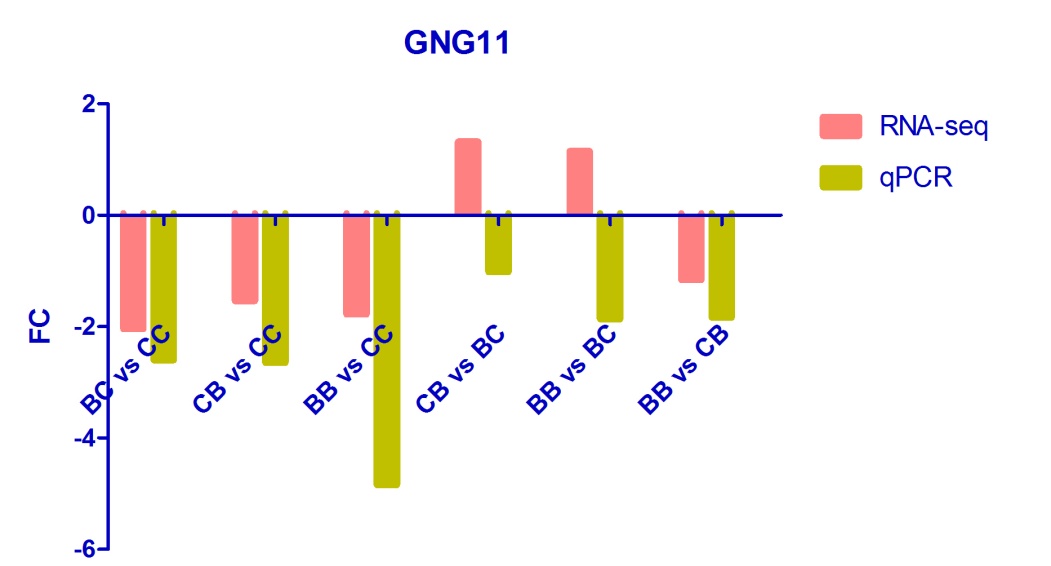


**Fig. S6.** Fold changes (FC) for the genes TTR, SOD3 and GNG11 derived on the basis of samples processed using RNA-seq and qPCR across the 6 pair-wise comparisons. Plotted values represent expression averages from the 3 biological replicates.
